# Computational based design and tracking of synthetic variants of Porcine circovirus reveal relations between silent genomic information and viral fitness

**DOI:** 10.1038/s41598-021-89918-6

**Published:** 2021-05-19

**Authors:** Lia Baron, Shimshi Atar, Hadas Zur, Modi Roopin, Eli Goz, Tamir Tuller

**Affiliations:** 1 Department of Biomedical Engineering, Tel-Aviv University, Tel Aviv, Israel; 2 SynVaccine Ltd. Ramat Hachayal, Tel Aviv, Israel

**Keywords:** Molecular evolution, Biomedical engineering, Computational biology and bioinformatics, Systems biology

## Abstract

Viral genomes not only code the protein content, but also include silent, overlapping codes which are important to the regulation of the viral life cycle and affect its evolution. Due to the high density of these codes, their non-modular nature and the complex intracellular processes they encode, the ability of current approaches to decipher them is very limited. We describe the first computational-experimental pipeline for studying the effects of viral silent and non-silent information on its fitness. The pipeline was implemented to study the Porcine Circovirus type 2 (PCV2), the shortest known eukaryotic virus, and includes the following steps: (1) Based on the analyses of 2100 variants of PCV, suspected silent codes were inferred. (2) Five hundred variants of the PCV2 were designed to include various ‘smart’ silent mutations. (3) Using state of the art synthetic biology approaches, the genomes of these five hundred variants were generated. (4) Competition experiments between the variants were performed in Porcine kidney-15 (PK15) cell-lines. (5) The variant titers were analyzed based on novel next-generation sequencing (NGS) experiments. (6) The features related to the titer of the variants were inferred and their analyses enabled detection of various novel silent functional sequence and structural motifs. Furthermore, we demonstrate that 50 of the silent variants exhibit higher fitness than the wildtype in the analyzed conditions.

## Introduction

Viral genomes, in particular coding regions, determine not only the protein products of a virus, but also how their production is regulated such as the regulation of viral gene expression, the replication of the viral genetic material, and avoidance of the immune system^1–5^. This regulation is encoded by the different combinations of synonymous codons, forming an overlapping second layer of information.

For example, it is known that synonymous codons can have different translation efficiencies and therefore affect the ribosomal speed^6^. The thermodynamic stability of the messenger RNA (mRNA) is under selection as well^7^, and the mRNA's secondary structure affects gene expression by a variety of mechanisms such as: the initiation of translation taking place at the mediated 5' untranslated region^8^, riboswitches^9^, recognition by the Protein kinase RNA-activated^10^,and activation of post-transcriptional silencing^11^. The structure of the mRNA can also influence the folding of the evolving amino acid (AA) chain by causing ribosome pausing^12^ and, in some cases, change the protein sequence by causing ribosomal slippage^13^. It has been shown that some of the secondary structures taken by RNA viral genomes are under powerful selective pressure because they regulate translation^12,14–16^, control replication initiation^17^, and constitute a target to cellular RNases^18^.

Selection acting on overlapping codes in the coding region is expected to be stronger in viruses with high compact genomes because their shorter genome should include various regulatory signals which must overlap with the protein-coding sequences. In this paper we focus on Porcine Circovirus type 2 (PCV2), which is a non-enveloped, isometric virus, 16–21 nm in diameter that contains a covalently closed, circular, single-stranded DNA genome. It belongs to the family *Circoviridae*, genus *Circovirus*^19,20^. Four PCV species have been identified so far: PCV1, PCV2, PCV3 and the recently identified PCV4^21^. PCV1 is non-pathogenic to pigs^20^ and was first identified as a cell culture contaminate in the mid-1970s by a German research group^22^. PCV2 was identified in pigs in Canada in the mid-1990s^23^. PCV2 is related to many diseases in piglets including wasting, respiratory signs and increased mortality^24^. In 2015, a novel Porcine Circovirus was identified in the USA and was designated PCV3. PCV3 is associated with reproductive failure, abortion and Porcine Dermatitis and Nephropathy Syndrome (PDNS)^25^. In 2019, a new Porcine Circovirus was identified in China in pigs with different health conditions and was designated PCV4^26^. All four PCV species have a similar structure: a single stranded DNA circular genome that includes two main open reading frames (ORFs) turning to opposite directions^21^.

PCV1 and PCV2 have similar initiation and termination signals at comparable locations in their genomes; they are different from each other with specific RNA expression levels and with a splicing selection unique to each virus^27,28^. There remain many unknowns regarding PCV3 and PCV4 since both were isolated recently recen^26,29^.

PCV2 has four main genotypes based on sequence analyses: PCV2a, PCV2b, PCV2c and PCV2d^30^. Two new genotypes were also recently proposed^31,32^. Sequence variations are mostly found in the capsid gene^33^. There are multiple lines of evidence of *in-vivo* recombination and co-infection that include several PCV2 genotypes—a within-host diversity of PCV2 quasispecies, demonstrating the role of the host's immune response in PCV2 evolution^34,35^.

In the past 20 years, PCV2 has arisen as a serious pig pathogen worldwide. Although DNA viruses are expected to be rather conserved, samples of PCV2, which is a small single-stranded DNA virus, display significant genetic variation and maintain evolutionary dynamics close to single-stranded RNA viruses^36–38^. The rate of its nucleotide substitution has been estimated as 1.2 × 10^–3^ substitutions per site per year—the highest registered substitution rate for a single-stranded DNA virus^39^.

PCV2 exhibits a rather complex transcription pattern. RNA synthesis is performed by the cell's enzymes after the single stranded viral genome is converted to a double stranded genome in the new host. The transcription is bidirectional (some of the genes are coded on the sense strand and some on the complementary one) and different RNAs are produced using alternative splicing.

The origin of replication (Ori) of the sense strand is a stem-loop structure. Four hexamer sequences (H1, H2, H3 and H4) are located downstream from the stem-loop^40^. The stem-loop structure has an important role in the termination of replication^40,41^, and the hexamers H1 and H2 are essential for the replication initiation since they form the Rep and Rep' proteins binding sites^40,42^. H3 and H4 are other optional binding sites for the Rep protein^43,44^.

The genome of PCV2 contains at least four open reading frames (ORFs). ORF1 encodes two proteins that play a crucial role in the replication cycle called Rep and Rep'^28,45^, ORF2 encodes the capsid protein^46^, ORF3 encodes a protein associated with apoptosis^47^ and ORF4 encodes a protein with a role in upregulating caspase activity and downregulating the activity of CD4^+^ and CD8^+^ T lymphocytes^48^. ORF1 and ORF2 are the two main ORFs, together constituting most of the viral sequence, and of these, ORF1 is reported to be more reserved^49–51^. It is believed that the PCV2 genome includes additional functional ORFs^49,52–56^.

In addition to Rep and Rep', ORF1 was also found to encode a group of RNAs and minor non-structural proteins (NS) associated RNAs (Rep3a, Rep3b, Rep3c, NS0, NS515, NS672). All of the RNAs mentioned share common 5' and 3' sequences, indicating that they are probably derived from the full rep RNA using alternative splicing^57^. Experiments inserting different mutations (start codon alteration, splice-junction modification and in-frame termination) into these RNAs concluded that only Rep and Rep' are required for PCV2 DNA replication^28,47,58^. The proteins encoded by Rep, Rep', cap, ORF3-RNA, ORF4-RNA, have been characterized. However, it is not clear yet what functions the minor RNAs have in the life cycle of the PCV^59,60^. The capsid protein encoded by ORF2 encapsulates the single-stranded DNA to construct infectious virions, it may also play a role in bringing the proteins Rep and Rep' from the cytoplasm into the nucleus for the DNA replication^50,61^.

In this study, we demonstrate a novel experimental computational approach for studying complex viruses such as PCV2. The approach includes the generation of a viral library of PCV2 which is designed and analyzed based on novel computational models and algorithms.

## Results

### Outline of the research

Five hundred variants of the PCV2 were designed to include various smart mutations (see Methods section). Using state of the art synthetic biology approaches, and in collaboration with the company “Twist Bioscience” (https://www.twistbioscience.com/), the genomes of these variants were generated. Competition experiments between the variants were performed in PK15 cell-lines. The variant titers were analyzed based on novel NGS experiments.

**Figure 1:**
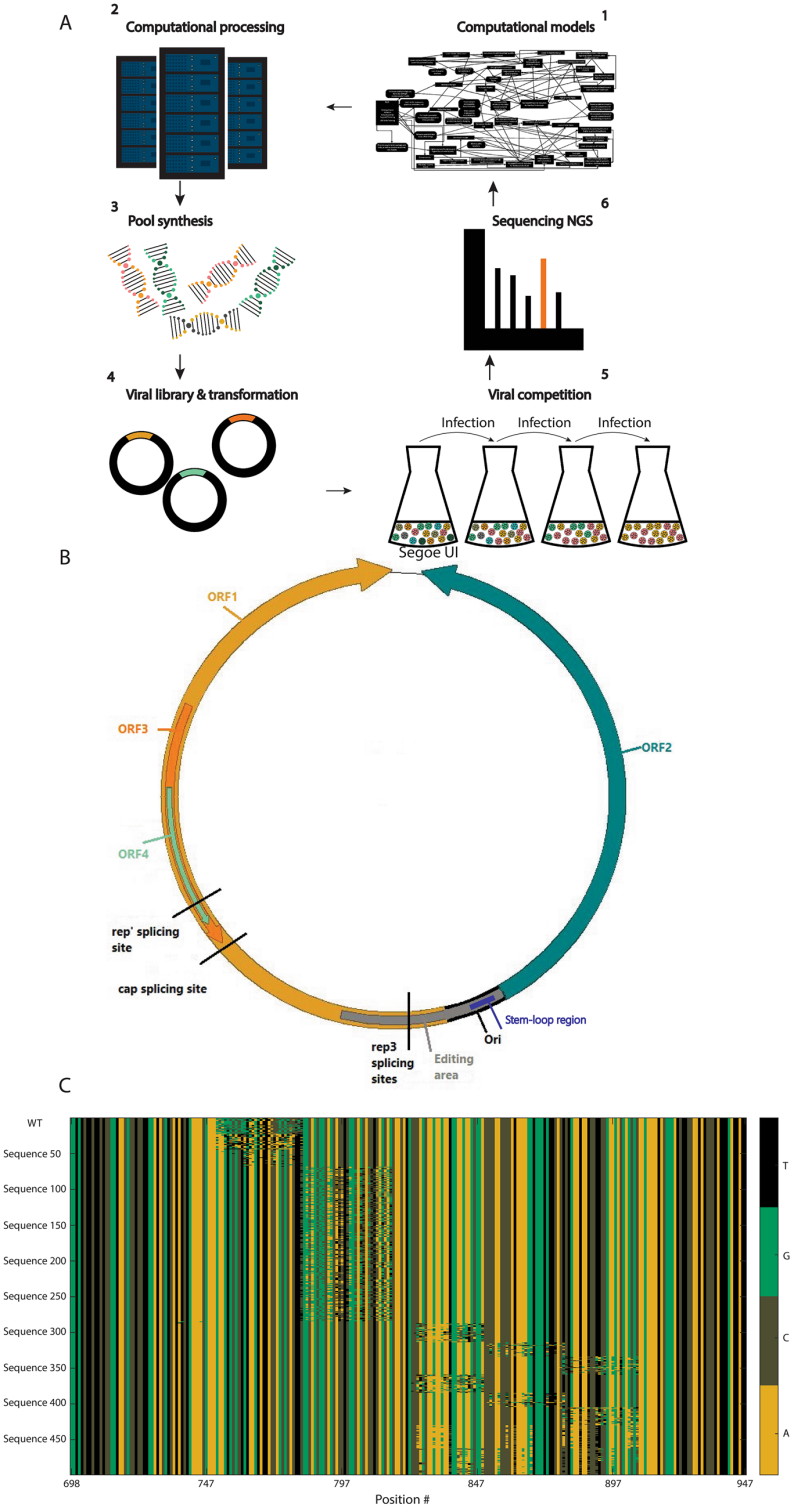
(**A**) Flow diagram of the study: 1. Building computational models 2. Simulating/implementing the models on powerful servers 3. DNA pool synthesis 4. Generating live viral libraries. 5. Viral competition. 6. NGS and Read Count (RC) analysis. (**B**) The genome of PCV2 with ORF1—ORF4 marked. In gray—the edited region in the synthetic library (nucleotides 738 – 947 according to NCBI accession number KJ128273). This region includes the Ori (origin of replication, marked in a black region) and the beginning of ORF1. The Ori region includes the stem-loop structure, marked in blue. The splicing sites of rep', rep3 and cap are marked in black lines. (**C**) A heatmap diagram of the DNA sequences in the oligonucleotide library (nucleotides 698 – 947 according to NCBI accession number KJ128273) of the five hundred variants in the synthetic library. This region includes the edited region with an inception of 40 nucleotides identical to the wildtype (see Methods section); the heatmap was generated by Matlab.

The general stages of the research are as follows: (1) Creation of computational models and feature selection based on big data—testing thousands of wildtype (WT) variants of viruses. The models enabled designing specific effective mutations relative to the wildtype DNA. (2) Creation of a pool of short pieces of synthetic DNA. (3) Replacing segments of DNA within the virus using recombination to generate a pool of viruses. (4) Transfecting relevant cells with the pool of engineered viruses and allowing them to replicate. The viruses are harvested and stored for subsequent passages. This procedure (transfection → replication → harvesting → storage) is repeated for several passages (generations) and aliquots from each passage are stored for later titration and sequencing. (5) Extracting viral DNA from aliquoted supernatants taken at different generations. Amplifying the DNA using polymerase chain reaction (PCR) and sequencing using NGS. (6) The relative titer of each variant allows a deduction on the fitness of the synthetic variant to its host. Insights can be re-implemented in the models (step 1). See illustration of the research in Fig. 1A and illustration of the virus in Fig. 1B.

### Various novel functional codes in the PCV genome

**Figure 2:**
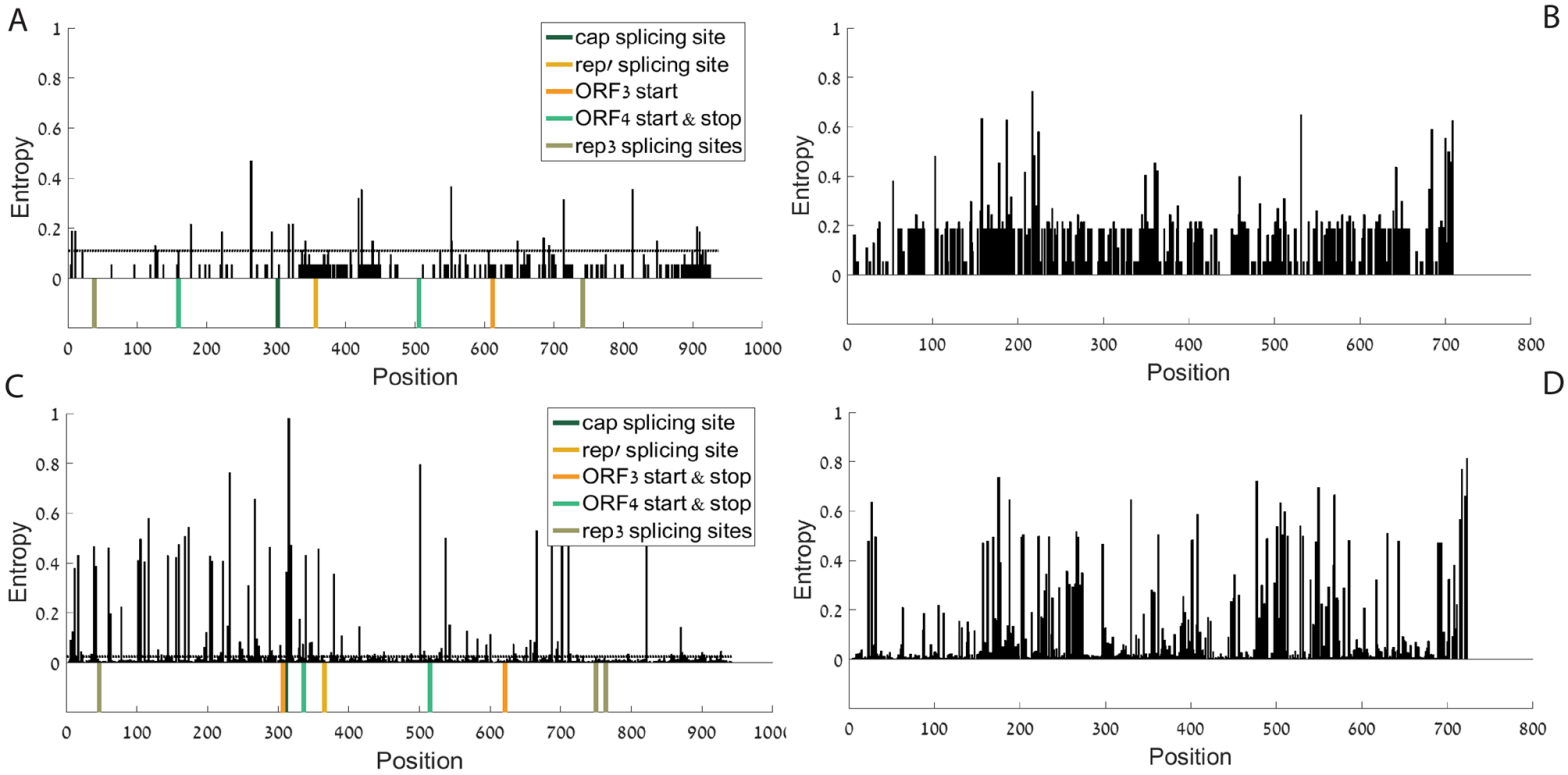
Entropy per AA aligned position: (**A**) PCV1 ORF1; (**B**) PCV1 ORF2 (**C**) PCV2 ORF1; (**D**) PCV2 ORF2. The entropy in PCV2 is higher than in PCV1. The entropy in ORF2 is higher than in ORF1 in both PCV1 and PCV2. Analyses of PCV1 were done based on coordinates of NCBI accession number AY184287^58^, analyses of PCV2 were done based on coordinates of NCBI accession number AY094619^57^.

After downloading 2091 wildtype PCV variants from databases (68 PCV1 variants 2023 PCV2 variants, see details about the downloading dataset and date in Methods section), each of the two major genes was aligned at the amino acid level. Each multiple sequence alignment (MSA) was analyzed to calculate an entropy score for each position (see Methods section). Entropy measures the conservation at the nucleotide level and was computed for each position along the coding sequence. Higher entropy is related to lower conservation and the minimal entropy (zero) is related to the 100% conservation. As shown in Fig. 2, ORF1 has lower entropy than ORF2 in both PCV types (PCV1 and PCV2), confirming as expected that in general ORF1 is more conserved than ORF2. It seems that positions with regulatory signals that are located in ORF1 such as splicing sites and start/stop sites on inner genes, tend to have relatively low entropy (as expected) as they are all in the top 2%/11% most conserved among all the positions in the PCV2/PCV1 genomes respectively (see dotted lines in Fig. 2A–C marking these thresholds).

**Figure 3:**
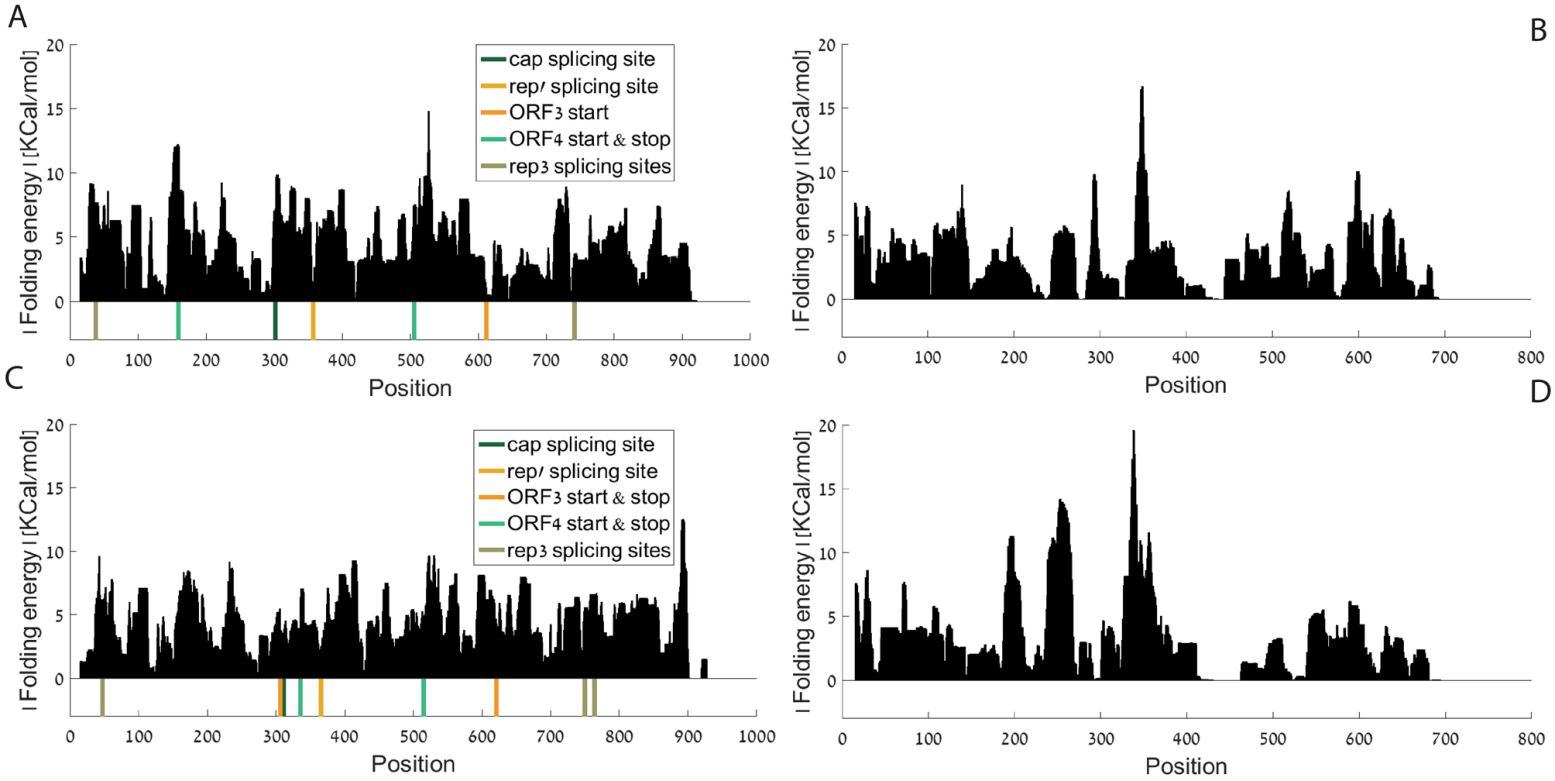
Absolute folding energy per AA aligned position, window size = 31: (**A**) PCV1 ORF1; (**B**) PCV1 ORF2 (**C**) PCV2 ORF1; (**D**) PCV2 ORF2. Analyses of PCV1 were done based on coordinates of NCBI accession number AY184287^58^, analyses of PCV2 were done based on coordinates of NCBI accession number AY094619^57^.

As can be seen in Fig. 3, absolute folding energy (FE) analyses show that the Rep' splicing site is in a site with relatively low absolute folding energy in both PCV1 and PCV2. Rep’ splicing sites rank in the 9th and 19th percentiles in regards to folding energy in PCV1 ORF1, and PCV2 ORF1 respectively, meaning that this site is placed in a position with a relatively open local structure. As already mentioned in the introduction, previous studies showed that Rep and Rep' are the essential proteins for viral DNA replication.

An average on the folding energy analysis also shows that PCV2 ORF2 has a long "open" (non-folded) segment between nucleotide no. 400 and nucleotide no. 500 from the start codon of ORF2 (nucleotides 1336 – 1236 on the complimentary strand, according to NCBI accession number AY094619). This is a segment with an extremely low folding strength in comparison to the rest of the genome (z-score = -2.0451).

Further folding energy analyses with different window sizes appear in Supplementary Figs. S1–S3. PCV1 ORF2 shows a similar structure at ~ 450 to ~ 500 nucleotides from the start codon of ORF2 (nucleotides 1274 – 1224 on the complimentary strand, according to NCBI accession number AY184287).

**Figure 4:**
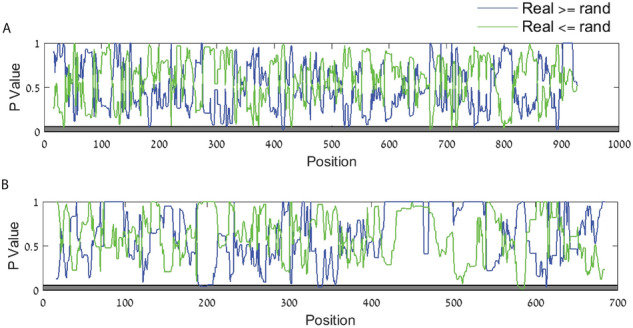
*P* values vs. position of the folding energy compared to randomized sequences. In blue—*P* value related to the probability of the real genome to be higher than or equal to randomized version, in green—*P* value related to the probability of the real genome to be lower than or equal to randomized version, in dark gray—the statistically significant region (*P* value <  = 0.05). (**A**) KJ128273 ORF1 (**B**) KJ128273 ORF2.

A comparison of a wildtype PCV2 viral sequence (NCBI accession number KJ128273) to 1000 corresponding randomized variants generated by the randomization model (see Methods) was performed. Absolute folding energy analysis with different window sizes shows that the long "open" segment (which is 150 nucleotides long in this strain) is not fully conserved/preserved/present in any random model, yet using our null model we were not able to show that this pattern is not due to the amino acid content in this region (Fig. 4).

We used a statistical null model that generates random genomes of the virus with exactly the same amino acids, but with different codon order (see Methods). We could not show that in the null model the signal is weaker. This means that we can't claim that the patterns are not due to the amino acid content in this region. This implies that one of the following is true: (1) the selection for amino acid content induced the signal but the AAs were selected due to other reasons, (2) The AAs in this region were selected for directly, to generate this pattern, (3) The pattern is not only due to the AAs content in this region but we do not have sufficient or appropriate data to demonstrate this.

Further analyses for ATG context, empirical *P* values for codon distribution for each amino acid, codon adaptation index (CAI) and effective number of codons (ENC) are described in Supplementary Figs. S4–S7, Supplementary Tables S2–S3). These additional overlapping codes can be studied via the method described in the next sub-sections.

### Properties of the PCV library titer

To study the novel, possibly overlapping codes that appear in the genome, we designed a library of five hundred PCV2 variants which included the following:

- Increasing or decreasing GC content in the stem-loop.
- Increasing or decreasing the loop length by removing/adding 1–2 connections at the end of the stem.
- Inserting point mutations in 6mer motifs^40,41^ downstream to the stem-loop.
- Decreasing the folding energy at the beginning of ORF1 while maintaining the AA sequence.
- Replacing blocks with the most frequent codons/AAs that appear in a column in the alignment of the wildtype PCV2 genome in the first 42 nucleotides of ORF1.
- Replacing sub-sets of the first 14 codons of ORF1 with the most frequent codons that appear in various cell line transcriptome (HEK, Hela, and NCI1299 cells respectively).
- Various combinations of the mutations above. More details are provided in the Methods section.

In the first step, a 250 base oligonucleotide library encoding these variants was generated using massively parallel on-chip DNA synthesis (Twist Bioscience: https://www.twistbioscience.com; Methods).

**Figure 5:**
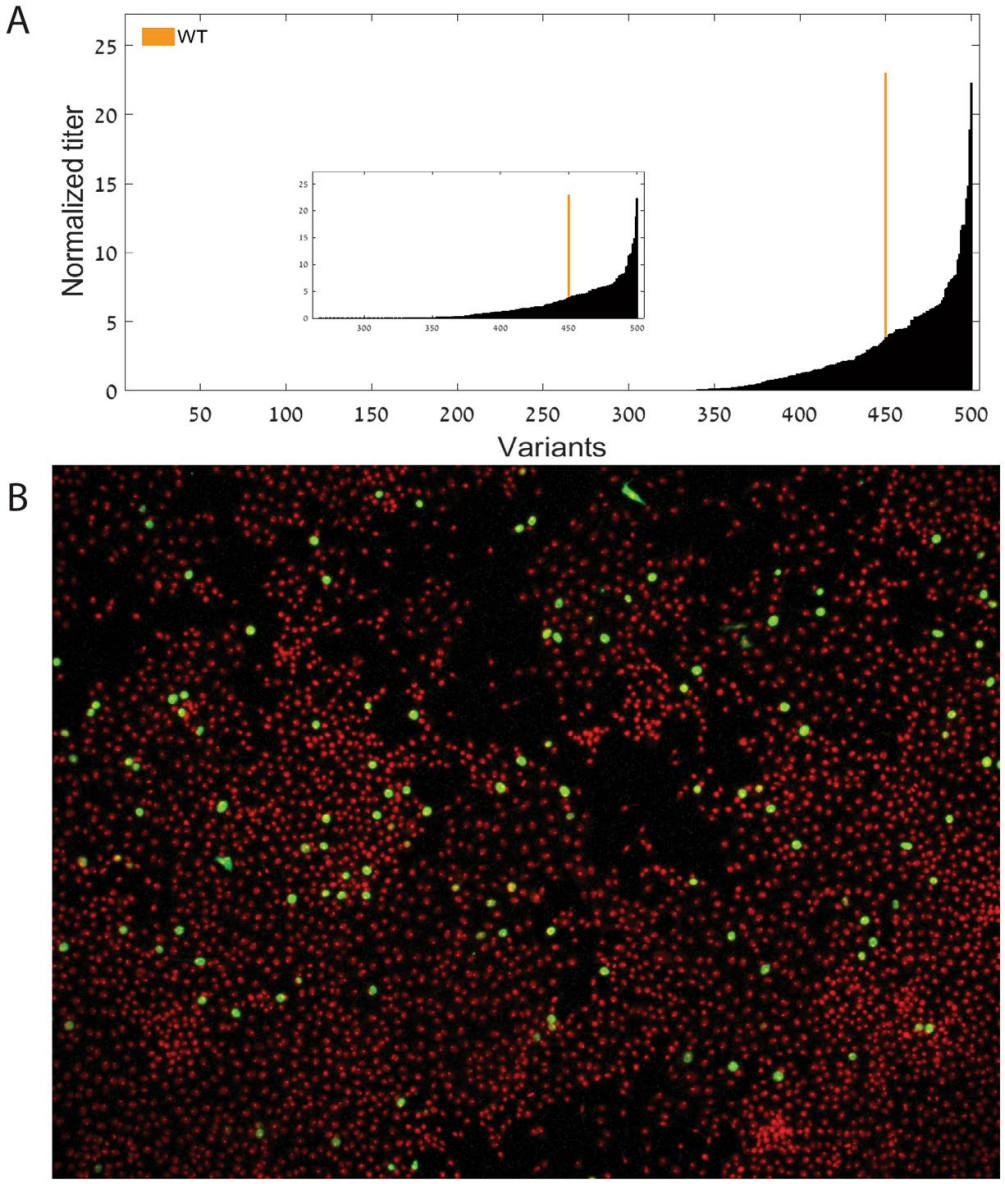
(**A**) Normalized titer (read counts) of the different variants in the PCV2 library sorted by their relative read counts. The wildtype's location is marked in orange. (**B**) FFA example (the cells are in red and viruses are in green).

The pool of DNA was used to generate a pool of viruses in PK15 cells which undergo a few passages (Methods section) and the titer of the viruses was then compared via novel NGS approach. The approach included sequencing the variable region among the variants with the titer estimated based on the number of reads mapped to each variant (more details in the Method subsection). As can be seen in Fig. 5A, the estimated titer of the viruses spans 6 order of magnitudes with 50 variants with estimated higher titer than the wildtype and 266 variants with titer which is very low and close to zero. Some of the variants were evaluated based on focus-forming units per milliliter (FFA, Methods), see Fig. 5B.

### Modeling the titer of a PCV library

In the next step, we aimed at modeling and understanding the PCV genomic features that affect the titer of the PCV variants in our experiment.

**Figure 6:**
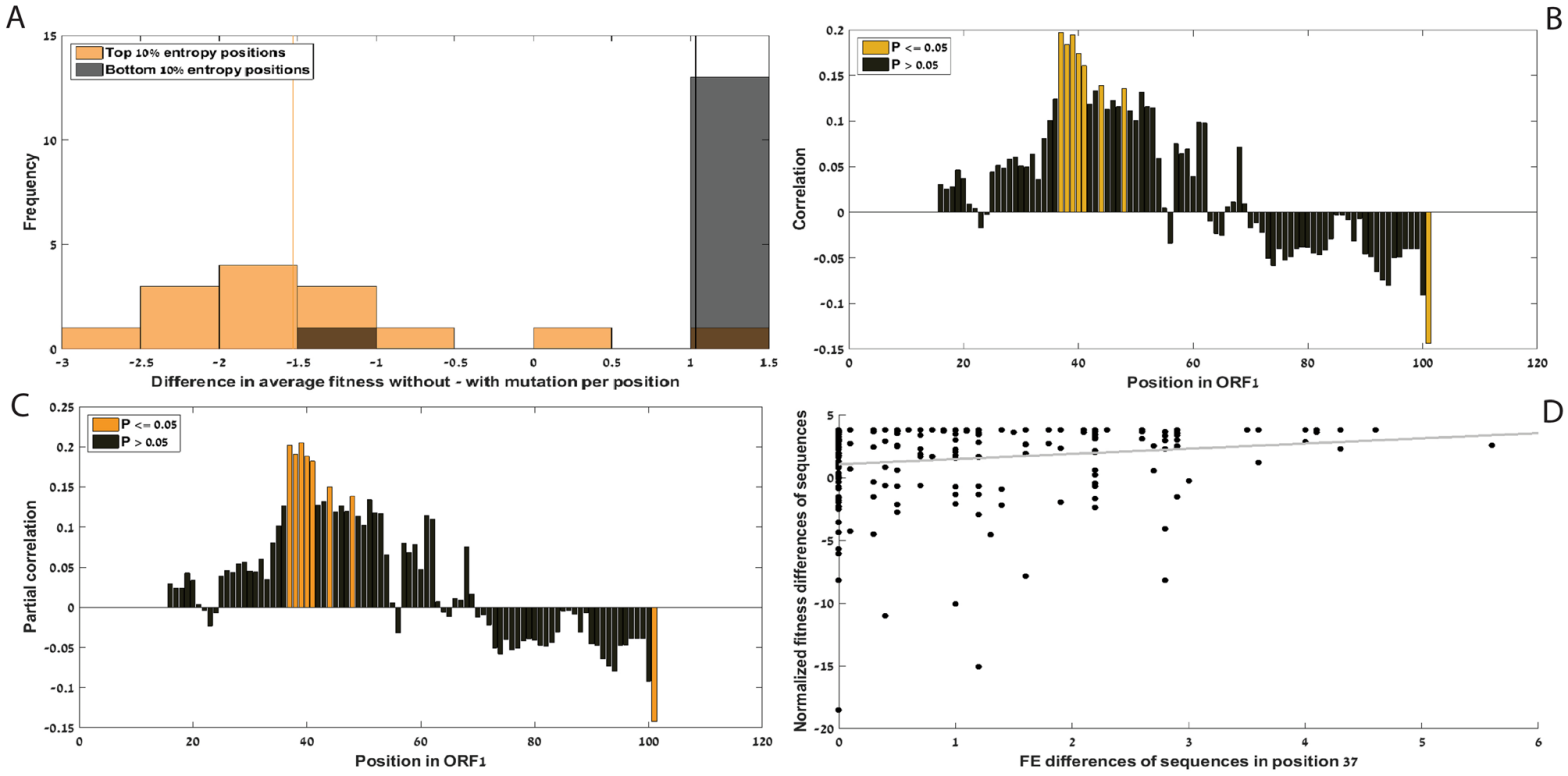
(**A**) Differences in average fitness due to mutation (fitness without mutation minus with mutation) for high and low entropy variants (*P* value = 2.3430e−04); see main text). The two distributions are clearly different with different medians (marked in thin lines) and spreads. (**B**) Spearman’s correlations between folding energy differences and fitness difference from wildtype vs. position in ORF1. (**C**) Spearman’s partial correlations between folding energy differences and fitness difference from wildtype given the number of mutations in the ORF vs. position in ORF1 (**D**) Fitness differences vs. folding energy differences of ORF1 mutated sequences compared to the wildtype in position 37 nucleotides from the start of ORF1 (ρ = 0.1971, *P* value = 0.0039).

Figure 6A shows the histograms of differences in average fitness without *vs.* the fitness *with* mutation in a certain position. There are two histograms: (1) mutation positions with top entropy based on the PCV2 wildtype genomes (i.e. lower conservation) and (2) mutation positions with lowest entropy (i.e. highest conservation). As can be seen, the two distributions are clearly different with different medians and spreads.

For conserved positions (low entropy, gray bars), a mutation in the position tends to have a greater difference between the fitness of the wildtype and the fitness of the mutant, due to a greater decrease in the fitness caused by the mutation. The mutations in the non-conserved positions (high entropy, orange bars), tend to have a lower effect on fitness. This demonstrates that conserved positions are conserved due to selection (the conserved positions are important for the viral fitness) and not because of (random) genetic drift.

Figure 6B shows Spearman’s correlations between folding energy differences (in absolute values) and fitness difference from the wildtype *vs.* position in ORF1. Correlation values that are statistically significant are marked in yellow. A positive correlation suggests that there is a statistical relationship between the changes in the local folding energy in the position and between lowering the fitness of the variant compared to the wildtype. Approximately 40 nucleotides from the start of ORF1 there is an area of statistically significant positive correlations. This is an area where the mRNA of ORF1 is spliced in order to create short RNAs (Rep3a, Rep3b and Rep3c). The role of these 3 short RNAs is yet unclear^57^. We learn from this analysis that the local mRNA folding energy of PCV2 has a significant effect on its fitness.

Figure 6C shows Spearman’s partial correlations between folding energy differences and fitness difference from wildtype given the total number of mutations in ORF1 of the variant *vs.* position in ORF1. Orange bars indicate statistical significance. Figure 6B and Fig. 6C are almost identical, indicating that the total number of mutations in the ORF can’t explain the result, supporting the conjecture that the correlation is directly related to local mRNA folding. The conclusion is that when designing a variant with different regulatory coding (in this case the folding energy) in the regulatory positions/areas mentioned above, the fitness of the variant is expected to decrease.

Figure 6D shows fitness differences *vs.* folding energy differences of ORF1 mutated sequences compared to the wildtype in position 37 in the genome (counted in nucleotides from the beginning of ORF1), which is the position with the highest correlation in Fig. 6B.

Next, we aimed at evaluating the ability to predict the fitness of PCV2 variants in our synthetic environment based only on their genomic sequence features. To achieve this, we trained a regressor based on various sequence features such as DNA and mRNA folding energy changes, DNA and mRNA topological distance and mutations in different positions (see Methods for more details). The regressor was trained based on part of the data (60%) and was validated based on the rest of the data (see details in the Methods section).

**Figure 7:**
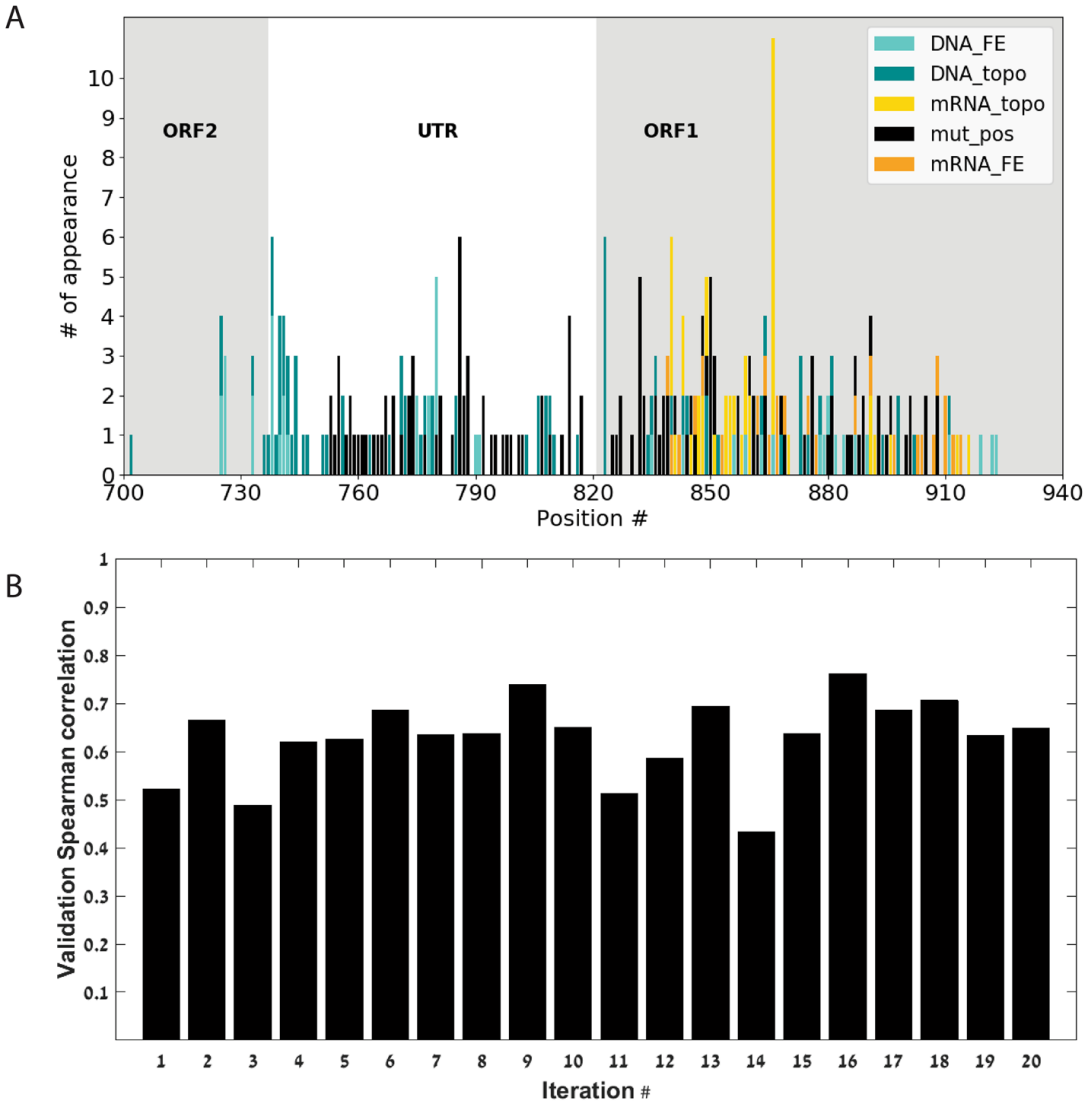
(**A**) The number of times a feature entered into the model out of 20 randomizations vs. the nucleotide position to which the feature refers, according to the feature groups: DNA folding energy difference (DNA_FE in turquoise); DNA topological distance (DNA_topo in dark cyan); mRNA topological distance (mRNA_topo in yellow); Mutation in position (mut_pos in black); mRNA folding energy difference (mRNA_FE in orange). (**B**) Spearman correlation of the validation set at each run of the fitness predictor. All correlations are statistically significant (*P* value < 0.05).

Figure 7A shows the number of times a feature was selected by the model (out of 20 iterations which include randomized division of the data to a training set, a test set and a validation set) and Fig. 7B includes the performances of the predictor. The top features are described in Table 1.

**Table 1:** The most frequently selected features that entered the model and their correlation with the estimated fitness.

Name	Number of appearance (out of 20)	Spearman correlation	*P* value
Topological distance in the mRNA in position 865 ('mRNA_topo_dist_865')	10	0.6824	8.534e−70
Bend angle difference in the DNA in position 822 ('DNA_topo_dist_BendAngle_pos_822')	6	0.0435	0.3321
Mutation in position 785 ('mut_pos_785')	6	− 0.1435	0.0013
Folding energy difference in the DNA in position 779 ('DNA_FE_diff_779')	5	0.1675	0.0002
Mutation in position 831 ('mut_pos_831')	5	0.1240	0.0055
Mutation in position 849 ('mut_pos_849')	5	0.0325	0.4685
Folding energy difference in the DNA in position 737 ('DNA_FE_diff_737')	4	− 0.1593	0.0003
Topological distance in the mRNA in position 839 ('mRNA_topo_dist_839')	4	0.5965	1.64e−49
Mutation in position 813 ('mut_pos_813')	4	− 0.3942	4.89e−20

There were no variants that had mutations in ORF2. However, the structure of the genetic material can include nucleotide base pairing where one nucleotide is in ORF1 and the second in ORF2; thus, modifying ORF1 can affect the base paring of nucleotides in ORF2. According to the folding predictions, the mutations downstream of ORF2 change the folding and the topological structure in that area.

The most frequently selected features highlight important positions and areas (known and yet unknown) within the PCV's genome. For example:

- "mut_pos_785" (6/20) which refers to a mutation in a position within the H1 hexamer that is an essential binding site for Rep and Rep' proteins that, as mentioned before, are essential for the PCV2 viral replication.
- "DNA_FE_diff_779" (5/20) refers to folding energy increase or decrease in a position within the stem-loop. The stem-loop is a highly important functional structure crucial for the initiation and termination of the DNA's viral replication.
- "DNA_FE_diff_737" (4/20) refers to folding energy changes right at the beginning of ORF2, which indicates that although mutations were not inserted in the ORF2 region, changes in the area close to it influence its structure and folding in the DNA form.
- "mut_pos_813" (4/20) refers to a mutation in a position within the H4 hexamer that is an additional optional binding site for the Rep protein.

This analysis enables us to plan the features in the next generation of the synthetic library, and is an important tool within the flow of the model. Since we build a predictive model that connects features of the virus to its titer we now can use this model for generating new viruses with features that are predicted (by the model) to have high titer.

Figure 7B shows the Spearman correlation of the validation set at each run of the fitness predictor. The mean value of the correlation is 0.6283.

## Discussion

We describe the first computational-experimental pipeline for studying the effects of silent and non-silent genomic information on viral fitness. The pipeline was implemented to study the Porcine Circovirus type 2 (PCV2). Output results of the pipeline form the input for the computational modeling stage.

The first step of the study included analyses of thousands of wildtype PCV genomes. We were not able to detect selection for global codon bias; we believe that there are two major reasons for this lack of signal: (1) various interleaved codes (e.g. codes related to splicing, replication, and more) that appear in the coding regions of the PCV and constrain its evolution; and (2) The genome of PCV is too short to infer a statistically significant signal of codon usage bias.

However, we found many positions which seem to undergo selection for weak mRNA folding. For example, the Rep' splicing site is placed in an open local structure as was expected. This, in our opinion, improves the fitness of the virus since the spliceosome can splice the mRNA more easily. Rep' (in addition to the full length Rep) as mentioned before is known to be essential to the PCV's viral replication.

Our genomic analysis of PCV2 also discovered novel signals that may be important for viral fitness. For example, PCV2 ORF2 has a long 'open' segment (in terms of mRNA local folding) at nucleotides ~ 400 to  ~ 500 after the start codon of ORF2. It is possible, that this region includes additional unknown regulatory signals which require/induce weak mRNA folding. This result was reproduced by calculating local folding energy with different window lengths and it is not fully reserved in a null model. In addition, we confirmed that ORF1 is more conserved than ORF2. This is probably a consequence of ORF1 including more regulatory signals (such as splicing sites and start/stop sites on inner genes) encoded in silent aspects of the ORF, and not simply because the only proteins known to be essential to the virus' replication (Rep and Rep') are encoded by ORF1.

Further, we analyzed and modeled an estimation of the titer of synthetic PCV2 variants. The titer is related to the variants growth rate (or fitness) in the studied conditions. The variants were designed partially based on the genomic analysis of PCV2. We demonstrated that the variants in this synthetic library have a gradient of fitness spanning 6 orders of magnitude. The synthetic library includes 50 variants with higher titer than the wildtype and 266 variants with titer which is very low and close to zero.

Simple RNA and single-stranded DNA viruses tend to rely on a large variant diversity within the host in order to escape the host's immune system^62–65^. According to some studies, in-host viral quasispecies represent a single selection unit, where selection is not performed based on the individual variant's fitness, but according to the population's mutant distribution^66,67^. A recent study tested this hypothesis on PCV2 by monitoring PCV2 variability over time during an experimental infection suggesting an interaction between genetic heterogeneity, immune system response and disease severity^35^. Our research tested a synthetic version of the quasispecies hypothesis using PCV2 for several passages within cells, without the linkage to an immune system of a host. The fitness described in our work is restricted to the cell's biological machinery, focusing on the replication cycle.

We also showed that fitness in the synthetic library can be predicted based on genomic analysis of the wildtype PCV2 genomes. For example, mutations in positions with high conservation (low entropy) have a higher effect on the fitness in our library. Similarly, mutations that affect the local mRNA folding energy of PCV2 tend to have a higher effect on the fitness of the synthetic variants.

This result suggests that entropy should be taken into account when designing a synthetic virus. It connects the evolutionary selection/conservation levels of the wildtype viruses in nature and the fitness of the viral variants in the synthetic system created in the lab.

In the next step, we aimed at evaluating the ability to predict the fitness of the PCV2 variants in our synthetic environment based only on their genomic sequence features via training a regressor. The most frequently selected features highlight important positions and areas (known and yet unknown) within the PCV2 genome, like the four hexamers and the stem-loop at the origin of replication. The fact that many of the relevant features in the synthetic library overlapped with conserved features in the wildtype genomes suggest that our experiment can capture relevant features in the viral natural life cycle.

To summarize, this study demonstrates how combining genomic analyses of wildtype genomes that were shaped by evolution and the titer of synthetic genomes of viruses can be used for better understanding of the complex overlapping signals that appear in viral genomes. We believe that in the future, in addition to the understanding of the viral genomes, our approach can be used for designing new viruses to address specific objectives, such as vaccinations.

Our research has some limitations: first, due to technical reasons, our synthetic biology experiments were focused on a short (but interesting) region in the PCV2 genome. It would be worthwhile in the future to explore other parts of the PCV2 viral genome in a similar manner in order to further substantiate this study’s findings.

Second, our synthetic biology experiments were performed in cell lines. Thus, the fitness ranking of the variants is not related to aspects of the PCV2 life cycle such as interaction with the immune system and tissue penetration. We demonstrate that there is a correlation between the effect of sequence features on viral fitness in our experiments and the selection of these features in natural conditions; however, the correlation is far from being perfect.

Furthermore, there are previous studies that aimed to estimate the fitness and dynamics of PCV2 strains in the host (see, for example^35^). Currently it is very challenging to compare our approach to such studies as our synthetic viruses are completely different to naturally circulating strains that were shaped by evolution in pigs. Further research in which naturally circulating strains are generated synthetically and compared to our synthetic variants would enable better generalization of the conclusions reported here.

Lastly, in this study we mainly studied PCV2 as this is the genotype with the most abundant genomic information. However, we believe that at least some of the conclusions are relevant to the PCV3 and PCV4 genotypes too, as their genome has around 50% similarity to PCV2^25,26^. The approach described here could be applied to study other viruses (e.g. SARS-CoV-2) too.

## Methods

### Library design

The PCV2b engineered region is GGTGTCTTCTTCTCCGGTAACGCCTCCTTGGATACGTCATATCTGAAAACGAAAGAAGTGCGCTGTAAGTATTACCAGCGCACTTCGGCAGCGGCAGCACCTCGGCAGCACCTCAGCAGCAACATGCCCAGCAAGAAGAATGGAAGAAGCGGACCCCAACCCCATAAAAGGTGGGTGTTCACTCTGAATAATCCTTCCGAAGACGAGCGCAAGAAAATACGGGATCTTCCAATATCCCTATTTGATTATT (see Fig. 1B,C for further details).

The genomic coordinates of the region are 698–947 according to NCBI accession number KJ128273. We chose this region because we believed that it is populated with various interesting regulatory signals^40,68^. Note that the ends of the regions are constant for amplification with primers and for recombination into the viral genome.

We performed various mutations in the region aiming to consider the following aspects and generating 500 variants:

1. a. For all stem-loops:
  I. Increasing the gradient of GC in the stem-loop: replacing A-T with G-C in 1, 2, 3, positions (i.e. generating differences in up to 6 in GC content in this region).
  II. Decreasing the gradient of GC in the stem-loop: replacing A-T with G-C in 1, 2, 3, positions (i.e. generating differences in up to 6 in GC content in this region).
  III. Increasing/decreasing the loop by removing/adding 1–2 connections at the end of the stem-loop.
  b. Inserting point mutations in 6mer motifs downstream of the stem-loop (suspected to be related to the viral replication40), and calculating all permutations. In our PCV2b strains the Hmers motifs40 are as follows:
  $$\begin{aligned} & {\text{CGGCAG}}\,{\text{CGGCAG}}\,{\text{CACCT}}\,{\text{CGGCAG}}\,{\text{CACCT}}\,{\text{CAGCAG}} \\ & {\text{H}}1\quad {\text{H}}1\quad {\text{H}}2\quad {\text{H}}1\quad {\text{H}}2\quad {\text{H}}1{\text{m}} \\ \end{aligned}$$
  c. Point mutations in the unique region upstream of the stem-loop across all genomes.
2. ORF1 mRNA folding: increasing/decreasing the folding gradient for windows 30 nucleotides in length, in the first 87 nucleotides of ORF1, and selecting 4 samples from each variant of windows 1–30, 31–60, 58–87 across all genomes.
3. Replacing the first 87 nucleotides of ORF1 with the most frequent codon in an alignment of all the PCV2 genomes in this position.
4. Replacing codons synonymously with the most frequent codons in the host's transcripts in the first 87 nucleotides of ORF1—we use the Pig's transcriptome and chose the longest transcript to represent each gene to avoid.

The final sequences of all the variants appear in Supplementary Table S3.

### Cell lines

The porcine kidney cell line, PK15-C1 (PTA-8244) that is highly permissive for PCV2 replication was maintained in Eagle's minimum essential medium (MEM, Gibco) supplemented with 10% fetal bovine serum (FBS), streptomycin 0.1 mg/ml, penicillin 100 u/ml, nystatin 12.5 U/ml, 0.29 mg/ml at 37 °C under 5% CO_2_. PK15-C1 cells used in this study were confirmed to be free of PCV and porcine parvovirus contamination.

### Construction of a synthetic porcine circovirus type 2b (PCV2b) genome library

The background genome of PCV2b (accession number KJ128273 NCBI) was de novo synthesized using solid-phase DNA synthesis method (BioBasic). Additionally, an oligonucleotide library encompassing 500 variants, each corresponding to a single 250 base pair fragment in the edited region of the PCV2b genome (see subsection "Library design") was generated using massively parallel on-chip DNA synthesis (the technology of Twist Bioscience company; www.twistbioscience.com).

To construct the synthetic virus library, two amplicons, 1605 and 271 base pairs long, with overlapping sequences in the PCV2b-genome were prepared by low cycle PCRs (up to 23 cycles). PCR reactions (8 per fragment) were carried out using Q5 Hot Start High-Fidelity Polymerase (New England Biolabs) with the primers listed in Table 2 in accordance with the manufacturer's recommendations. Note that the number of PCR reactions is flexible and may change according to the amount of fragment required for Virus booting in any given application. For the present study we found that the yield produced by 8 PCR replicas is sufficient to produce the required fragment "building blocks".

**Table 2:** Primers used in the library synthesis and NGS analysis.

Fragment use	Size	Forward primer	Reverse primer
Backbone	1605	CCAATATCCCTATTTGATTATTTTATTGTTGGCGA	ACAGCGCACTTCTTTCGTTTTCAGATAT
Variant	271	CATATCTGAAAACGAAAGAAGTGCGCTGTAAGTATTACCAGCGCACTTCGGC	GCGAACCCCTGGAGGTGAGGTGTTCGTCCTTCCTCATTACCCTCCTCGCCAACAATAAAATAATCAAAT
NGS	230	GCTGTAAGTATTACCAGCGCACT	GGTGTTCGTCCTTCCTCATTAC

Amplification products were then confirmed for size on a 1% agarose gel, purified using MinElute PCR purification kit (Qiagen) and quantified (Nanodrop 1000, Thermo Scientific) prior to transfections.

### Cell transfection and subsequent serial passages

To "boot" the PCV2b synthetic genome library to life, PK15-C1 cells were transfected with an equimolar mix of PCV2b backbone and oligo pool amplicons. Briefly, PK15-C1 cells were grown in T25 flasks overnight at 37 °C to approximately 85% confluency. The next day, the cells were washed once with phosphate-buffered saline (PBS) and transfected with 6.3 µg of the relevant DNA mix using TransIT-X2 Dynamic Delivery System (Mirus) according to the manufacturer’s protocol (MIRUSBIO, USA). Additional cells that were transfected with an equimolar mix of PCV2b backbone fragment and a corresponding wildtype PCV2b amplicon, as well as mock cells transfected with only one of either fragment, served as positive and negative controls, respectively. The transfection mixtures were added to flasks of PK15-C1 cells containing 2.5 ml of fresh MEM medium for incubation of 5 h at 37 °C. Post incubation the cells were washed 3 times with PBS and overlaid with 5 mL of fresh MEM medium supplemented with 2% FBS. The virus was allowed to replicate in transfected cells for 3 days, and then harvested and stored at − 80 °C until subsequent passage.

For serial passages, the cells-and-medium-containing virus stock was first clarified through centrifugation (400 × g) for 10 min at 4 °C. Then, the cells’ supernatant was collected and used to re-infect fresh cells (grown in T25 flasks overnight at 37 °C to approximately 85% confluency). After an incubation period of 5 h at 37 °C under 5% CO_2_ the virus-containing supernatant used for infection was removed and cells were washed 3 times with PBS prior to being overlaid with 5 ml of fresh MEM medium supplemented with 2% inactive FBS. The cells and media were again harvested at 3 days post-infection and stored at − 80 °C for subsequent passages. This procedure was repeated for at least 6 generations and aliquots from each passage were stored at − 80◦C for later titration of the stock and NGS sequencing.

### Virus titration by infectious fluorescent focus assay (FFA)

To determine the infectious titer of virus stocks, PK15-C1 were seeded at 2 × 10^4^/well in 96-well optical-bottom plate (Nunc) and grown overnight at 37 °C to approximately 90% confluency.

The next day, wells populated with PK15-C1 were inoculated with virus stocks from 6 passaged generations serially diluted tenfold in MEM medium supplemented with 2% FBS. Dilutions were prepared in duplicate and inoculum was allowed to infect the cells for 4 h at 37 °C under 5% CO_2_. A negative control (non-inoculated cells) was included in all titrations. At 3 days post-infection the confluent monolayers of PK15-C1 cells were fixed with a solution containing 80% acetone and 20% methanol at 4 °C for 20 min. After carefully washing with PBS buffer, the infected cells and controls were incubated with pig anti-PCV2 polyclonal antiserum (VMRD, USA) diluted 1:2000 in PBS buffer supplemented with 1% BSA for 1 h at 37 °C. Cells were then washed 3 times with PBST (0.05% Tween-20 in PBS, pH 7.4), and incubated with fluorescein (FITC)-affiniPure secondary goat anti-swine IgG antibody (Jackson ImmunoResearch) for 45 min at 37 °C in the dark. Finally, cells were washed 3 more times with PBST prior to quantification of the numbers of infected cells (fluorescent foci) under a FITC-fitted fluorescent microscope at 20 × magnification.

### Next-generation sequencing (NGS)

Virus DNA was extracted from aliquoted culture supernatants taken at generations (passages) 4 and 6 using the QIAamp DNA Mini Kit (Qiagen). The DNA was then amplified by low cycle PCR (up to 23 cycles) using Q5 high-fidelity DNA polymerase (New England Biolabs) according to the manufacturer’s instructions. Overall, a total of 8 replica PCR reactions were carried out using the synthetic virus library DNA as a template. Primers used for amplification (Table 2) were pre-designed to flank a variable 230 base pairs genome fragment in synthetic PCV2b viruses created in PK15-C1 cells. Amplified PCR products were purified using the Zymoclean large fragment DNA recovery kit (Zymo) and run out on a 2% agarose gel to confirm the absence of non-specific amplifications. Confirmed amplicons (~ 230 base pairs) were prepared for sequencing using the Truseq DNA library preparation kit (Illumina). According to the TruSeq DNA sample preparation protocol, 100-ng purified amplicon pools were processed to generate blunt-ended, 5’-phosphorylated DNA, and an A-tailing reaction compatible with the adapter ligation strategy was performed. The ligation product was purified by sample purification beads. Post-library quantification and quality check (QC) were performed with BioAnalyzer DNA 1000 chip (Agilent) and the Qubit dsDNA High Sensitivity fluorometric assay (Invitrogen). The size distribution and quality of the library was verified using TapeStation 2200 System (Agilent Technologies, Santa Clara, CA). PhiX Control library (v3) (Illumina) was then combined with the amplicon library (expected at 20%) and subsequent paired-end Illumina sequencing was performed on a NextSeq 500 Illumina platform using the high output kit v2 kit with 300 cycles (150 base pairs, paired-end sequencing). Sample denaturation and loading was conducted following the manufacturer standard protocol.

In an attempt to maximize the sensitivity and effectiveness of the sequencing reaction as measured by sequence quality 4 protocols were used to prepare PCR samples for sequencing. First, the library DNA was amplified using two different sets of primers flanking the variable 230 base pair genome fragment. One set was homologous to the sequence flanks, while in the other set, a primer set added random heterogeneity spacer regions (0–4 nucleotides) upstream of binding. The resulting PCR products were then either extracted from gel (to eliminate unspecific amplicons) or purified as a whole using standard silica-based spin columns.

### NGS mapping

We sequenced the variable region with pair end sequencing; the read length of each side was 150 nucleotides. We trimmed adaptors from the reads using Cutadapt^69^ (version 1.12), and utilized Bowtie2^70^ (version 2.2.9) to map them to each of the possible sequences. We used Bowtie2 parameters '–gbar 150 –local -X 240 -I 200 '–mp 150, 100 –np 100 –rdg 500, 300 –rfg 500, 300 –score-min L,200,0 -a'. We then selected the targets with the paired-end overlap with the best combined alignment score among all concordant alignments (SAM tag YT:Z:CP) in which each mate is at least 100 nucleotides long and the combined length (including the overlap) is at least 200 nucleotides ^71^ to get the read-count for each variant.

The above procedure was used to calculate read counts on all samples. To get the final normalized values and filter biases, a normalization factor (*RC0_factor*) was calculated for each sample/lane separately and for each variant (vi):

$${\text{RC}}0\_{\text{factor}}_{{{\text{vi}}}} = {\text{RC}}0_{{{\text{WT}}}} /{\text{RC}}0_{{{\text{vi}}}}$$

where RC0_*vi*_ is the Read Count of *vi* at time 0, and RC0_WT_ is the RC of the wild type at time 0. For later time points (P2, P4, P6) the normalization was done by multiplying RC_vi_ with RC0_factor_vi_*.* Following that, values of the four protocols were averaged to get the final values.

### Genomic analyses

We downloaded PCV sequences from the National Center for Biotechnology Information—NCBI^72^ at 18-Feb-2018. Only variants with 1650–1850 nucleotides were included and that had information on both ORF1 and ORF2, see Supplementary Table S4), 2023 wildtype variants of PCV2 were analyzed. In this study, WT is referred to all of these genomes. ORF1 and ORF2 were analyzed separately. A multiple sequence alignment (MSA) of the amino acid (AA) level was performed on each ORF (ORF1 and ORF2) using the scoring matrix "BLOSUM90" and the MATLAB function "multialign"^73^. Based on the AA alignment, we retrieve the nucleotide alignment.

### Di-nucleotide sequence randomization

For each wildtype sequence and every ORF (ORF1 and ORF2) a di-nucleotide randomization was performed 100 times using different randomization seeds. The first step in this randomization is to find all “legal” swaps. This is not a sequence-dependent step. A “legal swap” is a swap that doesn’t change the AA sequence and that doesn’t change the nucleotide couple distribution of the sequence. There are 1584 legal swaps. For example, in the following sequence the bold nucleotides can be swapped if the reading frame is such that the 3 nucleotides with the underline are a codon so that A**C**T and A**T**T are both translated to the amino acid Threonine.

$$\underline{{{\text{ACT}}}} {\text{A}} \ldots \ldots \underline{{{\text{ATT}}}} {\text{A}}$$

The second step is finding legal swaps within the sequence from the general list of legal swaps considering the correct reading frame and dividing the swaps into groups so that every nucleotide in a position within the group can be swapped with a nucleotide in the other positions in the group. The nucleotides within each group were permutated once based on the randomization seed. Between each two permutations the grouped list was updated according to the previous permutation.

### Entropy

Each position in the MSA for each ORF was given an entropy score. The entropy was calculated according to the following equation^74^:

1 $$S = - \sum\nolimits_{i} {p_{i} \cdot \log_{2} (p_{i} )}$$

where S is entropy and p is the probability of finding the character i in the sequence. The entropy score was normalized by dividing the value by 2 (which is the entropy score of a perfectly random nucleotide sequence).

### Folding energy

An average absolute folding energy score was calculated for each position in each of the two main ORFs (ORF1 and ORF2). For each wildtype sequence, in each position, a sequence of 31 nucleotides was taken around the position (with the nucleotide at the center of the sequence). The 31-nucleotide sequence was given as an input to the MATLAB function “rnafold”^75^.

An average value for each position was calculated using all the wildtype sequences in the MSA. Folding energy results of other 'window sizes' (25 and 37) can be seen in Supplementary Fig. S3. The absolute folding energy of the wildtype sequence KJ128273 (both ORF1 and ORF2) was compared to an average of 1000 random sequences based on KJ128273 with a 31 nucleotide window as shown in Supplementary Fig. S2.

### Codon adaptation Index—CAI

The Codon Adaptation Index (CAI) measures the degree with which genes use preferred codons^76^. The CAI value for a gene is calculated as the geometric mean of w_i_ values (weights) for all the codons used in that gene.

2 $${\text{CAI = }}\mathop {\left( {\prod\limits_{{\text{i = 1}}}^{{\text{L}}} {{\text{w}}_{{\text{i}}} } } \right)}\nolimits^{{\frac{{1}}{{\text{L}}}}}$$

where L = number of codons in the ORF. The weights were calculated according to codon usage data for domestic pigs (see Supplementary Table S1)^77,78^.

### The effective number of codons—ENC

The Effective Number of Codons measures the degree in which genes use more specific codons as opposed to using all codons uniformly^79^. ENC was calculated in the following manner:

3 $$F_{AA} = p_{1}^{2} + p_{2}^{2} + ... + p_{k}^{2}$$

where $F_{AA}$ is the probability that 2 random codons that encode the same amino acid are identical and $p_{i}$ is the actual frequency with which the codon i encodes the amino acid in the sequence.

4 $$F_{2} = \frac{{F_{Asn} + F_{Asp} + F_{Cys} + F_{G\ln } + F_{Glu} + F_{His} + F_{Lys} + F_{Phe} F_{Tyr} }}{9}$$

5 $$F_{3} = F_{Ile}$$

6 $$F_{4} = \frac{{F_{Ala} + F_{Gly} + F_{Thr} + F_{\Pr o} + F_{Val} }}{5}$$

7 $$F_{6} = \frac{{F_{Arg} + F_{Leu} + F_{Ser} }}{3}$$

8 $$ENC = 2 + \frac{9}{{F_{2} }} + \frac{1}{{F_{3} }} + \frac{5}{{F_{4} }} + \frac{3}{{F_{6} }}$$

### Differences in average fitness with/without mutation histograms separated by entropy ranking of position

This was done by: (A) getting an entropy score for each position from the wildtype data taken from NCBI; (B) in each position, dividing the sequences into 2 groups: with/without a mutation in this position (compared to the wildtype); (C) subtracting the average fitness of the mutation group from that of the group without mutations to calculate a vector of fitness differences by location,and linking it to the entropy by location vector to rank them by entropy; and (D) splitting the results into 2 groups: top 10% entropy values and bottom 10% entropy values, and displaying the results as 2 different histograms.

### Fitness predictor

Prediction of fitness required the creation of various features for each position:

**Mutation in position “mut_pos_#”:** an indication for every sequence and each position whether there is a mutation in the position compared to the wildtype sequence KJ128273.

**mRNA folding energy difference “RNA_FE_diff_#”:**

1. For each position and sequence, a short sequence of 31 nucleotides was taken around the position.
2. The above short sequence was given as an input to the MATLAB function “rnafold”^75^.
3. The “rnafold” output of the above sequence was assigned as the average “mRNA FE” of the center position of the short sequence.
4. For each sequence, the difference between the mRNA FE value in each position and the value of the same position in the wildtype, was calculated.

**DNA folding energy difference “DNA_FE_diff_#”:**

1. For each position and sequence, a short sequence of 31 nucleotides was taken around the position.
2. The above short sequence was given as an input to the MATLAB function “oligoprop”^80^.
3. The function “oligoprop” returns the “Gibbs Free Energy” of the above short sequence according to 4 different models^81–84^. The average value of the 4 models was assigned as an average “DNA FE” of the center position in the short sequence.
4. For each sequence, the difference between the DNA FE value in each position and the value of the same position in the wildtype, was calculated.

**mRNA topological distance “mRNA_topo_dist_#”:**

1. In all of the sequences, for each position, short sequences of 31 nucleotides were taken around the position. The first sequence is the wildtype sequence.
2. The above short sequences were given as an input to the function “RNApdist” of the Vienna software package^85^, with the flag “-Xf”, which calculates distances between thermodynamic RNA secondary structures ensembles. “ − Xf” compare each structure to the first one.
3. The output of the function “RNApdist” was assigned as an average “mRNA topological distance” in the center position for every short sequence.

**DNA topological distance:**

1. Each sequence was given as an input to the function “CurvedDNA” from the python package “dnacurve”, with the “trifonov” model as a parameter^86–92^.
2. The outputs of the function are 18 structural parameters for each position in the sequence: Curvature, Bend angel, Curvature angel, Helix axis [X], Helix axis [Y], Helix axis [Z], Phosphate 1 [X], Phosphate 1 [Y], Phosphate 1 [Z], Phosphate 2 [X], Phosphate 2 [Y], Phosphate 2 [Z], Basepair normal [X], Basepair normal [Y], Basepair normal [Z], Smoothed normal [X], Smoothed normal [Y], Smoothed normal [Z].
3. For each sequence, the difference between each structural parameter value in each position and the value of the same position in the wildtype, was calculated.

### Fitness value

The fitness value of each sequence was set as the average titer value for P2, P4 and P6.

### Running the predictor

Predictions were run twenty times, randomly dividing the dataset into three groups: training (300), test (100) and validation (100) using the following protocol:

1. Finding the 1st vector in the model—in the test set, finding the vector with the highest Spearman correlation with the fitness vector. Taking the vector into the model.
2. In each iteration, adding one vector to the model to maximally increase the Spearman’s correlation between the model and the fitness vector of the test group. The model is built using Linear Regression, the coefficients are calculated using the MATLAB function “regress”^93^ on the training set.
3. The addition of vectors to the model stops when the correlation stops increasing.
4. The finished model includes the selected vectors (features) and their coefficients. The model was run once on the validation set.

## Supplementary Information

Supplementary Information.
