# Supplementary material for: Computational based design and tracking of synthetic variants of Porcine circovirus reveal relations between silent genomic information and viral fitness
Source: Sci Rep. 2021 May 19;11:10620. doi: 10.1038/s41598-021-89918-6 (PMC8134455; doi:10.1038/s41598-021-89918-6)
Supplement: Supplementary file 1 — Supplementary Information. [file 41598_2021_89918_MOESM1_ESM.pdf]

Computational based design and tracking of synthetic variants of Porcine circovirus reveal relations between silent genomic information and viral fitness

Lia Baron, Shimshi Atar*, Hadas Zur*, Modi Roopin*, Eli Goz, Tamir Tuller*

Supplementary

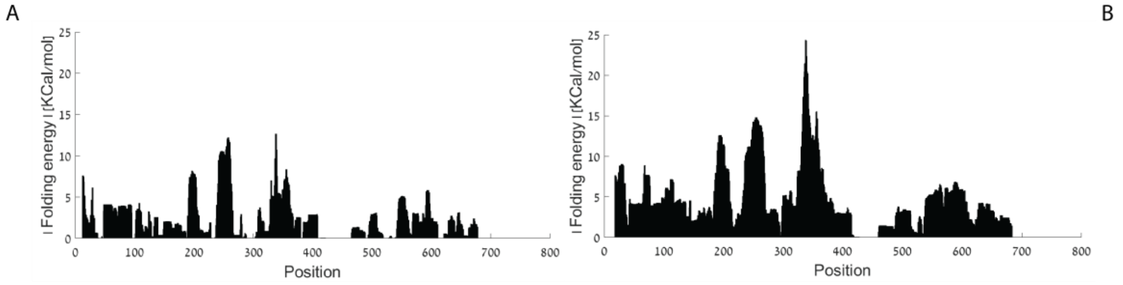

Figure S1. Absolute folding energy per AA aligned position of PCV2 ORF2 (KJ128273): (**A**) window size = 25 (**B**) window size = 37

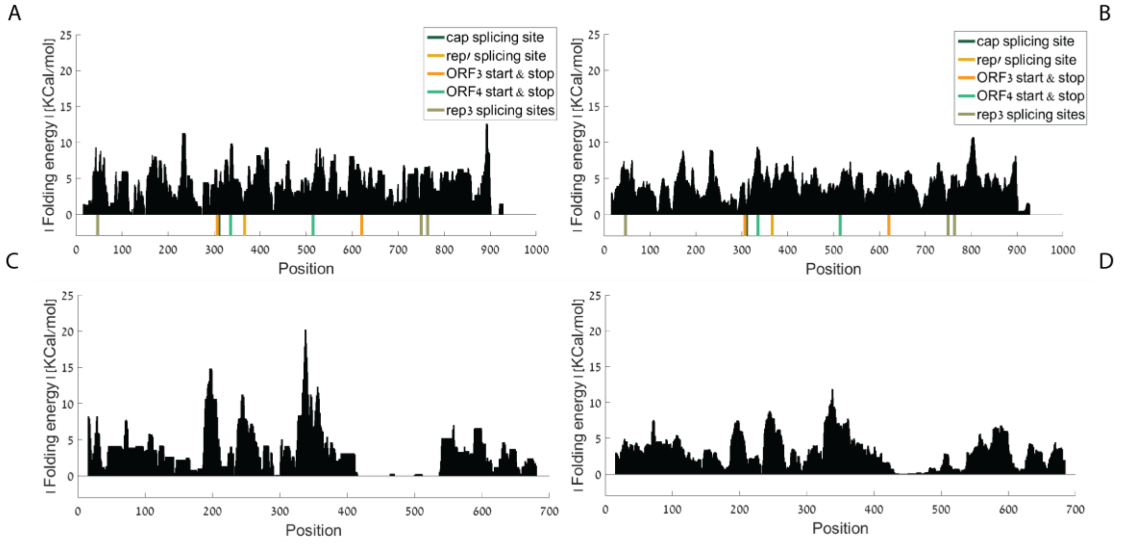

Figure S2. Absolute folding energy profile – real strain compared to average of a 1,000 random strains, window size = 31: (**A**) KJ128273 ORF1; (**B**) average on a 1,000 random ORF1; (**C**) KJ128273 ORF2 (**D**) Average on a 1,000 random ORF2

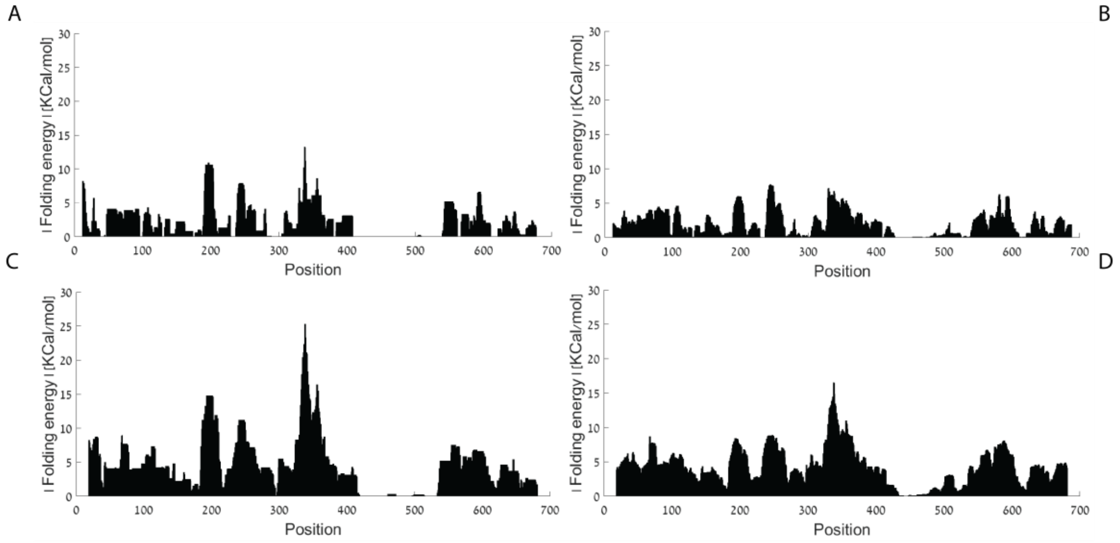

Figure S3. Absolute folding energy profile – PCV2 ORF2 real strain compared to average of a 1,000 random strains: (**A**) KJ128273, window size = 25 (**B**) Average on a 1,000 random, window size = 25 (**C**) KJ128273, window size = 37 (**D**) Average on a 1,000 random, window size = 37

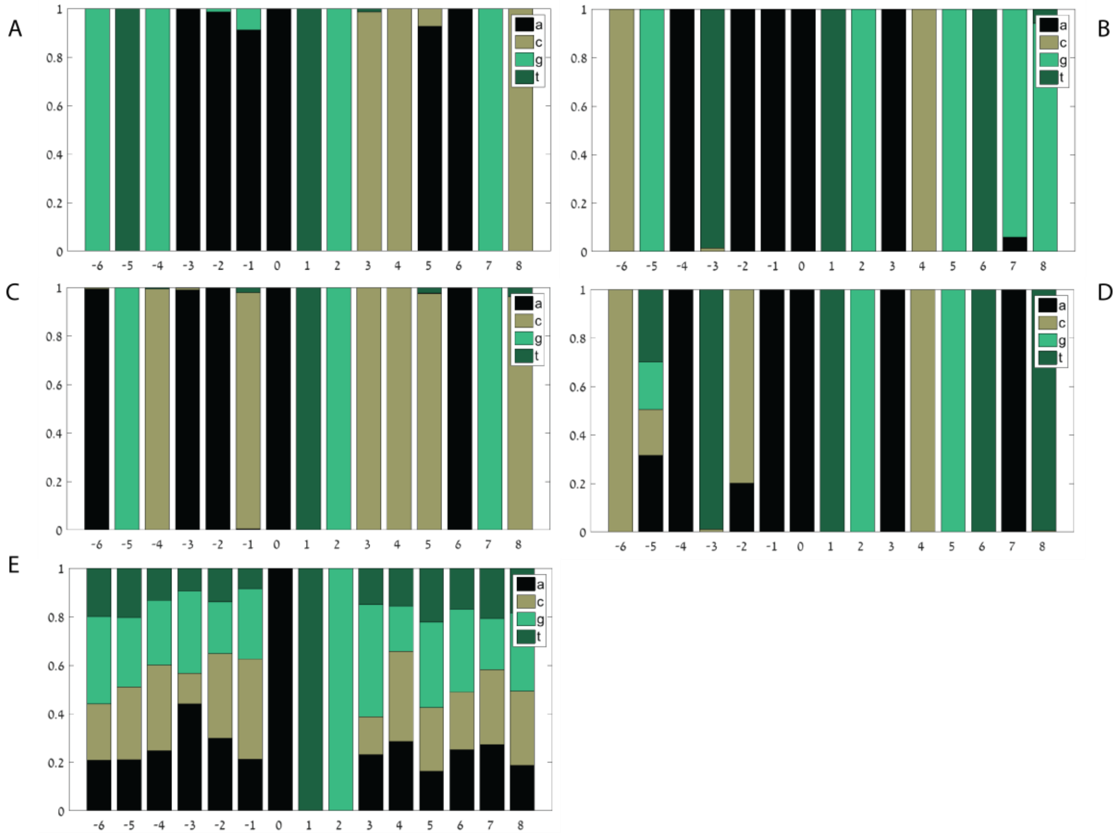

Figure S4. ATG context of: (**A**) PCV1 ORF1 (**B**) PCV1 ORF2 (**C**) PCV2 ORF1 (**D**) PCV2 ORF2 (**E**) Sus Scrofa Domesticus genes. The consensus of each image is: (**A**) GTGAAA**ATG**CCAAGC (**B**) CGATAA**ATG**ACGTGG (**C**) AGCAAC**ATG**CCCAGC (**D**) CAATCA**ATG**ACGTAT; (**E**) GCCACC**ATG**GCGGCG. PCV2 ORF2 is less conserved compared to the others.

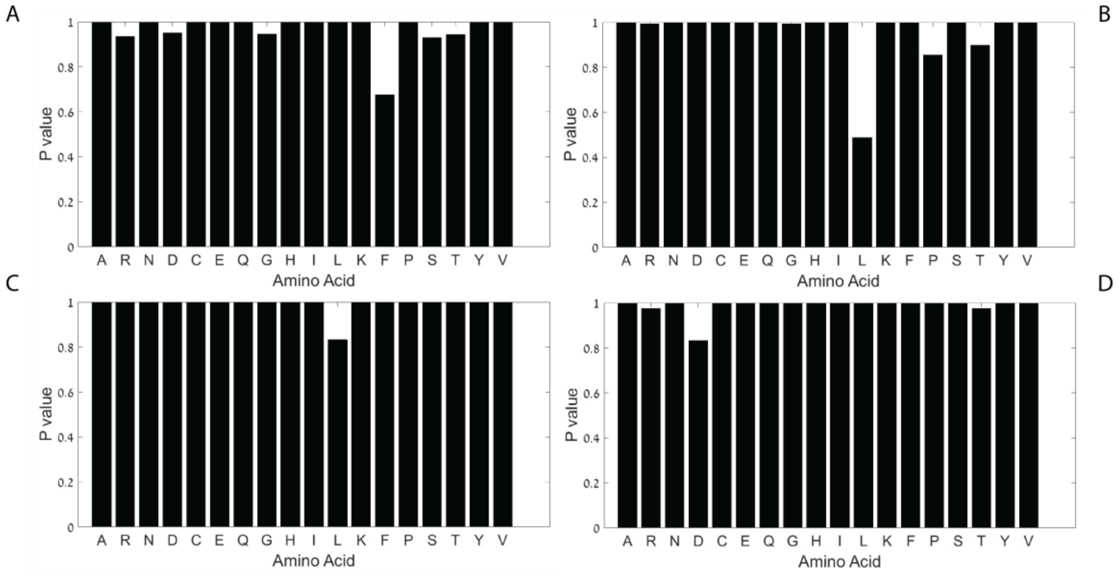

Figure S5. Empirical p values for codon entropy of each amino acid in the strain KJ128273 (**A**) ORF1, high entropy compared to random (**B**) ORF1, low entropy compared to random (**C**) ORF2, high entropy compared to random (**D**) low entropy compared to random

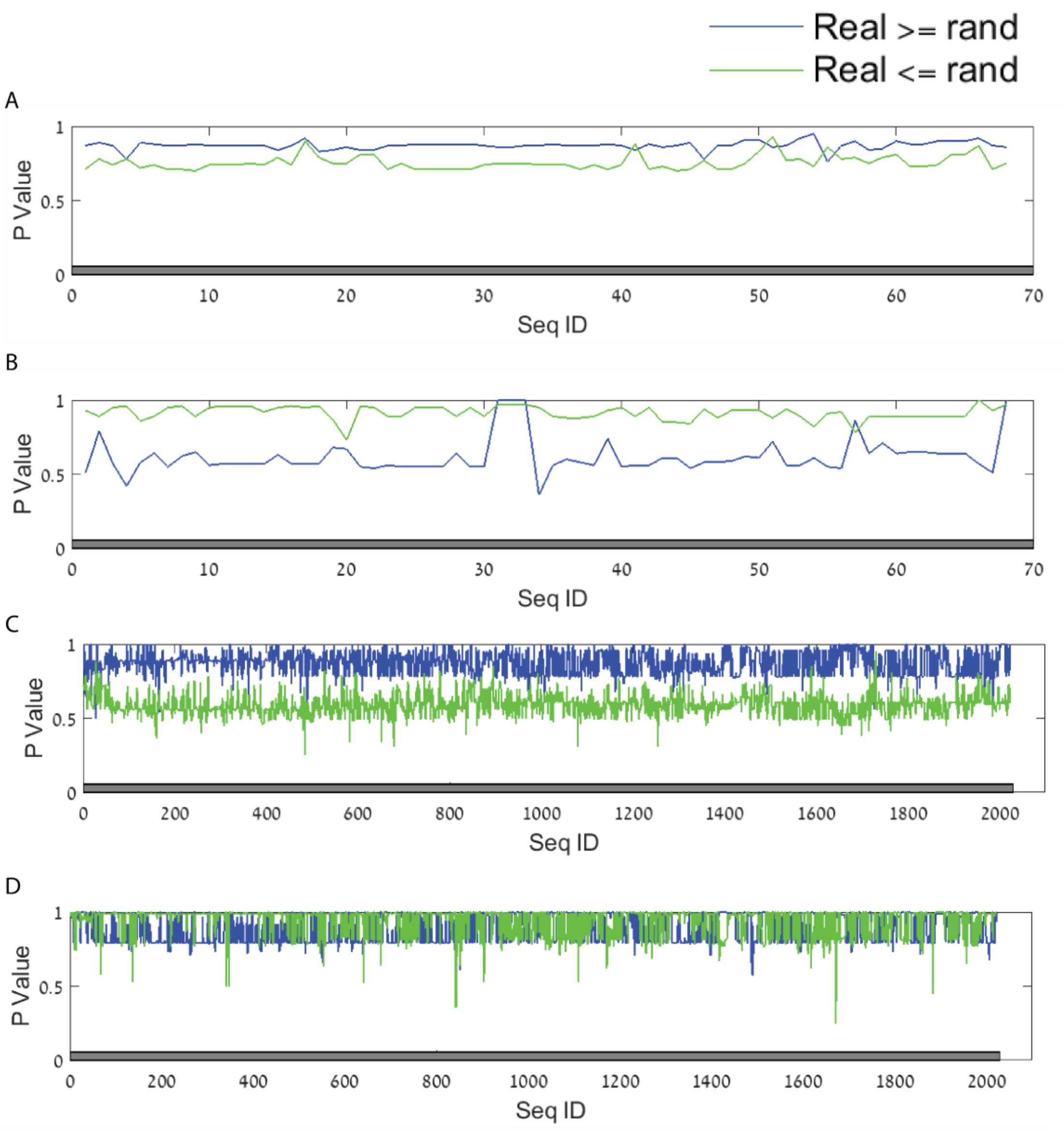

Figure S6. P value vs. sequence ID of CAI compared to randomized sequences. In blue - real higher than or equal to rand, in green - real lower than or equal to rand, in dark gray – the statistically significant region (P value <= 0.05). (**A**) PCV1 ORF1 (**B**) PCV1 ORF2 (**C**) PCV2 ORF1 (**D**) PCV2 ORF2

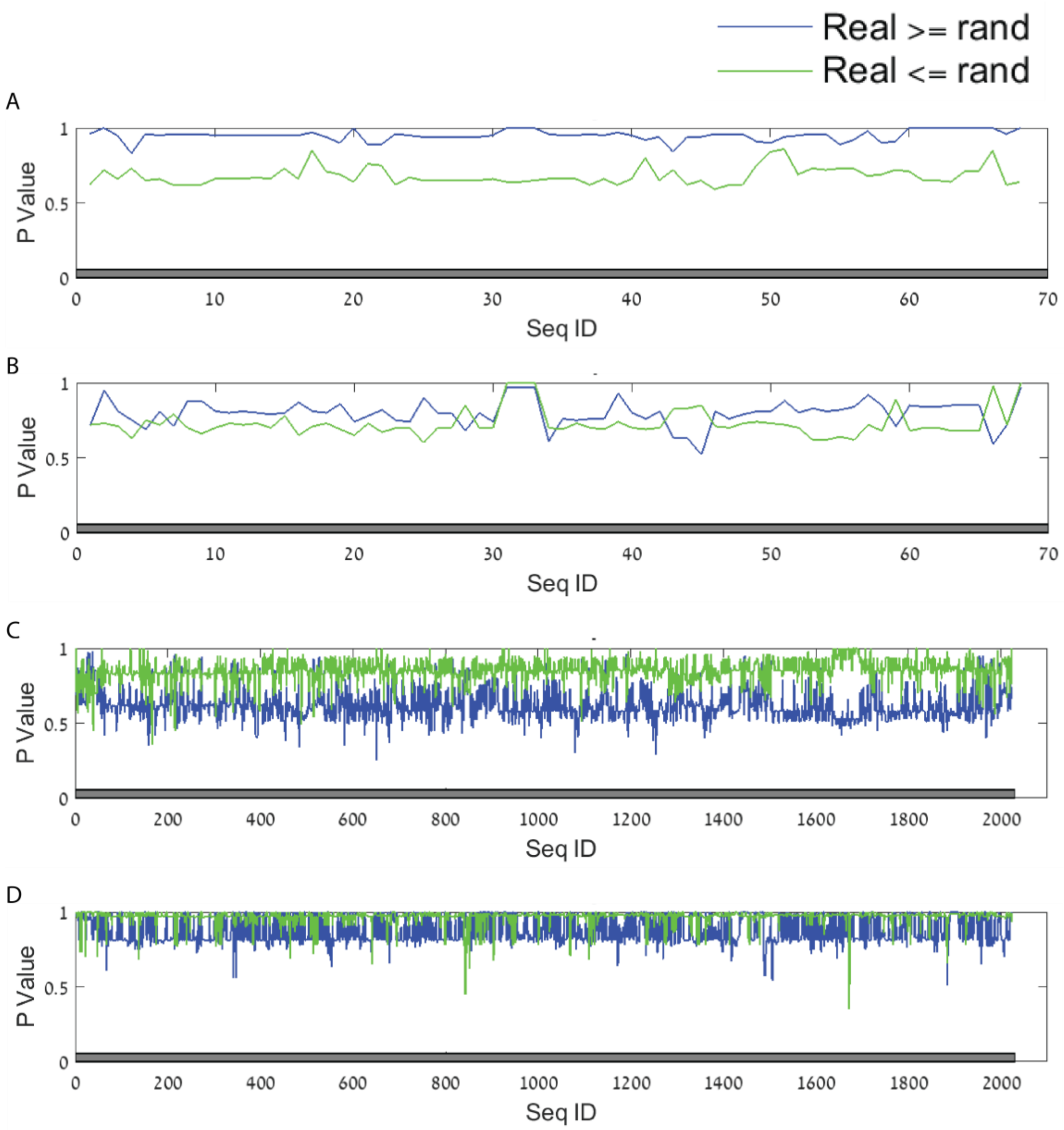

Figure S7. P value vs. sequence ID of ENC compared to randomized sequences. In blue - real higher than or equal to rand, in green - real lower than or equal to rand, in dark gray – the statistically significant region (P value <= 0.05). (**A**) PCV1 ORF1 (**B**) PCV1 ORF2 (**C**) PCV2 ORF1 (**D**) PCV2 ORF2

Amino acid	Codon	Weight
Phenylalanine	TTT	0.006
Phenylalanine	TTC	0.0143
Leucine	TTA	0.0030
Leucine	TTG	0.0049
Leucine	CTT	0.0058
Leucine	CTC	0.0139
Leucine	CTA	0.0028
Leucine	CTG	0.0289
Isoleucine	ATT	0.0131
Isoleucine	ATC	0.0157

Isoleucine	ATA	0.0036
Methionine	ATG	0.0106
Valine	GTT	0.0062
Valine	GTC	0.0262
Valine	GTA	0.0031
Valine	GTG	0.0368
Serine	TCT	0.0301
Serine	TCC	0.0273
Serine	TCA	0.0671
Serine	TCG	0.0151
Proline	CCT	0.0123
Proline	CCC	0.0233
Proline	CCA	0.0133
Proline	CCG	0.0096
Threonine	ACT	0.0191
Threonine	ACC	0.0296
Threonine	ACA	0.0398
Threonine	ACG	0.0100
Alanine	GCT	0.0203
Alanine	GCC	0.0298
Alanine	GCA	0.0558
Alanine	GCG	0.0105
Tyrosine	TAT	0.0046
Tyrosine	TAC	0.0088
Stop	TAA	0.0003
Stop	TAG	0.0002
Histidine	CAT	0.0035
Histidine	CAC	0.0081
Glutamine	CAA	0.0047
Glutamine	CAG	0.0276
Asparagine	AAT	0.0080
Asparagine	AAC	0.0116
Lysine	AAA	0.0098
Lysine	AAG	0.0117
Aspartic Acid	GAT	0.0091
Aspartic Acid	GAC	0.0134
Glutamic Acid	GAA	0.0234
Glutamic Acid	GAG	0.0144
Cysteine	TGT	0.0068
Cysteine	TGC	0.0100
Stop	TGA	0.0005
Tryptophan	TGG	0.0080
Arginine	CGT	0.0024
Arginine	CGC	0.0064
Arginine	CGA	0.0016
Arginine	CGG	0.0045
Serine	AGT	0.0070
Serine	AGC	0.0180
Arginine	AGA	0.0160
Arginine	AGG	0.0042

Glycine	GGT	0.0113
Glycine	GGC	0.0312
Glycine	GGA	0.0659
Glycine	GGG	0.0394

Table S1. Codon weights according to the distribution in Sus scrofa domestica [55,56]

	PCV1		PCV2	
	**ORF1**	**ORF2**	**ORF1**	**ORF2**
**CAI**	0.5257	0.5681	0.5434	0.5688
**CAI std**	0.0035	0.0061	0.0068	0.0075
**ENC**	49.0714	46.1022	48.5462	41.5509
**ENC std**	0.2400	1.7664	0.9156	0.6729

Table S2. CAI & ENC results – mean and standard deviation for the NCBI data (PCV1 and PCV2 – ORF1 and ORF2)

Full wildtype sequence (NCBI accession number KJ128273)	CTTTTTTATCACTTCGTAATGGTTTTTATTATTCATTAAGGGTTAAGTGGGG<br>GGTCTTTAAGATTAAATTCTCTGAATTGTACATACATGGTTACACGGATAT<br>TGTATTCCTGGTCGTATATACTGTTTTCGAACGCAGTGCCGAGGCCTACGT<br>GGTCTACATTTCCAGTAGTTTGTAGTCTCAGCCACAGCTGGTTTCTTTTGTT<br>GTTTGGTTGGAAGTAATCAATAGTGGAATCTAGGACAGGTTTGGGGGTAA<br>AGTAGCGGGAGTGGTAGGAGAAGGGCTGGGTTATGGTATGGCGGGAGG<br>AGTAGTTTACATAGGGGTCATAGGTGAGGGCTGTGGCCTTTGTTACAAAG<br>TTATCATCTAGAATAACAGCACTGGAGCCCACTCCCCTGTCACCCTGGGTG<br>ATCGGGGAGCAGGGCCAGAATTCAACCTTAACCTTTCTTATTCTGTAGTAT<br>TCAAAGGGCACAGAGCGGGGGTTTGAGCCTCCTCCTGGGGGAAGAAAGT<br>CATTAATATTGAATCTCATCATGTCCACCGCCCAGGAGGGCGTTCTGACTG<br>TGGTTCGCTTGATAGTATATCCGAAGGTGCGGGAGAGGCGGGTGTTGAA<br>GATGCCATTTTTCCTTCTCCAGCGGTAACGGTGGCGGGGGTGGACGAGCC<br>AGGGGCGGCGGCGGAGGATCTGGCCAAGATGGCTGCGGGGGCGGTGTC<br>TTCTTCTCCGGTAACGCCTCCTTGGATACGTCAT**ATCTGAAAACGAAAGA**<br>**AGTGCGCTGTAAGTATTACCAGCGCACTTCGGCAGCGGCAGCACCTCGG**<br>**CAGCACCTCAGCAGCAACATGCCCAGCAAGAAGAATGGAAGAAGCGGA**<br>**CCCCAACCCCATAAAAGGTGGGTGTTCACTCTGAATAATCCTTCCGAAGA**<br>**CGAGCGCAAGAAAATACGGGATCTTCCAATATCCCTATTTGATTATT**TTA<br>TTGTTGGCGAGGAGGGTAATGAGGAAGGACGAACACCTCACCTCCAGGG<br>GTTCGCTAATTTTGTGAAGAAGCAGACTTTTAATAAAGTGAAGTGGTATTT<br>GGGTGCCCGCTGCCACATCGAGAAAGCGAAAGGAACAGATCAGCAGAAT<br>AAAGAATACTGCAGTAAAGAAGGCAACTTACTGATTGAGTGTGGAGCTCC<br>GAGATCTCAGGGACAACGGAGTGACCTGTCTACTGCTGTGAGTACCTTGT<br>TGGAGAGCGGGAGTCTGGTGACCGTTGCAGAGCAGCACCCTGTAACGTTT<br>GTCAGAAATTTCCGCGGGCTGGCTGAACTTTTGAAAGTGAGCGGGAAAAT<br>GCAGAAGCGTGATTGGAAGACTAATGTACACGTCATTGTGGGGCCACCTG<br>GGTGTGGTAAAAGCAAATGGGCTGCTAATTTTGCAGACCCGGAAACCACA<br>TACTGGAAACCACCTAGAAACAAGTGGTGGGATGGTTACCATGGTGAAG<br>AAGTGGTTGTTATTGATGACTTTTATGGCTGGCTGCCCTGGGATGATCTAC<br>TGAGACTGTGTGATCGATATCCATTGACTGTAGAGACTAAAGGTGGAACT<br>GTACCTTTTTTGGCCCGCAGTATTCTGATTACCAGCAATCAGACCCCGTTG<br>GAATGGTACTCCTCAACTGCTGTCCCAGCTGTAGAAGCTCTTTATCGGAGG<br>ATTACTTCCTTGGTATTTTGGAAGAATGCTACAGAACAATCCACGGAGGA<br>AGGGGGCCAATTCGTCACCCTTTCCCCCCCATGCCCTGAATTTCCCTATGA<br>AATAAATTACTGAGT
Sequence_1_WT	**ATCTGAAAACGAAAGAAGTGCGCTGTAAGTATTACCAGCGCACTTCG**<br>**GCAGCGGCAGCACCTCGGCAGCACCTCAGCAGCAACATGCCCAGCAAGA**<br>**AGAATGGAAGAAGCGGACCCCAACCCCATAAAAGGTGGGTGTTCACTCT**<br>**GAATAATCCTTCCGAAGACGAGCGCAAGAAAATACGGGATCTTCCAATA**<br>**TCCCTATTTGATTATT**
Sequence_2	**ATCTGAAAACGAAAGGGGGGCGCCGGTAGTATTACCGGCGCCCCCCG**<br>**GCAGCGGCAGCACCTCGGCAGCACCTCAGCAGCAACATGCCCAGCAAGA**<br>**AGAATGGAAGAAGCGGACCCCAACCCCATAAAAGGTGGGTGTTCACTCT**<br>**GAATAATCCTTCCGAAGACGAGCGCAAGAAAATACGGGATCTTCCAATA**

	**TCCCTATTTGATTATT**
Sequence_3	**ATCTGAAAACGAAAGAGGGCGCGGCTGTAGTGTTACCCGCGCCCTCG GCAGCGGCAGCACCTCGGCAGCACCTCAGCAGCAACATGCCCAGCAAGA AGAATGGAAGAAGCGGACCCCAACCCCATAAAAGGTGGGTGTTCACTCT GAATAATCCTTCCGAAGACGAGCGCAAGAAAATACGGGATCTTCCAATA TCCCTATTTGATTATT**
Sequence_4	**ATCTGAAAACGAAAGCGTGCGCGGCTGTAGTATTACCCGCGCACGCG GCAGCGGCAGCACCTCGGCAGCACCTCAGCAGCAACATGCCCAGCAAGA AGAATGGAAGAAGCGGACCCCAACCCCATAAAAGGTGGGTGTTCACTCT GAATAATCCTTCCGAAGACGAGCGCAAGAAAATACGGGATCTTCCAATA TCCCTATTTGATTATT**
Sequence_5	**ATCTGAAAACGAAAACGCGCGCCGCTGTAGTATTACCGGCGCGCGTG GCAGCGGCAGCACCTCGGCAGCACCTCAGCAGCAACATGCCCAGCAAGA AGAATGGAAGAAGCGGACCCCAACCCCATAAAAGGTGGGTGTTCACTCT GAATAATCCTTCCGAAGACGAGCGCAAGAAAATACGGGATCTTCCAATA TCCCTATTTGATTATT**
Sequence_6	**ATCTGAAAACGAAAGCAGGGCGCGGGTAGTATTACCCGCGTCCTGCG GCAGCGGCAGCACCTCGGCAGCACCTCAGCAGCAACATGCCCAGCAAGA AGAATGGAAGAAGCGGACCCCAACCCCATAAAAGGTGGGTGTTCACTCT GAATAATCCTTCCGAAGACGAGCGCAAGAAAATACGGGATCTTCCAATA TCCCTATTTGATTATT**
Sequence_7	**ATCTGAAAACGAAAGGGGGGCGCCGTAGGTATTACCGGCGCCCCCCG GCAGCGGCAGCACCTCGGCAGCACCTCAGCAGCAACATGCCCAGCAAGA AGAATGGAAGAAGCGGACCCCAACCCCATAAAAGGTGGGTGTTCACTCT GAATAATCCTTCCGAAGACGAGCGCAAGAAAATACGGGATCTTCCAATA TCCCTATTTGATTATT**
Sequence_8	**ATCTGAAAACGAAAGCAGGGCGCGGTAAGTATTACCCGCGCCCTGCG GCAGCGGCAGCACCTCGGCAGCACCTCAGCAGCAACATGCCCAGCAAGA AGAATGGAAGAAGCGGACCCCAACCCCATAAAAGGTGGGTGTTCACTCT GAATAATCCTTCCGAAGACGAGCGCAAGAAAATACGGGATCTTCCAATA TCCCTATTTGATTATT**
Sequence_9	**ATCTGAAAACGAAAGGGGGGCGCGGTAAGTATTACCCGCGCCCCCTG GCAGCGGCAGCACCTCGGCAGCACCTCAGCAGCAACATGCCCAGCAAGA AGAATGGAAGAAGCGGACCCCAACCCCATAAAAGGTGGGTGTTCACTCT GAATAATCCTTCCGAAGACGAGCGCAAGAAAATACGGGATCTTCCAATA TCCCTATTTGATTATT**
Sequence_10	**ATCTGAAAACGAAAGACGCGCGCGGTAAGTTTTACCCGCGCGCGTCG GCAGCGGCAGCACCTCGGCAGCACCTCAGCAGCAACATGCCCAGCAAGA AGAATGGAAGAAGCGGACCCCAACCCCATAAAAGGTGGGTGTTCACTCT GAATAATCCTTCCGAAGACGAGCGCAAGAAAATACGGGATCTTCCAATA TCCCTATTTGATTATT**
Sequence_11	**ATCTGAAAACGAAAGCGGCGCGCGGTATGTATTACCCGCGCGCCGCG**

	**GCAGCGGCAGCACCTCGGCAGCACCTCAGCAGCAACATGCCCAGCAAGAAGAATGGAAGAAGCGGACCCCAACCCCATAAAAGGTGGGTGTTCACTCTGAATAATCCTTCCGAAGACGAGCGCAAGAAAATACGGGATCTTCCAATATCCCTATTTGATTATT**
Sequence_12	**ATCTGAAAACGAAAAGGTGCGCGGCTGTAGTATTACCCGCGCGCCTGGCAGCGGCAGCACCTCGGCAGCACCTCAGCAGCAACATGCCCAGCAAGAAGAATGGAAGAAGCGGACCCCAACCCCATAAAAGGTGGGTGTTCACTCTGAATAATCCTTCCGAAGACGAGCGCAAGAAAATACGGGATCTTCCAATATCCCTATTTGATTATT**
Sequence_13	**ATCTGAAAACGAAAGGCGCGCGCGGTAAGTATTACCCGCGCGCGCTGGCAGCGGCAGCACCTCGGCAGCACCTCAGCAGCAACATGCCCAGCAAGAAGAATGGAAGAAGCGGACCCCAACCCCATAAAAGGTGGGTGTTCACTCTGAATAATCCTTCCGAAGACGAGCGCAAGAAAATACGGGATCTTCCAATATCCCTATTTGATTATT**
Sequence_14	**ATCTGAAAACGAAAGCAGGGCGCGGTATGTATTACCCGCGCCCTGCGGCAGCGGCAGCACCTCGGCAGCACCTCAGCAGCAACATGCCCAGCAAGAAGAATGGAAGAAGCGGACCCCAACCCCATAAAAGGTGGGTGTTCACTCTGAATAATCCTTCCGAAGACGAGCGCAAGAAAATACGGGATCTTCCAATATCCCTATTTGATTATT**
Sequence_15	**ATCTGAAAACGAAAGCAGTGCGCGGGTAGTGTTACCCGCGCACTGCGGCAGCGGCAGCACCTCGGCAGCACCTCAGCAGCAACATGCCCAGCAAGAAGAATGGAAGAAGCGGACCCCAACCCCATAAAAGGTGGGTGTTCACTCTGAATAATCCTTCCGAAGACGAGCGCAAGAAAATACGGGATCTTCCAATATCCCTATTTGATTATT**
Sequence_16	**ATCTGAAAACGAAAACGCGCGCGGCTGTAGTATTACCCGCGCGCGTGGCAGCGGCAGCACCTCGGCAGCACCTCAGCAGCAACATGCCCAGCAAGAAGAATGGAAGAAGCGGACCCCAACCCCATAAAAGGTGGGTGTTCACTCTGAATAATCCTTCCGAAGACGAGCGCAAGAAAATACGGGATCTTCCAATATCCCTATTTGATTATT**
Sequence_17	**ATCTGAAAACGAAACGGCGCGCGGCTGTAGTATTACCCGCGCGCCGGGCAGCGGCAGCACCTCGGCAGCACCTCAGCAGCAACATGCCCAGCAAGAAGAATGGAAGAAGCGGACCCCAACCCCATAAAAGGTGGGTGTTCACTCTGAATAATCCTTCCGAAGACGAGCGCAAGAAAATACGGGATCTTCCAATATCCCTATTTGATTATT**
Sequence_18	**ATCTGAAAACGAAAGACGTGTGCGGTAAGTATTACCCGCGCACGTCGGCAGCGGCAGCACCTCGGCAGCACCTCAGCAGCAACATGCCCAGCAAGAAGAATGGAAGAAGCGGACCCCAACCCCATAAAAGGTGGGTGTTCACTCTGAATAATCCTTCCGAAGACGAGCGCAAGAAAATACGGGATCTTCCAATATCCCTATTTGATTATT**
Sequence_19	**ATCTGAAAACGAAAGCAGTGCGCGGGTAGTATTACCCGCGCGCTGCGGCAGCGGCAGCACCTCGGCAGCACCTCAGCAGCAACATGCCCAGCAAGAAGAATGGAAGAAGCGGACCCCAACCCCATAAAAGGTGGGTGTTCACTCTGAATAATCCTTCCGAAGACGAGCGCAAGAAAATACGGGATCTTCCAATA**

	**TCCCTATTTGATTATT**
Sequence_20	**ATCTGAAAACGAAAGGGGTGCGCGGGTAGTATTACCCGCGCGCCCCGGCAGCGGCAGCACCTCGGCAGCACCTCAGCAGCAACATGCCCAGCAAGAAGAATGGAAGAAGCGGACCCCAACCCCATAAAAGGTGGGTGTTCACTCTGAATAATCCTTCCGAAGACGAGCGCAAGAAAATACGGGATCTTCCAATATCCCTATTTGATTATT**
Sequence_21	**ATCTGAAAACGAAAGGGGGGCGCCGGTAGTGTTACCGGCGCCCCCCGGCAGCGGCAGCACCTCGGCAGCACCTCAGCAGCAACATGCCCAGCAAGAAGAATGGAAGAAGCGGACCCCAACCCCATAAAAGGTGGGTGTTCACTCTGAATAATCCTTCCGAAGACGAGCGCAAGAAAATACGGGATCTTCCAATATCCCTATTTGATTATT**
Sequence_22	**ATCTGAAAACGAAAAAGGGCGCGGCTGTAGTATTACCCGCGCCCTTGGCAGCGGCAGCACCTCGGCAGCACCTCAGCAGCAACATGCCCAGCAAGAAGAATGGAAGAAGCGGACCCCAACCCCATAAAAGGTGGGTGTTCACTCTGAATAATCCTTCCGAAGACGAGCGCAAGAAAATACGGGATCTTCCAATATCCCTATTTGATTATT**
Sequence_23	**ATCTGAAAACGAAAGGGGGGCGCGGTAAGTATTACCCGCGCCTCCCGGCAGCGGCAGCACCTCGGCAGCACCTCAGCAGCAACATGCCCAGCAAGAAGAATGGAAGAAGCGGACCCCAACCCCATAAAAGGTGGGTGTTCACTCTGAATAATCCTTCCGAAGACGAGCGCAAGAAAATACGGGATCTTCCAATATCCCTATTTGATTATT**
Sequence_24	**ATCTGAAAACGAAAGAAATGCGCTTGTAGTATTACAAGCGCATTTCGGCAGCGGCAGCACCTCGGCAGCACCTCAGCAGCAACATGCCCAGCAAGAAGAATGGAAGAAGCGGACCCCAACCCCATAAAAGGTGGGTGTTCACTCTGAATAATCCTTCCGAAGACGAGCGCAAGAAAATACGGGATCTTCCAATATCCCTATTTGATTATT**
Sequence_25	**ATCTGAAAACGAAAAAGTGATATTCTGTAGTGTTACAATATCACTTGGCAGCGGCAGCACCTCGGCAGCACCTCAGCAGCAACATGCCCAGCAAGAAGAATGGAAGAAGCGGACCCCAACCCCATAAAAGGTGGGTGTTCACTCTGAATAATCCTTCCGAAGACGAGCGCAAGAAAATACGGGATCTTCCAATATCCCTATTTGATTATT**
Sequence_26	**ATCTGAAAACGAAAAAAATTCGATACTGTAGTATTACTATCGAATTTGGCAGCGGCAGCACCTCGGCAGCACCTCAGCAGCAACATGCCCAGCAAGAAGAATGGAAGAAGCGGACCCCAACCCCATAAAAGGTGGGTGTTCACTCTGAATAATCCTTCCGAAGACGAGCGCAAGAAAATACGGGATCTTCCAATATCCCTATTTGATTATT**
Sequence_27	**ATCTGAAAACGAAAAAATTCTATTCTGTAGTATTACAATAGAATTTGGCAGCGGCAGCACCTCGGCAGCACCTCAGCAGCAACATGCCCAGCAAGAAGAATGGAAGAAGCGGACCCCAACCCCATAAAAGGTGGGTGTTCACTCTGAATAATCCTTCCGAAGACGAGCGCAAGAAAATACGGGATCTTCCAATATCCCTATTTGATTATT**
Sequence_28	**ATCTGAAAACGAAATAAGTGATATAGTAGTATTACTATATTACTTAGG**

	**CAGCGGCAGCACCTCGGCAGCACCTCAGCAGCAACATGCCCAGCAAGAA GAATGGAAGAAGCGGACCCCAACCCCATAAAAGGTGGGTGTTCACTCTG AATAATCCTTCCGAAGACGAGCGCAAGAAAATACGGGATCTTCCAATAT CCCTATTTGATTATT**
Sequence_29	**ATCTGAAAACGAAAGAAGTGAGATATAGGTATTACTATCTCACTTCG GCAGCGGCAGCACCTCGGCAGCACCTCAGCAGCAACATGCCCAGCAAGA AGAATGGAAGAAGCGGACCCCAACCCCATAAAAGGTGGGTGTTCACTCT GAATAATCCTTCCGAAGACGAGCGCAAGAAAATACGGGATCTTCCAATA TCCCTATTTGATTATT**
Sequence_30	**ATCTGAAAACGAAAAAAATTCGCTATAAGTATTACTAGCGAATTTTGG CAGCGGCAGCACCTCGGCAGCACCTCAGCAGCAACATGCCCAGCAAGAA GAATGGAAGAAGCGGACCCCAACCCCATAAAAGGTGGGTGTTCACTCTG AATAATCCTTCCGAAGACGAGCGCAAGAAAATACGGGATCTTCCAATAT CCCTATTTGATTATT**
Sequence_31	**ATCTGAAAACGAAAGAAGTGCGATATAAGTATTACTATCGCACTTTG GCAGCGGCAGCACCTCGGCAGCACCTCAGCAGCAACATGCCCAGCAAGA AGAATGGAAGAAGCGGACCCCAACCCCATAAAAGGTGGGTGTTCACTCT GAATAATCCTTCCGAAGACGAGCGCAAGAAAATACGGGATCTTCCAATA TCCCTATTTGATTATT**
Sequence_32	**ATCTGAAAACGAAATAAGTAAAATATAAGTTTTACTATTTTACTTAGG CAGCGGCAGCACCTCGGCAGCACCTCAGCAGCAACATGCCCAGCAAGAA GAATGGAAGAAGCGGACCCCAACCCCATAAAAGGTGGGTGTTCACTCTG AATAATCCTTCCGAAGACGAGCGCAAGAAAATACGGGATCTTCCAATAT CCCTATTTGATTATT**
Sequence_33	**ATCTGAAAACGAAAGAAGTTCGATATATGTATTACTATCGAACTTCGG CAGCGGCAGCACCTCGGCAGCACCTCAGCAGCAACATGCCCAGCAAGAA GAATGGAAGAAGCGGACCCCAACCCCATAAAAGGTGGGTGTTCACTCTG AATAATCCTTCCGAAGACGAGCGCAAGAAAATACGGGATCTTCCAATAT CCCTATTTGATTATT**
Sequence_34	**ATCTGAAAACGAAAAAATGAGTTACTGTAGTATTACTAACTCGTTTGG CAGCGGCAGCACCTCGGCAGCACCTCAGCAGCAACATGCCCAGCAAGAA GAATGGAAGAAGCGGACCCCAACCCCATAAAAGGTGGGTGTTCACTCTG AATAATCCTTCCGAAGACGAGCGCAAGAAAATACGGGATCTTCCAATAT CCCTATTTGATTATT**
Sequence_35	**ATCTGAAAACGAAAGAAGTTCGCTATAAGTATTACTAGCGAACTTTG GCAGCGGCAGCACCTCGGCAGCACCTCAGCAGCAACATGCCCAGCAAGA AGAATGGAAGAAGCGGACCCCAACCCCATAAAAGGTGGGTGTTCACTCT GAATAATCCTTCCGAAGACGAGCGCAAGAAAATACGGGATCTTCCAATA TCCCTATTTGATTATT**
Sequence_36	**ATCTGAAAACGAAAAAAGTACGTTATATGTATTACTAACGTACTTTGG CAGCGGCAGCACCTCGGCAGCACCTCAGCAGCAACATGCCCAGCAAGAA GAATGGAAGAAGCGGACCCCAACCCCATAAAAGGTGGGTGTTCACTCTG AATAATCCTTCCGAAGACGAGCGCAAGAAAATACGGGATCTTCCAATAT**

	**CCCTATTTGATTATT**
Sequence_37	**ATCTGAAAACGAAATAAATTTTTTTGTAGTGTTACAAAAAAATTTAGG CAGCGGCAGCACCTCGGCAGCACCTCAGCAGCAACATGCCCAGCAAGAA GAATGGAAGAAGCGGACCCCAACCCCATAAAAGGTGGGTGTTCACTCTG AATAATCCTTCCGAAGACGAGCGCAAGAAAATACGGGATCTTCCAATAT CCCTATTTGATTATT**
Sequence_38	**ATCTGAAAACGAAAAAATTCTTTTCTGTAGTATTACAAAAGAATTTGG CAGCGGCAGCACCTCGGCAGCACCTCAGCAGCAACATGCCCAGCAAGAA GAATGGAAGAAGCGGACCCCAACCCCATAAAAGGTGGGTGTTCACTCTG AATAATCCTTCCGAAGACGAGCGCAAGAAAATACGGGATCTTCCAATAT CCCTATTTGATTATT**
Sequence_39	**ATCTGAAAACGAAAAAATGCTATACTGTAGTATTACTATAGCATTTGG CAGCGGCAGCACCTCGGCAGCACCTCAGCAGCAACATGCCCAGCAAGAA GAATGGAAGAAGCGGACCCCAACCCCATAAAAGGTGGGTGTTCACTCTG AATAATCCTTCCGAAGACGAGCGCAAGAAAATACGGGATCTTCCAATAT CCCTATTTGATTATT**
Sequence_40	**ATCTGAAAACGAAAAAAATTTAATTTAAGTATTACAATTGAATTTTGG CAGCGGCAGCACCTCGGCAGCACCTCAGCAGCAACATGCCCAGCAAGAA GAATGGAAGAAGCGGACCCCAACCCCATAAAAGGTGGGTGTTCACTCTG AATAATCCTTCCGAAGACGAGCGCAAGAAAATACGGGATCTTCCAATAT CCCTATTTGATTATT**
Sequence_41	**ATCTGAAAACGAAAGAAATTAAATAGTAGTATTACTATTTAGTTTCGG CAGCGGCAGCACCTCGGCAGCACCTCAGCAGCAACATGCCCAGCAAGAA GAATGGAAGAAGCGGACCCCAACCCCATAAAAGGTGGGTGTTCACTCTG AATAATCCTTCCGAAGACGAGCGCAAGAAAATACGGGATCTTCCAATAT CCCTATTTGATTATT**
Sequence_42	**ATCTGAAAACGAAAGAAGTGTGATAGTAGTATTACTATCACGCTTCG GCAGCGGCAGCACCTCGGCAGCACCTCAGCAGCAACATGCCCAGCAAGA AGAATGGAAGAAGCGGACCCCAACCCCATAAAAGGTGGGTGTTCACTCT GAATAATCCTTCCGAAGACGAGCGCAAGAAAATACGGGATCTTCCAATA TCCCTATTTGATTATT**
Sequence_43	**ATCTGAAAACGAAAGAAATGCGCTTGTAGTGTTACAAGCGCATTTCG GCAGCGGCAGCACCTCGGCAGCACCTCAGCAGCAACATGCCCAGCAAGA AGAATGGAAGAAGCGGACCCCAACCCCATAAAAGGTGGGTGTTCACTCT GAATAATCCTTCCGAAGACGAGCGCAAGAAAATACGGGATCTTCCAATA TCCCTATTTGATTATT**
Sequence_44	**ATCTGAAAACGAAAAAATTTTTACTGTAGTATTACTAAAAAATTTGG CAGCGGCAGCACCTCGGCAGCACCTCAGCAGCAACATGCCCAGCAAGAA GAATGGAAGAAGCGGACCCCAACCCCATAAAAGGTGGGTGTTCACTCTG AATAATCCTTCCGAAGACGAGCGCAAGAAAATACGGGATCTTCCAATAT CCCTATTTGATTATT**
Sequence_45	**ATCTGAAAACGAAATAAGTGCGCTATAAGTATTACTAGCGCATTTAG**

	**GCAGCGGCAGCACCTCGGCAGCACCTCAGCAGCAACATGCCCAGCAAGAAGAATGGAAGAAGCGGACCCCAACCCCATAAAAGGTGGGTGTTCACTCTGAATAATCCTTCCGAAGACGAGCGCAAGAAAATACGGGATCTTCCAATATCCCTATTTGATTATT**
Sequence_46	**ATCTGAAAACGAAAGAAGTGCGCTATAAGTATTACCAGTGCACTTTGGCAGCGGCAGCACCTCGGCAGCACCTCAGCAGCAACATGCCCAGCAAGAAGAATGGAAGAAGCGGACCCCAACCCCATAAAAGGTGGGTGTTCACTCTGAATAATCCTTCCGAAGACGAGCGCAAGAAAATACGGGATCTTCCAATATCCCTATTTGATTATT**
Sequence_47	**ATCTGAAAACGAAAGAAGTGCGCTGGATGTATTACCAGCGCACTTCGGCAGCGGCAGCACCTCGGCAGCACCTCAGCAGCAACATGCCCAGCAAGAAGAATGGAAGAAGCGGACCCCAACCCCATAAAAGGTGGGTGTTCACTCTGAATAATCCTTCCGAAGACGAGCGCAAGAAAATACGGGATCTTCCAATATCCCTATTTGATTATT**
Sequence_48	**ATCTGAAAACGAAAGAGGTGCGCTGTAAGTATTACGAGCGCACTTCGGCAGCGGCAGCACCTCGGCAGCACCTCAGCAGCAACATGCCCAGCAAGAAGAATGGAAGAAGCGGACCCCAACCCCATAAAAGGTGGGTGTTCACTCTGAATAATCCTTCCGAAGACGAGCGCAAGAAAATACGGGATCTTCCAATATCCCTATTTGATTATT**
Sequence_49	**ATCTGAAAACGAAAGAAGTGCGCTTGTAGTATTACCAGCGCACTTCGGCAGCGGCAGCACCTCGGCAGCACCTCAGCAGCAACATGCCCAGCAAGAAGAATGGAAGAAGCGGACCCCAACCCCATAAAAGGTGGGTGTTCACTCTGAATAATCCTTCCGAAGACGAGCGCAAGAAAATACGGGATCTTCCAATATCCCTATTTGATTATT**
Sequence_50	**ATCTGAAAACGAAAGAAGTGCGCTGCATGTATTAGCAGCGCACTTCGGCAGCGGCAGCACCTCGGCAGCACCTCAGCAGCAACATGCCCAGCAAGAAGAATGGAAGAAGCGGACCCCAACCCCATAAAAGGTGGGTGTTCACTCTGAATAATCCTTCCGAAGACGAGCGCAAGAAAATACGGGATCTTCCAATATCCCTATTTGATTATT**
Sequence_51	**ATCTGAAAACGAAAGAAGTGTGCTCTAAGTATTACAAGCGCACTTCGGCAGCGGCAGCACCTCGGCAGCACCTCAGCAGCAACATGCCCAGCAAGAAGAATGGAAGAAGCGGACCCCAACCCCATAAAAGGTGGGTGTTCACTCTGAATAATCCTTCCGAAGACGAGCGCAAGAAAATACGGGATCTTCCAATATCCCTATTTGATTATT**
Sequence_52	**ATCTGAAAACGAAAGAAGTGCGCTGGTAGTATTACGAGCGCACTTCGGCAGCGGCAGCACCTCGGCAGCACCTCAGCAGCAACATGCCCAGCAAGAAGAATGGAAGAAGCGGACCCCAACCCCATAAAAGGTGGGTGTTCACTCTGAATAATCCTTCCGAAGACGAGCGCAAGAAAATACGGGATCTTCCAATATCCCTATTTGATTATT**
Sequence_53	**ATCTGAAAACGAAAGAAGTGCGCTGTAAGTATTACGAGCGCGCTTCGGCAGCGGCAGCACCTCGGCAGCACCTCAGCAGCAACATGCCCAGCAAGAAGAATGGAAGAAGCGGACCCCAACCCCATAAAAGGTGGGTGTTCACTCTGAATAATCCTTCCGAAGACGAGCGCAAGAAAATACGGGATCTTCCAATA**

	**TCCCTATTTGATTATT**
Sequence_54	**ATCTGAAAACGAAAGAAGTGCGCTATAAGTATTACAAGTGCACTTTG GCAGCGGCAGCACCTCGGCAGCACCTCAGCAGCAACATGCCCAGCAAGA AGAATGGAAGAAGCGGACCCCAACCCCATAAAAGGTGGGTGTTCACTCT GAATAATCCTTCCGAAGACGAGCGCAAGAAAATACGGGATCTTCCAATA TCCCTATTTGATTATT**
Sequence_55	**ATCTGAAAACGAAAGAAGTGCGCTGAAAGTATTATCAGCGCATTTCG GCAGCGGCAGCACCTCGGCAGCACCTCAGCAGCAACATGCCCAGCAAGA AGAATGGAAGAAGCGGACCCCAACCCCATAAAAGGTGGGTGTTCACTCT GAATAATCCTTCCGAAGACGAGCGCAAGAAAATACGGGATCTTCCAATA TCCCTATTTGATTATT**
Sequence_56	**ATCTGAAAACGAAAGAAGTGCGCTGTAAGTATTAACAGCGCATTTCG GCAGCGGCAGCACCTCGGCAGCACCTCAGCAGCAACATGCCCAGCAAGA AGAATGGAAGAAGCGGACCCCAACCCCATAAAAGGTGGGTGTTCACTCT GAATAATCCTTCCGAAGACGAGCGCAAGAAAATACGGGATCTTCCAATA TCCCTATTTGATTATT**
Sequence_57	**ATCTGAAAACGAAAGAAGTGCGCTCTAAGTTTTACAAGCGCACTTCG GCAGCGGCAGCACCTCGGCAGCACCTCAGCAGCAACATGCCCAGCAAGA AGAATGGAAGAAGCGGACCCCAACCCCATAAAAGGTGGGTGTTCACTCT GAATAATCCTTCCGAAGACGAGCGCAAGAAAATACGGGATCTTCCAATA TCCCTATTTGATTATT**
Sequence_58	**ATCTGAAAACGAAAGAAGTGCGCTTGTAGTATTACGAGCGCGCTTCG GCAGCGGCAGCACCTCGGCAGCACCTCAGCAGCAACATGCCCAGCAAGA AGAATGGAAGAAGCGGACCCCAACCCCATAAAAGGTGGGTGTTCACTCT GAATAATCCTTCCGAAGACGAGCGCAAGAAAATACGGGATCTTCCAATA TCCCTATTTGATTATT**
Sequence_59	**ATCTGAAAACGAAAGAAGTGCGCTCTAAGTATTACAAGCGCATTTCG GCAGCGGCAGCACCTCGGCAGCACCTCAGCAGCAACATGCCCAGCAAGA AGAATGGAAGAAGCGGACCCCAACCCCATAAAAGGTGGGTGTTCACTCT GAATAATCCTTCCGAAGACGAGCGCAAGAAAATACGGGATCTTCCAATA TCCCTATTTGATTATT**
Sequence_60	**ATCTGAAAACGAAAGAAGTGCGCTGTAAGTATCACGAGCGCACTTCG GCAGCGGCAGCACCTCGGCAGCACCTCAGCAGCAACATGCCCAGCAAGA AGAATGGAAGAAGCGGACCCCAACCCCATAAAAGGTGGGTGTTCACTCT GAATAATCCTTCCGAAGACGAGCGCAAGAAAATACGGGATCTTCCAATA TCCCTATTTGATTATT**
Sequence_61	**ATCTGAAAACGAAAGAAGTGCGCTCTAAGTATTACTAGTGCACTTCG GCAGCGGCAGCACCTCGGCAGCACCTCAGCAGCAACATGCCCAGCAAGA AGAATGGAAGAAGCGGACCCCAACCCCATAAAAGGTGGGTGTTCACTCT GAATAATCCTTCCGAAGACGAGCGCAAGAAAATACGGGATCTTCCAATA TCCCTATTTGATTATT**
Sequence_62	**ATCTGAAAACGAAAGAAGTGCGCTCTATGTATTACTAGCGCACTTCGG**

	**CAGCGGCAGCACCTCGGCAGCACCTCAGCAGCAACATGCCCAGCAAGAAGAATGGAAGAAGCGGACCCCAACCCCATAAAAGGTGGGTGTTCACTCTGAATAATCCTTCCGAAGACGAGCGCAAGAAAATACGGGATCTTCCAATATCCCTATTTGATTATT**
Sequence_63	**ATCTGAAAACGAAAGAAGTGCGCTGTAGGTATTACTAGCGCACTTCGGCAGCGGCAGCACCTCGGCAGCACCTCAGCAGCAACATGCCCAGCAAGAAGAATGGAAGAAGCGGACCCCAACCCCATAAAAGGTGGGTGTTCACTCTGAATAATCCTTCCGAAGACGAGCGCAAGAAAATACGGGATCTTCCAATATCCCTATTTGATTATT**
Sequence_64	**ATCTGAAAACGAAAGAAGTGCGCTATAAGTATCACGAGCGCACTTCGGCAGCGGCAGCACCTCGGCAGCACCTCAGCAGCAACATGCCCAGCAAGAAGAATGGAAGAAGCGGACCCCAACCCCATAAAAGGTGGGTGTTCACTCTGAATAATCCTTCCGAAGACGAGCGCAAGAAAATACGGGATCTTCCAATATCCCTATTTGATTATT**
Sequence_65	**ATCTGAAAACGAAAGAAGTGCGCTTTAAGTATTACCAGTGCACTTTGGCAGCGGCAGCACCTCGGCAGCACCTCAGCAGCAACATGCCCAGCAAGAAGAATGGAAGAAGCGGACCCCAACCCCATAAAAGGTGGGTGTTCACTCTGAATAATCCTTCCGAAGACGAGCGCAAGAAAATACGGGATCTTCCAATATCCCTATTTGATTATT**
Sequence_66	**ATCTGAAAACGAAAGAAGTGCGCTGTAAGTATTACTAGCGCGCTTCGGCAGCGGCAGCACCTCGGCAGCACCTCAGCAGCAACATGCCCAGCAAGAAGAATGGAAGAAGCGGACCCCAACCCCATAAAAGGTGGGTGTTCACTCTGAATAATCCTTCCGAAGACGAGCGCAAGAAAATACGGGATCTTCCAATATCCCTATTTGATTATT**
Sequence_67	**ATCTGAAAACGAAAGAAGTGCGCTGATAGTATTATCAGCGCGCTTCGGCAGCGGCAGCACCTCGGCAGCACCTCAGCAGCAACATGCCCAGCAAGAAGAATGGAAGAAGCGGACCCCAACCCCATAAAAGGTGGGTGTTCACTCTGAATAATCCTTCCGAAGACGAGCGCAAGAAAATACGGGATCTTCCAATATCCCTATTTGATTATT**
Sequence_68	**ATCTGAAAACGAAAGAAGTGCGCTGTAAGTATTAACAGCGCACTTTGGCAGCGGCAGCACCTCGGCAGCACCTCAGCAGCAACATGCCCAGCAAGAAGAATGGAAGAAGCGGACCCCAACCCCATAAAAGGTGGGTGTTCACTCTGAATAATCCTTCCGAAGACGAGCGCAAGAAAATACGGGATCTTCCAATATCCCTATTTGATTATT**
Sequence_69	**ATCTGAAAACGAAAGAAGTGCGCTCTAAGTATTACCAGTGCACTTCGGCAGCGGCAGCACCTCGGCAGCACCTCAGCAGCAACATGCCCAGCAAGAAGAATGGAAGAAGCGGACCCCAACCCCATAAAAGGTGGGTGTTCACTCTGAATAATCCTTCCGAAGACGAGCGCAAGAAAATACGGGATCTTCCAATATCCCTATTTGATTATT**
Sequence_70	**ATCTGAAAACGAAAGAAGTGCGCTGTAAGTATTACCAGCGCACTTCGACAGCGGCGGCATCTCAGCAGCAGCTAGCAGCCAACATGCCCAGCAAGAAGAATGGAAGAAGCGGACCCCAACCCCATAAAAGGTGGGTGTTCACTCTGAATAATCCTTCCGAAGACGAGCGCAAGAAAATACGGGATCTTCCAATA**

	**TCCCTATTTGATTATT**
Sequence_71	**ATCTGAAAACGAAAGAAGTGCGCTGTAAGTATTACCAGCGCACTTCG GCGGCGCCAGCATCTCAGCAGCAGCTCAGCAGCAACATGCCCAGCAAGA AGAATGGAAGAAGCGGACCCCAACCCCATAAAAGGTGGGTGTTCACTCT GAATAATCCTTCCGAAGACGAGCGCAAGAAAATACGGGATCTTCCAATA TCCCTATTTGATTATT**
Sequence_72	**ATCTGAAAACGAAAGAAGTGCGCTGTAAGTATTACCAGCGCACTTCG GCGGGCGCAGCACATCGGCATTACCTCATCAGCAACATGCCCAGCAAGA AGAATGGAAGAAGCGGACCCCAACCCCATAAAAGGTGGGTGTTCACTCT GAATAATCCTTCCGAAGACGAGCGCAAGAAAATACGGGATCTTCCAATA TCCCTATTTGATTATT**
Sequence_73	**ATCTGAAAACGAAAGAAGTGCGCTGTAAGTATTACCAGCGCACTTCG ACAGCGCCAGCTCCTCGGCATCACTCCAGCATCAACATGCCCAGCAAGA AGAATGGAAGAAGCGGACCCCAACCCCATAAAAGGTGGGTGTTCACTCT GAATAATCCTTCCGAAGACGAGCGCAAGAAAATACGGGATCTTCCAATA TCCCTATTTGATTATT**
Sequence_74	**ATCTGAAAACGAAAGAAGTGCGCTGTAAGTATTACCAGCGCACTTCG GCGGCGGCAACTCCTTGGGAGCACTCAGCAGCCAACATGCCCAGCAAGA AGAATGGAAGAAGCGGACCCCAACCCCATAAAAGGTGGGTGTTCACTCT GAATAATCCTTCCGAAGACGAGCGCAAGAAAATACGGGATCTTCCAATA TCCCTATTTGATTATT**
Sequence_75	**ATCTGAAAACGAAAGAAGTGCGCTGTAAGTATTACCAGCGCACTTTG GGAGGCGCAGCACATTGGGAGCACTCAGCAGCCAACATGCCCAGCAAG AAGAATGGAAGAAGCGGACCCCAACCCCATAAAAGGTGGGTGTTCACT CTGAATAATCCTTCCGAAGACGAGCGCAAGAAAATACGGGATCTTCCAA TATCCCTATTTGATTATT**
Sequence_76	**ATCTGAAAACGAAAGAAGTGCGCTGTAAGTATTACCAGCGCACTTCG GCGGCGGCGGCTCCTTGGGAGTACCTAGCAGCCAACATGCCCAGCAAGA AGAATGGAAGAAGCGGACCCCAACCCCATAAAAGGTGGGTGTTCACTCT GAATAATCCTTCCGAAGACGAGCGCAAGAAAATACGGGATCTTCCAATA TCCCTATTTGATTATT**
Sequence_77	**ATCTGAAAACGAAAGAAGTGCGCTGTAAGTATTACCAGCGCACTTTG GGAGCGGCAACACATTGGGAGCACTCCAGCATCAACATGCCCAGCAAG AAGAATGGAAGAAGCGGACCCCAACCCCATAAAAGGTGGGTGTTCACT CTGAATAATCCTTCCGAAGACGAGCGCAAGAAAATACGGGATCTTCCAA TATCCCTATTTGATTATT**
Sequence_78	**ATCTGAAAACGAAAGAAGTGCGCTGTAAGTATTACCAGCGCACTTCG GCGGCGCCAGCTCCTCGGCACCACTCAGCAGCCAACATGCCCAGCAAGA AGAATGGAAGAAGCGGACCCCAACCCCATAAAAGGTGGGTGTTCACTCT GAATAATCCTTCCGAAGACGAGCGCAAGAAAATACGGGATCTTCCAATA TCCCTATTTGATTATT**
Sequence_79	**ATCTGAAAACGAAAGAAGTGCGCTGTAAGTATTACCAGCGCACTTCG**

	**GCGGCGGCAACACATCGGCATTACCTCAGCAGCAACATGCCCAGCAAGA<br>AGAATGGAAGAAGCGGACCCCAACCCCATAAAAGGTGGGTGTTCACTCT<br>GAATAATCCTTCCGAAGACGAGCGCAAGAAAATACGGGATCTTCCAATA<br>TCCCTATTTGATTATT**
Sequence_80	**ATCTGAAAACGAAAGAAGTGCGCTGTAAGTATTACCAGCGCACTTCG<br>ACAGCGGCAACACATCGGCACTACCTAGCAGCCAACATGCCCAGCAAGA<br>AGAATGGAAGAAGCGGACCCCAACCCCATAAAAGGTGGGTGTTCACTCT<br>GAATAATCCTTCCGAAGACGAGCGCAAGAAAATACGGGATCTTCCAATA<br>TCCCTATTTGATTATT**
Sequence_81	**ATCTGAAAACGAAAGAAGTGCGCTGTAAGTATTACCAGCGCACTTCG<br>GCGGCGCCAGCACATCGGCACCACTCCATCAGCAACATGCCCAGCAAGA<br>AGAATGGAAGAAGCGGACCCCAACCCCATAAAAGGTGGGTGTTCACTCT<br>GAATAATCCTTCCGAAGACGAGCGCAAGAAAATACGGGATCTTCCAATA<br>TCCCTATTTGATTATT**
Sequence_82	**ATCTGAAAACGAAAGAAGTGCGCTGTAAGTATTACCAGCGCACTTCG<br>GCGGCGGCGGCACATCGGCATCAGCTAGCAGCCAACATGCCCAGCAAG<br>AAGAATGGAAGAAGCGGACCCCAACCCCATAAAAGGTGGGTGTTCACT<br>CTGAATAATCCTTCCGAAGACGAGCGCAAGAAAATACGGGATCTTCCAA<br>TATCCCTATTTGATTATT**
Sequence_83	**ATCTGAAAACGAAAGAAGTGCGCTGTAAGTATTACCAGCGCACTTTG<br>GGAGCGGCAACTCCTTGGGAGCACTCAGCAGCCAACATGCCCAGCAAGA<br>AGAATGGAAGAAGCGGACCCCAACCCCATAAAAGGTGGGTGTTCACTCT<br>GAATAATCCTTCCGAAGACGAGCGCAAGAAAATACGGGATCTTCCAATA<br>TCCCTATTTGATTATT**
Sequence_84	**ATCTGAAAACGAAAGAAGTGCGCTGTAAGTATTACCAGCGCACTTCG<br>ACAGCGCCAGCTCCTCAGCAGCACTCCATCAGCAACATGCCCAGCAAGA<br>AGAATGGAAGAAGCGGACCCCAACCCCATAAAAGGTGGGTGTTCACTCT<br>GAATAATCCTTCCGAAGACGAGCGCAAGAAAATACGGGATCTTCCAATA<br>TCCCTATTTGATTATT**
Sequence_85	**ATCTGAAAACGAAAGAAGTGCGCTGTAAGTATTACCAGCGCACTTCG<br>GCGGCGGCAACATCTCAGCAGCAGCTCATCAGCAACATGCCCAGCAAGA<br>AGAATGGAAGAAGCGGACCCCAACCCCATAAAAGGTGGGTGTTCACTCT<br>GAATAATCCTTCCGAAGACGAGCGCAAGAAAATACGGGATCTTCCAATA<br>TCCCTATTTGATTATT**
Sequence_86	**ATCTGAAAACGAAAGAAGTGCGCTGTAAGTATTACCAGCGCACTTCG<br>ACAGCGGCGGCTCCTCAGCAGCACTCAGCAGCCAACATGCCCAGCAAGA<br>AGAATGGAAGAAGCGGACCCCAACCCCATAAAAGGTGGGTGTTCACTCT<br>GAATAATCCTTCCGAAGACGAGCGCAAGAAAATACGGGATCTTCCAATA<br>TCCCTATTTGATTATT**
Sequence_87	**ATCTGAAAACGAAAGAAGTGCGCTGTAAGTATTACCAGCGCACTTCG<br>ACAGGCGCAGCACATTGGGAGTACCTCAGCATCAACATGCCCAGCAAGA<br>AGAATGGAAGAAGCGGACCCCAACCCCATAAAAGGTGGGTGTTCACTCT<br>GAATAATCCTTCCGAAGACGAGCGCAAGAAAATACGGGATCTTCCAATA**

	**TCCCTATTTGATTATT**
Sequence_88	**ATCTGAAAACGAAAGAAGTGCGCTGTAAGTATTACCAGCGCACTTCG ACAGGCGCAGCTCCTCAGCAGCAGCTCATCAGCAACATGCCCAGCAAGA AGAATGGAAGAAGCGGACCCCAACCCCATAAAAGGTGGGTGTTCACTCT GAATAATCCTTCCGAAGACGAGCGCAAGAAAATACGGGATCTTCCAATA TCCCTATTTGATTATT**
Sequence_89	**ATCTGAAAACGAAAGAAGTGCGCTGTAAGTATTACCAGCGCACTTCG ACAGCGCCAGCTCCTTGGGAGCACTCAGCAGCCAACATGCCCAGCAAGA AGAATGGAAGAAGCGGACCCCAACCCCATAAAAGGTGGGTGTTCACTCT GAATAATCCTTCCGAAGACGAGCGCAAGAAAATACGGGATCTTCCAATA TCCCTATTTGATTATT**
Sequence_90	**ATCTGAAAACGAAAGAAGTGCGCTGTAAGTATTACCAGCGCACTTCG GCGGGCGCAGCATCTCGGCATCACTCCAGCATCAACATGCCCAGCAAGA AGAATGGAAGAAGCGGACCCCAACCCCATAAAAGGTGGGTGTTCACTCT GAATAATCCTTCCGAAGACGAGCGCAAGAAAATACGGGATCTTCCAATA TCCCTATTTGATTATT**
Sequence_91	**ATCTGAAAACGAAAGAAGTGCGCTGTAAGTATTACCAGCGCACTTTG GGAGCGGCGGCTCCTTGGGAGCACTCCATCAGCAACATGCCCAGCAAGA AGAATGGAAGAAGCGGACCCCAACCCCATAAAAGGTGGGTGTTCACTCT GAATAATCCTTCCGAAGACGAGCGCAAGAAAATACGGGATCTTCCAATA TCCCTATTTGATTATT**
Sequence_92	**ATCTGAAAACGAAAGAAGTGCGCTGTAAGTATTACCAGCGCACTTTG GGAGCGGCGGCACATTGGGAGCAGCTCAGCAGCAACATGCCCAGCAAG AAGAATGGAAGAAGCGGACCCCAACCCCATAAAAGGTGGGTGTTCACT CTGAATAATCCTTCCGAAGACGAGCGCAAGAAAATACGGGATCTTCCAA TATCCCTATTTGATTATT**
Sequence_93	**ATCTGAAAACGAAAGAAGTGCGCTGTAAGTATTACCAGCGCACTTCG ACAGCGGCGGCTCCTCGGCATCAGCTAGCAGCCAACATGCCCAGCAAGA AGAATGGAAGAAGCGGACCCCAACCCCATAAAAGGTGGGTGTTCACTCT GAATAATCCTTCCGAAGACGAGCGCAAGAAAATACGGGATCTTCCAATA TCCCTATTTGATTATT**
Sequence_94	**ATCTGAAAACGAAAGAAGTGCGCTGTAAGTATTACCAGCGCACTTCG GCGGCGGCGGCTCCTTGGGAGCACTCCATCAGCAACATGCCCAGCAAGA AGAATGGAAGAAGCGGACCCCAACCCCATAAAAGGTGGGTGTTCACTCT GAATAATCCTTCCGAAGACGAGCGCAAGAAAATACGGGATCTTCCAATA TCCCTATTTGATTATT**
Sequence_95	**ATCTGAAAACGAAAGAAGTGCGCTGTAAGTATTACCAGCGCACTTCG ACAGGCGCAGCTCCTCGGCACTACCTCAGCATCAACATGCCCAGCAAGA AGAATGGAAGAAGCGGACCCCAACCCCATAAAAGGTGGGTGTTCACTCT GAATAATCCTTCCGAAGACGAGCGCAAGAAAATACGGGATCTTCCAATA TCCCTATTTGATTATT**
Sequence_96	**ATCTGAAAACGAAAGAAGTGCGCTGTAAGTATTACCAGCGCACTTCG**

	**GCGGCGCCAGCACATCGGCATTACCTCAGCAGCAACATGCCCAGCAAGAAGAATGGAAGAAGCGGACCCCAACCCCATAAAAGGTGGGTGTTCACTCTGAATAATCCTTCCGAAGACGAGCGCAAGAAAATACGGGATCTTCCAATATCCCTATTTGATTATT**
Sequence_97	**ATCTGAAAACGAAAGAAGTGCGCTGTAAGTATTACCAGCGCACTTCGACAGGCGCAGCTCCTTGGGAGCACTCAGCAGCCAACATGCCCAGCAAGAAGAATGGAAGAAGCGGACCCCAACCCCATAAAAGGTGGGTGTTCACTCTGAATAATCCTTCCGAAGACGAGCGCAAGAAAATACGGGATCTTCCAATATCCCTATTTGATTATT**
Sequence_98	**ATCTGAAAACGAAAGAAGTGCGCTGTAAGTATTACCAGCGCACTTCGGCGGCGGCGGCTCCTCGGCACTACCTCATCAGCAACATGCCCAGCAAGAAGAATGGAAGAAGCGGACCCCAACCCCATAAAAGGTGGGTGTTCACTCTGAATAATCCTTCCGAAGACGAGCGCAAGAAAATACGGGATCTTCCAATATCCCTATTTGATTATT**
Sequence_99	**ATCTGAAAACGAAAGAAGTGCGCTGTAAGTATTACCAGCGCACTTTGGGAGCGGCAACACATCAGCAGCACTCCAGCAGCAACATGCCCAGCAAGAAGAATGGAAGAAGCGGACCCCAACCCCATAAAAGGTGGGTGTTCACTCTGAATAATCCTTCCGAAGACGAGCGCAAGAAAATACGGGATCTTCCAATATCCCTATTTGATTATT**
Sequence_100	**ATCTGAAAACGAAAGAAGTGCGCTGTAAGTATTACCAGCGCACTTTGGGAGCGCCAGCTCCTCAGCAGCACTCAGCAGCCAACATGCCCAGCAAGAAGAATGGAAGAAGCGGACCCCAACCCCATAAAAGGTGGGTGTTCACTCTGAATAATCCTTCCGAAGACGAGCGCAAGAAAATACGGGATCTTCCAATATCCCTATTTGATTATT**
Sequence_101	**ATCTGAAAACGAAAGAAGTGCGCTGTAAGTATTACCAGCGCACTTCGACAGCGCCAGCATCTCGGCACCACTCCAGCATCAACATGCCCAGCAAGAAGAATGGAAGAAGCGGACCCCAACCCCATAAAAGGTGGGTGTTCACTCTGAATAATCCTTCCGAAGACGAGCGCAAGAAAATACGGGATCTTCCAATATCCCTATTTGATTATT**
Sequence_102	**ATCTGAAAACGAAAGAAGTGCGCTGTAAGTATTACCAGCGCACTTTGGGAGCGGCAACATCTCAGCAGTACCTCAGCATCAACATGCCCAGCAAGAAGAATGGAAGAAGCGGACCCCAACCCCATAAAAGGTGGGTGTTCACTCTGAATAATCCTTCCGAAGACGAGCGCAAGAAAATACGGGATCTTCCAATATCCCTATTTGATTATT**
Sequence_103	**ATCTGAAAACGAAAGAAGTGCGCTGTAAGTATTACCAGCGCACTTTGGGAGCGGCGGCACATTGGGAGCACTCCAGCAGCAACATGCCCAGCAAGAAGAATGGAAGAAGCGGACCCCAACCCCATAAAAGGTGGGTGTTCACTCTGAATAATCCTTCCGAAGACGAGCGCAAGAAAATACGGGATCTTCCAATATCCCTATTTGATTATT**
Sequence_104	**ATCTGAAAACGAAAGAAGTGCGCTGTAAGTATTACCAGCGCACTTCGACAGCGGCGGCTCCTCGGCACCACTCCAGCATCAACATGCCCAGCAAGAAGAATGGAAGAAGCGGACCCCAACCCCATAAAAGGTGGGTGTTCACTCTGAATAATCCTTCCGAAGACGAGCGCAAGAAAATACGGGATCTTCCAATA**

	**TCCCTATTTGATTATT**
Sequence_105	**ATCTGAAAACGAAAGAAGTGCGCTGTAAGTATTACCAGCGCACTTCG ACAGCGCCAGCACATTGGGAGCAGCTAGCAGCCAACATGCCCAGCAAG AAGAATGGAAGAAGCGGACCCCAACCCCATAAAAGGTGGGTGTTCACT CTGAATAATCCTTCCGAAGACGAGCGCAAGAAAATACGGGATCTTCCAA TATCCCTATTTGATTATT**
Sequence_106	**ATCTGAAAACGAAAGAAGTGCGCTGTAAGTATTACCAGCGCACTTCG ACAGGCGCAGCTCCTCAGCAGCAGCTCAGCATCAACATGCCCAGCAAGA AGAATGGAAGAAGCGGACCCCAACCCCATAAAAGGTGGGTGTTCACTCT GAATAATCCTTCCGAAGACGAGCGCAAGAAAATACGGGATCTTCCAATA TCCCTATTTGATTATT**
Sequence_107	**ATCTGAAAACGAAAGAAGTGCGCTGTAAGTATTACCAGCGCACTTCG GCGGCGGCAACTCCTCAGCAGCACTCAGCAGCCAACATGCCCAGCAAGA AGAATGGAAGAAGCGGACCCCAACCCCATAAAAGGTGGGTGTTCACTCT GAATAATCCTTCCGAAGACGAGCGCAAGAAAATACGGGATCTTCCAATA TCCCTATTTGATTATT**
Sequence_108	**ATCTGAAAACGAAAGAAGTGCGCTGTAAGTATTACCAGCGCACTTTG GGAGCGGCGGCACATTGGGAGTACCTCATCAGCAACATGCCCAGCAAG AAGAATGGAAGAAGCGGACCCCAACCCCATAAAAGGTGGGTGTTCACT CTGAATAATCCTTCCGAAGACGAGCGCAAGAAAATACGGGATCTTCCAA TATCCCTATTTGATTATT**
Sequence_109	**ATCTGAAAACGAAAGAAGTGCGCTGTAAGTATTACCAGCGCACTTTG GGAGCGCCAGCACATCGGCACTACCTAGCAGCCAACATGCCCAGCAAGA AGAATGGAAGAAGCGGACCCCAACCCCATAAAAGGTGGGTGTTCACTCT GAATAATCCTTCCGAAGACGAGCGCAAGAAAATACGGGATCTTCCAATA TCCCTATTTGATTATT**
Sequence_110	**ATCTGAAAACGAAAGAAGTGCGCTGTAAGTATTACCAGCGCACTTCG GCGGCGGCAACACATCGGCACTACCTCAGCAGCAACATGCCCAGCAAGA AGAATGGAAGAAGCGGACCCCAACCCCATAAAAGGTGGGTGTTCACTCT GAATAATCCTTCCGAAGACGAGCGCAAGAAAATACGGGATCTTCCAATA TCCCTATTTGATTATT**
Sequence_111	**ATCTGAAAACGAAAGAAGTGCGCTGTAAGTATTACCAGCGCACTTCG ACAGGCGCAGCACATTGGGAGTACCTAGCAGCCAACATGCCCAGCAAG AAGAATGGAAGAAGCGGACCCCAACCCCATAAAAGGTGGGTGTTCACT CTGAATAATCCTTCCGAAGACGAGCGCAAGAAAATACGGGATCTTCCAA TATCCCTATTTGATTATT**
Sequence_112	**ATCTGAAAACGAAAGAAGTGCGCTGTAAGTATTACCAGCGCACTTTG GGAGCGGCGGCACATCGGCATCAGCTCATCAGCAACATGCCCAGCAAGA AGAATGGAAGAAGCGGACCCCAACCCCATAAAAGGTGGGTGTTCACTCT GAATAATCCTTCCGAAGACGAGCGCAAGAAAATACGGGATCTTCCAATA TCCCTATTTGATTATT**
Sequence_113	**ATCTGAAAACGAAAGAAGTGCGCTGTAAGTATTACCAGCGCACTTCG**

	**ACAGGCGCAGCACATCGGCATTACCTCATCAGCAACATGCCCAGCAAGA AGAATGGAAGAAGCGGACCCCAACCCCATAAAAGGTGGGTGTTCACTCT GAATAATCCTTCCGAAGACGAGCGCAAGAAAATACGGGATCTTCCAATA TCCCTATTTGATTATT**
Sequence_114	**ATCTGAAAACGAAAGAAGTGCGCTGTAAGTATTACCAGCGCACTTCG GCGGCGGCGGCTCCTCGGCACCAGCTCAGCAGCAACATGCCCAGCAAGA AGAATGGAAGAAGCGGACCCCAACCCCATAAAAGGTGGGTGTTCACTCT GAATAATCCTTCCGAAGACGAGCGCAAGAAAATACGGGATCTTCCAATA TCCCTATTTGATTATT**
Sequence_115	**ATCTGAAAACGAAAGAAGTGCGCTGTAAGTATTACCAGCGCACTTCG ACAGCGGCGGCATCTCGGCACCACTCCAGCAGCAACATGCCCAGCAAGA AGAATGGAAGAAGCGGACCCCAACCCCATAAAAGGTGGGTGTTCACTCT GAATAATCCTTCCGAAGACGAGCGCAAGAAAATACGGGATCTTCCAATA TCCCTATTTGATTATT**
Sequence_116	**ATCTGAAAACGAAAGAAGTGCGCTGTAAGTATTACCAGCGCACTTCG ACAGCGCCAGCTCCTTGGGAGCAGCTCAGCAGCAACATGCCCAGCAAGA AGAATGGAAGAAGCGGACCCCAACCCCATAAAAGGTGGGTGTTCACTCT GAATAATCCTTCCGAAGACGAGCGCAAGAAAATACGGGATCTTCCAATA TCCCTATTTGATTATT**
Sequence_117	**ATCTGAAAACGAAAGAAGTGCGCTGTAAGTATTACCAGCGCACTTCG ACAGGCGCAGCTCCTCGGCACTACCTCATCAGCAACATGCCCAGCAAGA AGAATGGAAGAAGCGGACCCCAACCCCATAAAAGGTGGGTGTTCACTCT GAATAATCCTTCCGAAGACGAGCGCAAGAAAATACGGGATCTTCCAATA TCCCTATTTGATTATT**
Sequence_118	**ATCTGAAAACGAAAGAAGTGCGCTGTAAGTATTACCAGCGCACTTCG GCGGCGGCGGCACATCAGCAGCAGCTCAGCAGCAACATGCCCAGCAAG AAGAATGGAAGAAGCGGACCCCAACCCCATAAAAGGTGGGTGTTCACT CTGAATAATCCTTCCGAAGACGAGCGCAAGAAAATACGGGATCTTCCAA TATCCCTATTTGATTATT**
Sequence_119	**ATCTGAAAACGAAAGAAGTGCGCTGTAAGTATTACCAGCGCACTTTG GGAGCGGCGGCTCCTCGGCACCACTCCAGCAGCAACATGCCCAGCAAGA AGAATGGAAGAAGCGGACCCCAACCCCATAAAAGGTGGGTGTTCACTCT GAATAATCCTTCCGAAGACGAGCGCAAGAAAATACGGGATCTTCCAATA TCCCTATTTGATTATT**
Sequence_120	**ATCTGAAAACGAAAGAAGTGCGCTGTAAGTATTACCAGCGCACTTTG GGAGCGGCAACATCTCAGCAGCACTCAGCAGCCAACATGCCCAGCAAGA AGAATGGAAGAAGCGGACCCCAACCCCATAAAAGGTGGGTGTTCACTCT GAATAATCCTTCCGAAGACGAGCGCAAGAAAATACGGGATCTTCCAATA TCCCTATTTGATTATT**
Sequence_121	**ATCTGAAAACGAAAGAAGTGCGCTGTAAGTATTACCAGCGCACTTCG ACAGGCGCAGCATCTTGGGAGTACCTCAGCAGCAACATGCCCAGCAAGA AGAATGGAAGAAGCGGACCCCAACCCCATAAAAGGTGGGTGTTCACTCT GAATAATCCTTCCGAAGACGAGCGCAAGAAAATACGGGATCTTCCAATA**

	**TCCCTATTTGATTATT**
Sequence_122	**ATCTGAAAACGAAAGAAGTGCGCTGTAAGTATTACCAGCGCACTTCG GCGGGCGCAGCACATCAGCAGCACTCCAGCATCAACATGCCCAGCAAGA AGAATGGAAGAAGCGGACCCCAACCCCATAAAAGGTGGGTGTTCACTCT GAATAATCCTTCCGAAGACGAGCGCAAGAAAATACGGGATCTTCCAATA TCCCTATTTGATTATT**
Sequence_123	**ATCTGAAAACGAAAGAAGTGCGCTGTAAGTATTACCAGCGCACTTCG GCGGCGGCAACTCCTCAGCAGTACCTCATCAGCAACATGCCCAGCAAGA AGAATGGAAGAAGCGGACCCCAACCCCATAAAAGGTGGGTGTTCACTCT GAATAATCCTTCCGAAGACGAGCGCAAGAAAATACGGGATCTTCCAATA TCCCTATTTGATTATT**
Sequence_124	**ATCTGAAAACGAAAGAAGTGCGCTGTAAGTATTACCAGCGCACTTCG ACAGCGGCAACACATCGGCACTACCTCAGCATCAACATGCCCAGCAAGA AGAATGGAAGAAGCGGACCCCAACCCCATAAAAGGTGGGTGTTCACTCT GAATAATCCTTCCGAAGACGAGCGCAAGAAAATACGGGATCTTCCAATA TCCCTATTTGATTATT**
Sequence_125	**ATCTGAAAACGAAAGAAGTGCGCTGTAAGTATTACCAGCGCACTTCG ACAGCGGCAACATCTCGGCATCACTCCAGCATCAACATGCCCAGCAAGA AGAATGGAAGAAGCGGACCCCAACCCCATAAAAGGTGGGTGTTCACTCT GAATAATCCTTCCGAAGACGAGCGCAAGAAAATACGGGATCTTCCAATA TCCCTATTTGATTATT**
Sequence_126	**ATCTGAAAACGAAAGAAGTGCGCTGTAAGTATTACCAGCGCACTTTG GGAGCGCCAGCTCCTTGGGAGTACCTCAGCAGCAACATGCCCAGCAAGA AGAATGGAAGAAGCGGACCCCAACCCCATAAAAGGTGGGTGTTCACTCT GAATAATCCTTCCGAAGACGAGCGCAAGAAAATACGGGATCTTCCAATA TCCCTATTTGATTATT**
Sequence_127	**ATCTGAAAACGAAAGAAGTGCGCTGTAAGTATTACCAGCGCACTTCG ACAGCGGCGGCACATCAGCAGCAGCTCAGCAGCAACATGCCCAGCAAG AAGAATGGAAGAAGCGGACCCCAACCCCATAAAAGGTGGGTGTTCACT CTGAATAATCCTTCCGAAGACGAGCGCAAGAAAATACGGGATCTTCCAA TATCCCTATTTGATTATT**
Sequence_128	**ATCTGAAAACGAAAGAAGTGCGCTGTAAGTATTACCAGCGCACTTTG GGAGCGGCAACATCTCAGCAGCACTCCAGCATCAACATGCCCAGCAAGA AGAATGGAAGAAGCGGACCCCAACCCCATAAAAGGTGGGTGTTCACTCT GAATAATCCTTCCGAAGACGAGCGCAAGAAAATACGGGATCTTCCAATA TCCCTATTTGATTATT**
Sequence_129	**ATCTGAAAACGAAAGAAGTGCGCTGTAAGTATTACCAGCGCACTTCG GCGGGCGCAGCACATCGGCATCAGCTAGCAGCCAACATGCCCAGCAAG AAGAATGGAAGAAGCGGACCCCAACCCCATAAAAGGTGGGTGTTCACT CTGAATAATCCTTCCGAAGACGAGCGCAAGAAAATACGGGATCTTCCAA TATCCCTATTTGATTATT**
Sequence_130	**ATCTGAAAACGAAAGAAGTGCGCTGTAAGTATTACCAGCGCACTTCG**

	**ACAGCGGCGGCACATCGGCATCAGCTCAGCAGCAACATGCCCAGCAAGAAGAATGGAAGAAGCGGACCCCAACCCCATAAAAGGTGGGTGTTCACTCTGAATAATCCTTCCGAAGACGAGCGCAAGAAAATACGGGATCTTCCAATATCCCTATTTGATTATT**
Sequence_131	**ATCTGAAAACGAAAGAAGTGCGCTGTAAGTATTACCAGCGCACTTCGACAGCGCCAGCTCCTCAGCAGCAGCTCAGCATCAACATGCCCAGCAAGAAGAATGGAAGAAGCGGACCCCAACCCCATAAAAGGTGGGTGTTCACTCTGAATAATCCTTCCGAAGACGAGCGCAAGAAAATACGGGATCTTCCAATATCCCTATTTGATTATT**
Sequence_132	**ATCTGAAAACGAAAGAAGTGCGCTGTAAGTATTACCAGCGCACTTCGGCGGCGGCAACATCTCAGCAGTACCTCAGCAGCAACATGCCCAGCAAGAAGAATGGAAGAAGCGGACCCCAACCCCATAAAAGGTGGGTGTTCACTCTGAATAATCCTTCCGAAGACGAGCGCAAGAAAATACGGGATCTTCCAATATCCCTATTTGATTATT**
Sequence_133	**ATCTGAAAACGAAAGAAGTGCGCTGTAAGTATTACCAGCGCACTTTGGGAGCGGCAACTCCTCAGCAGCAGCTCAGCATCAACATGCCCAGCAAGAAGAATGGAAGAAGCGGACCCCAACCCCATAAAAGGTGGGTGTTCACTCTGAATAATCCTTCCGAAGACGAGCGCAAGAAAATACGGGATCTTCCAATATCCCTATTTGATTATT**
Sequence_134	**ATCTGAAAACGAAAGAAGTGCGCTGTAAGTATTACCAGCGCACTTTGGGAGCGCCAGCATCTCGGCATCACTCAGCAGCCAACATGCCCAGCAAGAAGAATGGAAGAAGCGGACCCCAACCCCATAAAAGGTGGGTGTTCACTCTGAATAATCCTTCCGAAGACGAGCGCAAGAAAATACGGGATCTTCCAATATCCCTATTTGATTATT**
Sequence_135	**ATCTGAAAACGAAAGAAGTGCGCTGTAAGTATTACCAGCGCACTTCGGCGGCGGCGGCATCTCAGCAGCAGCTAGCAGCCAACATGCCCAGCAAGAAGAATGGAAGAAGCGGACCCCAACCCCATAAAAGGTGGGTGTTCACTCTGAATAATCCTTCCGAAGACGAGCGCAAGAAAATACGGGATCTTCCAATATCCCTATTTGATTATT**
Sequence_136	**ATCTGAAAACGAAAGAAGTGCGCTGTAAGTATTACCAGCGCACTTCGGCGGCGGCGGCACATTGGGAGCACTCCAGCATCAACATGCCCAGCAAGAAGAATGGAAGAAGCGGACCCCAACCCCATAAAAGGTGGGTGTTCACTCTGAATAATCCTTCCGAAGACGAGCGCAAGAAAATACGGGATCTTCCAATATCCCTATTTGATTATT**
Sequence_137	**ATCTGAAAACGAAAGAAGTGCGCTGTAAGTATTACCAGCGCACTTCGACAGCGGCAACATCTTGGGAGCACTCCAGCATCAACATGCCCAGCAAGAAGAATGGAAGAAGCGGACCCCAACCCCATAAAAGGTGGGTGTTCACTCTGAATAATCCTTCCGAAGACGAGCGCAAGAAAATACGGGATCTTCCAATATCCCTATTTGATTATT**
Sequence_138	**ATCTGAAAACGAAAGAAGTGCGCTGTAAGTATTACCAGCGCACTTTGGGAGGCGCAGCATCTTGGGAGTACCTCAGCAGCAACATGCCCAGCAAGAAGAATGGAAGAAGCGGACCCCAACCCCATAAAAGGTGGGTGTTCACTCTGAATAATCCTTCCGAAGACGAGCGCAAGAAAATACGGGATCTTCCAA**

	**TATCCCTATTTGATTATT**
Sequence_139	**ATCTGAAAACGAAAGAAGTGCGCTGTAAGTATTACCAGCGCACTTCG GCGGCGCCAGCTCCTCAGCAGCACTCCAGCAGCAACATGCCCAGCAAGA AGAATGGAAGAAGCGGACCCCAACCCCATAAAAGGTGGGTGTTCACTCT GAATAATCCTTCCGAAGACGAGCGCAAGAAAATACGGGATCTTCCAATA TCCCTATTTGATTATT**
Sequence_140	**ATCTGAAAACGAAAGAAGTGCGCTGTAAGTATTACCAGCGCACTTCG ACAGCGGCGGCATCTCGGCATTACCTCATCAGCAACATGCCCAGCAAGA AGAATGGAAGAAGCGGACCCCAACCCCATAAAAGGTGGGTGTTCACTCT GAATAATCCTTCCGAAGACGAGCGCAAGAAAATACGGGATCTTCCAATA TCCCTATTTGATTATT**
Sequence_141	**ATCTGAAAACGAAAGAAGTGCGCTGTAAGTATTACCAGCGCACTTTG GGAGCGCCAGCACATCGGCATCAGCTAGCAGCCAACATGCCCAGCAAGA AGAATGGAAGAAGCGGACCCCAACCCCATAAAAGGTGGGTGTTCACTCT GAATAATCCTTCCGAAGACGAGCGCAAGAAAATACGGGATCTTCCAATA TCCCTATTTGATTATT**
Sequence_142	**ATCTGAAAACGAAAGAAGTGCGCTGTAAGTATTACCAGCGCACTTTG GGAGCGGCGGCATCTCAGCAGCACTCCAGCAGCAACATGCCCAGCAAG AAGAATGGAAGAAGCGGACCCCAACCCCATAAAAGGTGGGTGTTCACT CTGAATAATCCTTCCGAAGACGAGCGCAAGAAAATACGGGATCTTCCAA TATCCCTATTTGATTATT**
Sequence_143	**ATCTGAAAACGAAAGAAGTGCGCTGTAAGTATTACCAGCGCACTTCG GCGGCGCCAGCACATCGGCACTACCTCAGCATCAACATGCCCAGCAAGA AGAATGGAAGAAGCGGACCCCAACCCCATAAAAGGTGGGTGTTCACTCT GAATAATCCTTCCGAAGACGAGCGCAAGAAAATACGGGATCTTCCAATA TCCCTATTTGATTATT**
Sequence_144	**ATCTGAAAACGAAAGAAGTGCGCTGTAAGTATTACCAGCGCACTTCG GCGGCGGCAACTCCTCGGCATTACCTAGCAGCCAACATGCCCAGCAAGA AGAATGGAAGAAGCGGACCCCAACCCCATAAAAGGTGGGTGTTCACTCT GAATAATCCTTCCGAAGACGAGCGCAAGAAAATACGGGATCTTCCAATA TCCCTATTTGATTATT**
Sequence_145	**ATCTGAAAACGAAAGAAGTGCGCTGTAAGTATTACCAGCGCACTTCG GCGGGCGCAGCACATTGGGAGCACTCCAGCAGCAACATGCCCAGCAAG AAGAATGGAAGAAGCGGACCCCAACCCCATAAAAGGTGGGTGTTCACT CTGAATAATCCTTCCGAAGACGAGCGCAAGAAAATACGGGATCTTCCAA TATCCCTATTTGATTATT**
Sequence_146	**ATCTGAAAACGAAAGAAGTGCGCTGTAAGTATTACCAGCGCACTTCG GCGGCGCCAGCACATCAGCAGCAGCTCAGCATCAACATGCCCAGCAAGA AGAATGGAAGAAGCGGACCCCAACCCCATAAAAGGTGGGTGTTCACTCT GAATAATCCTTCCGAAGACGAGCGCAAGAAAATACGGGATCTTCCAATA TCCCTATTTGATTATT**
Sequence_147	**ATCTGAAAACGAAAGAAGTGCGCTGTAAGTATTACCAGCGCACTTCG**

	GCGGCGGCAACATCTTGGGAGCAGCTCATCAGCAACATGCCCAGCAAGA AGAATGGAAGAAGCGGACCCCAACCCCATAAAAGGTGGGTGTTCACTCT GAATAATCCTTCCGAAGACGAGCGCAAGAAAATACGGGATCTTCCAATA TCCCTATTTGATTATT
Sequence_148	ATCTGAAAACGAAAGAAGTGCGCTGTAAGTATTACCAGCGCACTTCG GCGGCGGCGGCACATCGGCACTACCTCATCAGCAACATGCCCAGCAAGA AGAATGGAAGAAGCGGACCCCAACCCCATAAAAGGTGGGTGTTCACTCT GAATAATCCTTCCGAAGACGAGCGCAAGAAAATACGGGATCTTCCAATA TCCCTATTTGATTATT
Sequence_149	ATCTGAAAACGAAAGAAGTGCGCTGTAAGTATTACCAGCGCACTTTG GGAGCGGCAACACATCAGCAGCAGCTCATCAGCAACATGCCCAGCAAG AAGAATGGAAGAAGCGGACCCCAACCCCATAAAAGGTGGGTGTTCACT CTGAATAATCCTTCCGAAGACGAGCGCAAGAAAATACGGGATCTTCCAA TATCCCTATTTGATTATT
Sequence_150	ATCTGAAAACGAAAGAAGTGCGCTGTAAGTATTACCAGCGCACTTCG GCGGGCGCAGCACATCGGCATCAGCTCATCAGCAACATGCCCAGCAAGA AGAATGGAAGAAGCGGACCCCAACCCCATAAAAGGTGGGTGTTCACTCT GAATAATCCTTCCGAAGACGAGCGCAAGAAAATACGGGATCTTCCAATA TCCCTATTTGATTATT
Sequence_151	ATCTGAAAACGAAAGAAGTGCGCTGTAAGTATTACCAGCGCACTTTG GGAGCGGCGGCTCCTCGGCACCAGCTAGCAGCCAACATGCCCAGCAAGA AGAATGGAAGAAGCGGACCCCAACCCCATAAAAGGTGGGTGTTCACTCT GAATAATCCTTCCGAAGACGAGCGCAAGAAAATACGGGATCTTCCAATA TCCCTATTTGATTATT
Sequence_152	ATCTGAAAACGAAAGAAGTGCGCTGTAAGTATTACCAGCGCACTTCG GCGGCGGCAACACATCGGCATCAGCTAGCAGCCAACATGCCCAGCAAGA AGAATGGAAGAAGCGGACCCCAACCCCATAAAAGGTGGGTGTTCACTCT GAATAATCCTTCCGAAGACGAGCGCAAGAAAATACGGGATCTTCCAATA TCCCTATTTGATTATT
Sequence_153	ATCTGAAAACGAAAGAAGTGCGCTGTAAGTATTACCAGCGCACTTCG GCGGCGCCAGCATCTCGGCATCACTCCAGCATCAACATGCCCAGCAAGA AGAATGGAAGAAGCGGACCCCAACCCCATAAAAGGTGGGTGTTCACTCT GAATAATCCTTCCGAAGACGAGCGCAAGAAAATACGGGATCTTCCAATA TCCCTATTTGATTATT
Sequence_154	ATCTGAAAACGAAAGAAGTGCGCTGTAAGTATTACCAGCGCACTTCG GCGGCGGCAACATCTCGGCACCACTCCATCAGCAACATGCCCAGCAAGA AGAATGGAAGAAGCGGACCCCAACCCCATAAAAGGTGGGTGTTCACTCT GAATAATCCTTCCGAAGACGAGCGCAAGAAAATACGGGATCTTCCAATA TCCCTATTTGATTATT
Sequence_155	ATCTGAAAACGAAAGAAGTGCGCTGTAAGTATTACCAGCGCACTTTG GGAGGCGCAGCATCTCGGCATTACCTAGCAGCCAACATGCCCAGCAAGA AGAATGGAAGAAGCGGACCCCAACCCCATAAAAGGTGGGTGTTCACTCT GAATAATCCTTCCGAAGACGAGCGCAAGAAAATACGGGATCTTCCAATA

	**TCCCTATTTGATTATT**
Sequence_156	**ATCTGAAAACGAAAGAAGTGCGCTGTAAGTATTACCAGCGCACTTTG GGAGCGGCAACACATCGGCATCACTCAGCAGCCAACATGCCCAGCAAGA AGAATGGAAGAAGCGGACCCCAACCCCATAAAAGGTGGGTGTTCACTCT GAATAATCCTTCCGAAGACGAGCGCAAGAAAATACGGGATCTTCCAATA TCCCTATTTGATTATT**
Sequence_157	**ATCTGAAAACGAAAGAAGTGCGCTGTAAGTATTACCAGCGCACTTCG GCGGGCGCAGCATCTCGGCACCAGCTCAGCAGCAACATGCCCAGCAAGA AGAATGGAAGAAGCGGACCCCAACCCCATAAAAGGTGGGTGTTCACTCT GAATAATCCTTCCGAAGACGAGCGCAAGAAAATACGGGATCTTCCAATA TCCCTATTTGATTATT**
Sequence_158	**ATCTGAAAACGAAAGAAGTGCGCTGTAAGTATTACCAGCGCACTTCG GCGGCGGCGGCATCTCGGCACTACCTAGCAGCCAACATGCCCAGCAAGA AGAATGGAAGAAGCGGACCCCAACCCCATAAAAGGTGGGTGTTCACTCT GAATAATCCTTCCGAAGACGAGCGCAAGAAAATACGGGATCTTCCAATA TCCCTATTTGATTATT**
Sequence_159	**ATCTGAAAACGAAAGAAGTGCGCTGTAAGTATTACCAGCGCACTTTG GGAGGCGCAGCATCTCGGCACTACCTCAGCAGCAACATGCCCAGCAAGA AGAATGGAAGAAGCGGACCCCAACCCCATAAAAGGTGGGTGTTCACTCT GAATAATCCTTCCGAAGACGAGCGCAAGAAAATACGGGATCTTCCAATA TCCCTATTTGATTATT**
Sequence_160	**ATCTGAAAACGAAAGAAGTGCGCTGTAAGTATTACCAGCGCACTTTG GGAGCGCCAGCATCTCGGCACCACTCCAGCATCAACATGCCCAGCAAGA AGAATGGAAGAAGCGGACCCCAACCCCATAAAAGGTGGGTGTTCACTCT GAATAATCCTTCCGAAGACGAGCGCAAGAAAATACGGGATCTTCCAATA TCCCTATTTGATTATT**
Sequence_161	**ATCTGAAAACGAAAGAAGTGCGCTGTAAGTATTACCAGCGCACTTCG ACAGCGGCGGCATCTCAGCAGCACTCAGCAGCCAACATGCCCAGCAAGA AGAATGGAAGAAGCGGACCCCAACCCCATAAAAGGTGGGTGTTCACTCT GAATAATCCTTCCGAAGACGAGCGCAAGAAAATACGGGATCTTCCAATA TCCCTATTTGATTATT**
Sequence_162	**ATCTGAAAACGAAAGAAGTGCGCTGTAAGTATTACCAGCGCACTTTG GGAGCGGCGGCATCTTGGGAGCAGCTCAGCATCAACATGCCCAGCAAG AAGAATGGAAGAAGCGGACCCCAACCCCATAAAAGGTGGGTGTTCACT CTGAATAATCCTTCCGAAGACGAGCGCAAGAAAATACGGGATCTTCCAA TATCCCTATTTGATTATT**
Sequence_163	**ATCTGAAAACGAAAGAAGTGCGCTGTAAGTATTACCAGCGCACTTTG GGAGCGCCAGCACATCGGCATCACTCCAGCATCAACATGCCCAGCAAGA AGAATGGAAGAAGCGGACCCCAACCCCATAAAAGGTGGGTGTTCACTCT GAATAATCCTTCCGAAGACGAGCGCAAGAAAATACGGGATCTTCCAATA TCCCTATTTGATTATT**
Sequence_164	**ATCTGAAAACGAAAGAAGTGCGCTGTAAGTATTACCAGCGCACTTCG**

	ACAGCGCCAGCATCTCGGCACCAGCTAGCAGCCAACATGCCCAGCAAGAAGAATGGAAGAAGCGGACCCCAACCCCATAAAAGGTGGGTGTTCACTCTGAATAATCCTTCCGAAGACGAGCGCAAGAAAATACGGGATCTTCCAATATCCCTATTTGATTATT
Sequence_165	ATCTGAAAACGAAAGAAGTGCGCTGTAAGTATTACCAGCGCACTTCGACAGCGGCGGCATCTCGGCATTACCTAGCAGCCAACATGCCCAGCAAGAAGAATGGAAGAAGCGGACCCCAACCCCATAAAAGGTGGGTGTTCACTCTGAATAATCCTTCCGAAGACGAGCGCAAGAAAATACGGGATCTTCCAATATCCCTATTTGATTATT
Sequence_166	ATCTGAAAACGAAAGAAGTGCGCTGTAAGTATTACCAGCGCACTTCGACAGCGGCAACATCTTGGGAGCAGCTCAGCAGCAACATGCCCAGCAAGAAGAATGGAAGAAGCGGACCCCAACCCCATAAAAGGTGGGTGTTCACTCTGAATAATCCTTCCGAAGACGAGCGCAAGAAAATACGGGATCTTCCAATATCCCTATTTGATTATT
Sequence_167	ATCTGAAAACGAAAGAAGTGCGCTGTAAGTATTACCAGCGCACTTCGGCGGGCGCAGCACATTGGGAGCAGCTCAGCATCAACATGCCCAGCAAGAAGAATGGAAGAAGCGGACCCCAACCCCATAAAAGGTGGGTGTTCACTCTGAATAATCCTTCCGAAGACGAGCGCAAGAAAATACGGGATCTTCCAATATCCCTATTTGATTATT
Sequence_168	ATCTGAAAACGAAAGAAGTGCGCTGTAAGTATTACCAGCGCACTTTGGGAGCGGCAACATCTCGGCACCACTCAGCAGCCAACATGCCCAGCAAGAAGAATGGAAGAAGCGGACCCCAACCCCATAAAAGGTGGGTGTTCACTCTGAATAATCCTTCCGAAGACGAGCGCAAGAAAATACGGGATCTTCCAATATCCCTATTTGATTATT
Sequence_169	ATCTGAAAACGAAAGAAGTGCGCTGTAAGTATTACCAGCGCACTTTGGGAGCGGCAACACATCAGCAGCACTCAGCAGCCAACATGCCCAGCAAGAAGAATGGAAGAAGCGGACCCCAACCCCATAAAAGGTGGGTGTTCACTCTGAATAATCCTTCCGAAGACGAGCGCAAGAAAATACGGGATCTTCCAATATCCCTATTTGATTATT
Sequence_170	ATCTGAAAACGAAAGAAGTGCGCTGTAAGTATTACCAGCGCACTTTGGGAGCGGCAACACATTGGGAGCACTCAGCAGCCAACATGCCCAGCAAGAAGAATGGAAGAAGCGGACCCCAACCCCATAAAAGGTGGGTGTTCACTCTGAATAATCCTTCCGAAGACGAGCGCAAGAAAATACGGGATCTTCCAATATCCCTATTTGATTATT
Sequence_171	ATCTGAAAACGAAAGAAGTGCGCTGTAAGTATTACCAGCGCACTTCGACAGCGGCAACTCCTTGGGAGCACTCAGCAGCCAACATGCCCAGCAAGAAGAATGGAAGAAGCGGACCCCAACCCCATAAAAGGTGGGTGTTCACTCTGAATAATCCTTCCGAAGACGAGCGCAAGAAAATACGGGATCTTCCAATATCCCTATTTGATTATT
Sequence_172	ATCTGAAAACGAAAGAAGTGCGCTGTAAGTATTACCAGCGCACTTCGACAGGCGCAGCATCTCGGCATTACCTAGCAGCCAACATGCCCAGCAAGAAGAATGGAAGAAGCGGACCCCAACCCCATAAAAGGTGGGTGTTCACTCTGAATAATCCTTCCGAAGACGAGCGCAAGAAAATACGGGATCTTCCAATA

	**TCCCTATTTGATTATT**
Sequence_173	**ATCTGAAAACGAAAGAAGTGCGCTGTAAGTATTACCAGCGCACTTCG ACAGCGGCGGCACATTGGGAGCACTCAGCAGCCAACATGCCCAGCAAG AAGAATGGAAGAAGCGGACCCCAACCCCATAAAAGGTGGGTGTTCACT CTGAATAATCCTTCCGAAGACGAGCGCAAGAAAATACGGGATCTTCCAA TATCCCTATTTGATTATT**
Sequence_174	**ATCTGAAAACGAAAGAAGTGCGCTGTAAGTATTACCAGCGCACTTCG GCGGCGCCAGCTCCTCGGCATTACCTAGCAGCCAACATGCCCAGCAAGA AGAATGGAAGAAGCGGACCCCAACCCCATAAAAGGTGGGTGTTCACTCT GAATAATCCTTCCGAAGACGAGCGCAAGAAAATACGGGATCTTCCAATA TCCCTATTTGATTATT**
Sequence_175	**ATCTGAAAACGAAAGAAGTGCGCTGTAAGTATTACCAGCGCACTTTG GGAGCGCCAGCTCCTCGGCATCACTCCAGCATCAACATGCCCAGCAAGA AGAATGGAAGAAGCGGACCCCAACCCCATAAAAGGTGGGTGTTCACTCT GAATAATCCTTCCGAAGACGAGCGCAAGAAAATACGGGATCTTCCAATA TCCCTATTTGATTATT**
Sequence_176	**ATCTGAAAACGAAAGAAGTGCGCTGTAAGTATTACCAGCGCACTTCG ACAGCGGCGGCTCCTCGGCATCACTCCATCAGCAACATGCCCAGCAAGA AGAATGGAAGAAGCGGACCCCAACCCCATAAAAGGTGGGTGTTCACTCT GAATAATCCTTCCGAAGACGAGCGCAAGAAAATACGGGATCTTCCAATA TCCCTATTTGATTATT**
Sequence_177	**ATCTGAAAACGAAAGAAGTGCGCTGTAAGTATTACCAGCGCACTTCG ACAGCGGCGGCACATCGGCATCAGCTCAGCATCAACATGCCCAGCAAGA AGAATGGAAGAAGCGGACCCCAACCCCATAAAAGGTGGGTGTTCACTCT GAATAATCCTTCCGAAGACGAGCGCAAGAAAATACGGGATCTTCCAATA TCCCTATTTGATTATT**
Sequence_178	**ATCTGAAAACGAAAGAAGTGCGCTGTAAGTATTACCAGCGCACTTCG GCGGCGCCAGCATCTCGGCACCAGCTCAGCATCAACATGCCCAGCAAGA AGAATGGAAGAAGCGGACCCCAACCCCATAAAAGGTGGGTGTTCACTCT GAATAATCCTTCCGAAGACGAGCGCAAGAAAATACGGGATCTTCCAATA TCCCTATTTGATTATT**
Sequence_179	**ATCTGAAAACGAAAGAAGTGCGCTGTAAGTATTACCAGCGCACTTCG GCGGCGGCGGCATCTCGGCACTACCTCAGCATCAACATGCCCAGCAAGA AGAATGGAAGAAGCGGACCCCAACCCCATAAAAGGTGGGTGTTCACTCT GAATAATCCTTCCGAAGACGAGCGCAAGAAAATACGGGATCTTCCAATA TCCCTATTTGATTATT**
Sequence_180	**ATCTGAAAACGAAAGAAGTGCGCTGTAAGTATTACCAGCGCACTTCG ACAGCGCCAGCATCTCGGCATCAGCTAGCAGCCAACATGCCCAGCAAGA AGAATGGAAGAAGCGGACCCCAACCCCATAAAAGGTGGGTGTTCACTCT GAATAATCCTTCCGAAGACGAGCGCAAGAAAATACGGGATCTTCCAATA TCCCTATTTGATTATT**
Sequence_181	**ATCTGAAAACGAAAGAAGTGCGCTGTAAGTATTACCAGCGCACTTCG**

	**GCGGGCGCAGCACATCGGCATCACTCCATCAGCAACATGCCCAGCAAGAAGAATGGAAGAAGCGGACCCCAACCCCATAAAAGGTGGGTGTTCACTCTGAATAATCCTTCCGAAGACGAGCGCAAGAAAATACGGGATCTTCCAATATCCCTATTTGATTATT**
Sequence_182	**ATCTGAAAACGAAAGAAGTGCGCTGTAAGTATTACCAGCGCACTTCGGCGGCGGCAACATCTTGGGAGTACCTAGCAGCCAACATGCCCAGCAAGAAGAATGGAAGAAGCGGACCCCAACCCCATAAAAGGTGGGTGTTCACTCTGAATAATCCTTCCGAAGACGAGCGCAAGAAAATACGGGATCTTCCAATATCCCTATTTGATTATT**
Sequence_183	**ATCTGAAAACGAAAGAAGTGCGCTGTAAGTATTACCAGCGCACTTCGACAGCGGCGGCACATCGGCACCACTCCATCAGCAACATGCCCAGCAAGAAGAATGGAAGAAGCGGACCCCAACCCCATAAAAGGTGGGTGTTCACTCTGAATAATCCTTCCGAAGACGAGCGCAAGAAAATACGGGATCTTCCAATATCCCTATTTGATTATT**
Sequence_184	**ATCTGAAAACGAAAGAAGTGCGCTGTAAGTATTACCAGCGCACTTTGGGAGCGCCAGCACATTGGGAGCACTCCATCAGCAACATGCCCAGCAAGAAGAATGGAAGAAGCGGACCCCAACCCCATAAAAGGTGGGTGTTCACTCTGAATAATCCTTCCGAAGACGAGCGCAAGAAAATACGGGATCTTCCAATATCCCTATTTGATTATT**
Sequence_185	**ATCTGAAAACGAAAGAAGTGCGCTGTAAGTATTACCAGCGCACTTTGGGAGCGGCGGCTCCTCAGCAGCACTCAGCAGCCAACATGCCCAGCAAGAAGAATGGAAGAAGCGGACCCCAACCCCATAAAAGGTGGGTGTTCACTCTGAATAATCCTTCCGAAGACGAGCGCAAGAAAATACGGGATCTTCCAATATCCCTATTTGATTATT**
Sequence_186	**ATCTGAAAACGAAAGAAGTGCGCTGTAAGTATTACCAGCGCACTTCGGCGGCGGCGGCTCCTCGGCATTACCTCAGCAGCAACATGCCCAGCAAGAAGAATGGAAGAAGCGGACCCCAACCCCATAAAAGGTGGGTGTTCACTCTGAATAATCCTTCCGAAGACGAGCGCAAGAAAATACGGGATCTTCCAATATCCCTATTTGATTATT**
Sequence_187	**ATCTGAAAACGAAAGAAGTGCGCTGTAAGTATTACCAGCGCACTTCGGCGGCGGCAACATCTCGGCACCAGCTCATCAGCAACATGCCCAGCAAGAAGAATGGAAGAAGCGGACCCCAACCCCATAAAAGGTGGGTGTTCACTCTGAATAATCCTTCCGAAGACGAGCGCAAGAAAATACGGGATCTTCCAATATCCCTATTTGATTATT**
Sequence_188	**ATCTGAAAACGAAAGAAGTGCGCTGTAAGTATTACCAGCGCACTTTGGGAGGCGCAGCACATCGGCATCAGCTCAGCATCAACATGCCCAGCAAGAAGAATGGAAGAAGCGGACCCCAACCCCATAAAAGGTGGGTGTTCACTCTGAATAATCCTTCCGAAGACGAGCGCAAGAAAATACGGGATCTTCCAATATCCCTATTTGATTATT**
Sequence_189	**ATCTGAAAACGAAAGAAGTGCGCTGTAAGTATTACCAGCGCACTTCGGCGGCGGCGGCACATCGGCACTACCTCAGCATCAACATGCCCAGCAAGAAGAATGGAAGAAGCGGACCCCAACCCCATAAAAGGTGGGTGTTCACTCTGAATAATCCTTCCGAAGACGAGCGCAAGAAAATACGGGATCTTCCAATA**

	**TCCCTATTTGATTATT**
Sequence_190	**ATCTGAAAACGAAAGAAGTGCGCTGTAAGTATTACCAGCGCACTTTGGGAGCGGCAACACATCGGCACCACTCCATCAGCAACATGCCCAGCAAGAAGAATGGAAGAAGCGGACCCCAACCCCATAAAAGGTGGGTGTTCACTCTGAATAATCCTTCCGAAGACGAGCGCAAGAAAATACGGGATCTTCCAATATCCCTATTTGATTATT**
Sequence_191	**ATCTGAAAACGAAAGAAGTGCGCTGTAAGTATTACCAGCGCACTTTGGGAGGCGCAGCACATCAGCAGCAGCTAGCAGCCAACATGCCCAGCAAGAAGAATGGAAGAAGCGGACCCCAACCCCATAAAAGGTGGGTGTTCACTCTGAATAATCCTTCCGAAGACGAGCGCAAGAAAATACGGGATCTTCCAATATCCCTATTTGATTATT**
Sequence_192	**ATCTGAAAACGAAAGAAGTGCGCTGTAAGTATTACCAGCGCACTTTGGGAGCGCCAGCTCCTCGGCACCACTCCAGCATCAACATGCCCAGCAAGAAGAATGGAAGAAGCGGACCCCAACCCCATAAAAGGTGGGTGTTCACTCTGAATAATCCTTCCGAAGACGAGCGCAAGAAAATACGGGATCTTCCAATATCCCTATTTGATTATT**
Sequence_193	**ATCTGAAAACGAAAGAAGTGCGCTGTAAGTATTACCAGCGCACTTCGGCGGCGGCGGCACATTGGGAGCAGCTCAGCAGCAACATGCCCAGCAAGAAGAATGGAAGAAGCGGACCCCAACCCCATAAAAGGTGGGTGTTCACTCTGAATAATCCTTCCGAAGACGAGCGCAAGAAAATACGGGATCTTCCAATATCCCTATTTGATTATT**
Sequence_194	**ATCTGAAAACGAAAGAAGTGCGCTGTAAGTATTACCAGCGCACTTCGACAGCGGCGGCATCTCAGCAGCACTCCAGCATCAACATGCCCAGCAAGAAGAATGGAAGAAGCGGACCCCAACCCCATAAAAGGTGGGTGTTCACTCTGAATAATCCTTCCGAAGACGAGCGCAAGAAAATACGGGATCTTCCAATATCCCTATTTGATTATT**
Sequence_195	**ATCTGAAAACGAAAGAAGTGCGCTGTAAGTATTACCAGCGCACTTCGGCGGGCGCAGCATCTCGGCACTACCTCAGCATCAACATGCCCAGCAAGAAGAATGGAAGAAGCGGACCCCAACCCCATAAAAGGTGGGTGTTCACTCTGAATAATCCTTCCGAAGACGAGCGCAAGAAAATACGGGATCTTCCAATATCCCTATTTGATTATT**
Sequence_196	**ATCTGAAAACGAAAGAAGTGCGCTGTAAGTATTACCAGCGCACTTTGGGAGCGGCAACTCCTCAGCAGCACTCCAGCATCAACATGCCCAGCAAGAAGAATGGAAGAAGCGGACCCCAACCCCATAAAAGGTGGGTGTTCACTCTGAATAATCCTTCCGAAGACGAGCGCAAGAAAATACGGGATCTTCCAATATCCCTATTTGATTATT**
Sequence_197	**ATCTGAAAACGAAAGAAGTGCGCTGTAAGTATTACCAGCGCACTTCGACAGCGCCAGCACATCAGCAGCAGCTCATCAGCAACATGCCCAGCAAGAAGAATGGAAGAAGCGGACCCCAACCCCATAAAAGGTGGGTGTTCACTCTGAATAATCCTTCCGAAGACGAGCGCAAGAAAATACGGGATCTTCCAATATCCCTATTTGATTATT**
Sequence_198	**ATCTGAAAACGAAAGAAGTGCGCTGTAAGTATTACCAGCGCACTTCG**

	**GCGGCGGCGGCTCCTCGGCATCAGCTCAGCATCAACATGCCCAGCAAGAAGAATGGAAGAAGCGGACCCCAACCCCATAAAAGGTGGGTGTTCACTCTGAATAATCCTTCCGAAGACGAGCGCAAGAAAATACGGGATCTTCCAATATCCCTATTTGATTATT**
Sequence_199	**ATCTGAAAACGAAAGAAGTGCGCTGTAAGTATTACCAGCGCACTTCGACAGCGGCAACACATCAGCAGCAGCTCAGCATCAACATGCCCAGCAAGAAGAATGGAAGAAGCGGACCCCAACCCCATAAAAGGTGGGTGTTCACTCTGAATAATCCTTCCGAAGACGAGCGCAAGAAAATACGGGATCTTCCAATATCCCTATTTGATTATT**
Sequence_200	**ATCTGAAAACGAAAGAAGTGCGCTGTAAGTATTACCAGCGCACTTCGACAGCGGCAACACATTGGGAGCAGCTCATCAGCAACATGCCCAGCAAGAAGAATGGAAGAAGCGGACCCCAACCCCATAAAAGGTGGGTGTTCACTCTGAATAATCCTTCCGAAGACGAGCGCAAGAAAATACGGGATCTTCCAATATCCCTATTTGATTATT**
Sequence_201	**ATCTGAAAACGAAAGAAGTGCGCTGTAAGTATTACCAGCGCACTTCGACAGGCGCAGCACATTGGGAGCACTCCAGCATCAACATGCCCAGCAAGAAGAATGGAAGAAGCGGACCCCAACCCCATAAAAGGTGGGTGTTCACTCTGAATAATCCTTCCGAAGACGAGCGCAAGAAAATACGGGATCTTCCAATATCCCTATTTGATTATT**
Sequence_202	**ATCTGAAAACGAAAGAAGTGCGCTGTAAGTATTACCAGCGCACTTTGGGAGCGGCGGCACATCGGCACTACCTCAGCAGCAACATGCCCAGCAAGAAGAATGGAAGAAGCGGACCCCAACCCCATAAAAGGTGGGTGTTCACTCTGAATAATCCTTCCGAAGACGAGCGCAAGAAAATACGGGATCTTCCAATATCCCTATTTGATTATT**
Sequence_203	**ATCTGAAAACGAAAGAAGTGCGCTGTAAGTATTACCAGCGCACTTTGGGAGCGCCAGCATCTCGGCATTACCTAGCAGCCAACATGCCCAGCAAGAAGAATGGAAGAAGCGGACCCCAACCCCATAAAAGGTGGGTGTTCACTCTGAATAATCCTTCCGAAGACGAGCGCAAGAAAATACGGGATCTTCCAATATCCCTATTTGATTATT**
Sequence_204	**ATCTGAAAACGAAAGAAGTGCGCTGTAAGTATTACCAGCGCACTTCGACAGCGGCAACACATCGGCATCACTCCATCAGCAACATGCCCAGCAAGAAGAATGGAAGAAGCGGACCCCAACCCCATAAAAGGTGGGTGTTCACTCTGAATAATCCTTCCGAAGACGAGCGCAAGAAAATACGGGATCTTCCAATATCCCTATTTGATTATT**
Sequence_205	**ATCTGAAAACGAAAGAAGTGCGCTGTAAGTATTACCAGCGCACTTTGGGAGGCGCAGCATCTCAGCAGCACTCAGCAGCCAACATGCCCAGCAAGAAGAATGGAAGAAGCGGACCCCAACCCCATAAAAGGTGGGTGTTCACTCTGAATAATCCTTCCGAAGACGAGCGCAAGAAAATACGGGATCTTCCAATATCCCTATTTGATTATT**
Sequence_206	**ATCTGAAAACGAAAGAAGTGCGCTGTAAGTATTACCAGCGCACTTCGGCGGCGCCAGCACATCAGCAGCAGCTCATCAGCAACATGCCCAGCAAGAAGAATGGAAGAAGCGGACCCCAACCCCATAAAAGGTGGGTGTTCACTCTGAATAATCCTTCCGAAGACGAGCGCAAGAAAATACGGGATCTTCCAATA**

	**TCCCTATTTGATTATT**
Sequence_207	**ATCTGAAAACGAAAGAAGTGCGCTGTAAGTATTACCAGCGCACTTCG ACAGCGGCAACACATCAGCAGTACCTCATCAGCAACATGCCCAGCAAGA AGAATGGAAGAAGCGGACCCCAACCCCATAAAAGGTGGGTGTTCACTCT GAATAATCCTTCCGAAGACGAGCGCAAGAAAATACGGGATCTTCCAATA TCCCTATTTGATTATT**
Sequence_208	**ATCTGAAAACGAAAGAAGTGCGCTGTAAGTATTACCAGCGCACTTCG ACAGCGGCGGCTCCTCGGCACTACCTAGCAGCCAACATGCCCAGCAAGA AGAATGGAAGAAGCGGACCCCAACCCCATAAAAGGTGGGTGTTCACTCT GAATAATCCTTCCGAAGACGAGCGCAAGAAAATACGGGATCTTCCAATA TCCCTATTTGATTATT**
Sequence_209	**ATCTGAAAACGAAAGAAGTGCGCTGTAAGTATTACCAGCGCACTTCG GCGGGCGCAGCATCTTGGGAGCAGCTAGCAGCCAACATGCCCAGCAAG AAGAATGGAAGAAGCGGACCCCAACCCCATAAAAGGTGGGTGTTCACT CTGAATAATCCTTCCGAAGACGAGCGCAAGAAAATACGGGATCTTCCAA TATCCCTATTTGATTATT**
Sequence_210	**ATCTGAAAACGAAAGAAGTGCGCTGTAAGTATTACCAGCGCACTTCG GCGGCGCCAGCATCTCAGCAGTACCTCATCAGCAACATGCCCAGCAAGA AGAATGGAAGAAGCGGACCCCAACCCCATAAAAGGTGGGTGTTCACTCT GAATAATCCTTCCGAAGACGAGCGCAAGAAAATACGGGATCTTCCAATA TCCCTATTTGATTATT**
Sequence_211	**ATCTGAAAACGAAAGAAGTGCGCTGTAAGTATTACCAGCGCACTTCG ACAGCGGCAACATCTCAGCAGTACCTCAGCATCAACATGCCCAGCAAGA AGAATGGAAGAAGCGGACCCCAACCCCATAAAAGGTGGGTGTTCACTCT GAATAATCCTTCCGAAGACGAGCGCAAGAAAATACGGGATCTTCCAATA TCCCTATTTGATTATT**
Sequence_212	**ATCTGAAAACGAAAGAAGTGCGCTGTAAGTATTACCAGCGCACTTCG ACAGCGGCGGCATCTCGGCACCACTCCAGCATCAACATGCCCAGCAAGA AGAATGGAAGAAGCGGACCCCAACCCCATAAAAGGTGGGTGTTCACTCT GAATAATCCTTCCGAAGACGAGCGCAAGAAAATACGGGATCTTCCAATA TCCCTATTTGATTATT**
Sequence_213	**ATCTGAAAACGAAAGAAGTGCGCTGTAAGTATTACCAGCGCACTTCG ACAGCGGCGGCATCTTGGGAGTACCTCAGCATCAACATGCCCAGCAAGA AGAATGGAAGAAGCGGACCCCAACCCCATAAAAGGTGGGTGTTCACTCT GAATAATCCTTCCGAAGACGAGCGCAAGAAAATACGGGATCTTCCAATA TCCCTATTTGATTATT**
Sequence_214	**ATCTGAAAACGAAAGAAGTGCGCTGTAAGTATTACCAGCGCACTTTG GGAGCGGCAACATCTTGGGAGCACTCCAGCAGCAACATGCCCAGCAAG AAGAATGGAAGAAGCGGACCCCAACCCCATAAAAGGTGGGTGTTCACT CTGAATAATCCTTCCGAAGACGAGCGCAAGAAAATACGGGATCTTCCAA TATCCCTATTTGATTATT**
Sequence_215	**ATCTGAAAACGAAAGAAGTGCGCTGTAAGTATTACCAGCGCACTTCG**

	**GCGGCGCCAGCATCTCAGCAGTACCTCAGCAGCAACATGCCCAGCAAGAAGAATGGAAGAAGCGGACCCCAACCCCATAAAAGGTGGGTGTTCACTCTGAATAATCCTTCCGAAGACGAGCGCAAGAAAATACGGGATCTTCCAATATCCCTATTTGATTATT**
Sequence_216	**ATCTGAAAACGAAAGAAGTGCGCTGTAAGTATTACCAGCGCACTTCGGCGGCGGCAACATCTCGGCACTACCTCAGCAGCAACATGCCCAGCAAGAAGAATGGAAGAAGCGGACCCCAACCCCATAAAAGGTGGGTGTTCACTCTGAATAATCCTTCCGAAGACGAGCGCAAGAAAATACGGGATCTTCCAATATCCCTATTTGATTATT**
Sequence_217	**ATCTGAAAACGAAAGAAGTGCGCTGTAAGTATTACCAGCGCACTTTGGGAGCGCCAGCTCCTCAGCAGTACCTCATCAGCAACATGCCCAGCAAGAAGAATGGAAGAAGCGGACCCCAACCCCATAAAAGGTGGGTGTTCACTCTGAATAATCCTTCCGAAGACGAGCGCAAGAAAATACGGGATCTTCCAATATCCCTATTTGATTATT**
Sequence_218	**ATCTGAAAACGAAAGAAGTGCGCTGTAAGTATTACCAGCGCACTTCGGCGGGCGCAGCACATTGGGAGTACCTCAGCATCAACATGCCCAGCAAGAAGAATGGAAGAAGCGGACCCCAACCCCATAAAAGGTGGGTGTTCACTCTGAATAATCCTTCCGAAGACGAGCGCAAGAAAATACGGGATCTTCCAATATCCCTATTTGATTATT**
Sequence_219	**ATCTGAAAACGAAAGAAGTGCGCTGTAAGTATTACCAGCGCACTTTGGGAGGCGCAGCTCCTCGGCACCACTCCAGCATCAACATGCCCAGCAAGAAGAATGGAAGAAGCGGACCCCAACCCCATAAAAGGTGGGTGTTCACTCTGAATAATCCTTCCGAAGACGAGCGCAAGAAAATACGGGATCTTCCAATATCCCTATTTGATTATT**
Sequence_220	**ATCTGAAAACGAAAGAAGTGCGCTGTAAGTATTACCAGCGCACTTCGACAGCGCCAGCATCTCGGCATTACCTCAGCATCAACATGCCCAGCAAGAAGAATGGAAGAAGCGGACCCCAACCCCATAAAAGGTGGGTGTTCACTCTGAATAATCCTTCCGAAGACGAGCGCAAGAAAATACGGGATCTTCCAATATCCCTATTTGATTATT**
Sequence_221	**ATCTGAAAACGAAAGAAGTGCGCTGTAAGTATTACCAGCGCACTTTGGGAGGCGCAGCTCCTCAGCAGCACTCCAGCATCAACATGCCCAGCAAGAAGAATGGAAGAAGCGGACCCCAACCCCATAAAAGGTGGGTGTTCACTCTGAATAATCCTTCCGAAGACGAGCGCAAGAAAATACGGGATCTTCCAATATCCCTATTTGATTATT**
Sequence_222	**ATCTGAAAACGAAAGAAGTGCGCTGTAAGTATTACCAGCGCACTTCGACAGCGGCAACATCTCAGCAGCAGCTCATCAGCAACATGCCCAGCAAGAAGAATGGAAGAAGCGGACCCCAACCCCATAAAAGGTGGGTGTTCACTCTGAATAATCCTTCCGAAGACGAGCGCAAGAAAATACGGGATCTTCCAATATCCCTATTTGATTATT**
Sequence_223	**ATCTGAAAACGAAAGAAGTGCGCTGTAAGTATTACCAGCGCACTTCGACAGCGCCAGCATCTCGGCATCACTCCAGCATCAACATGCCCAGCAAGAAGAATGGAAGAAGCGGACCCCAACCCCATAAAAGGTGGGTGTTCACTCTGAATAATCCTTCCGAAGACGAGCGCAAGAAAATACGGGATCTTCCAATA**

	**TCCCTATTTGATTATT**
Sequence_224	**ATCTGAAAACGAAAGAAGTGCGCTGTAAGTATTACCAGCGCACTTCG ACAGCGGCGGCATCTCAGCAGCAGCTCAGCAGCAACATGCCCAGCAAGA AGAATGGAAGAAGCGGACCCCAACCCCATAAAAGGTGGGTGTTCACTCT GAATAATCCTTCCGAAGACGAGCGCAAGAAAATACGGGATCTTCCAATA TCCCTATTTGATTATT**
Sequence_225	**ATCTGAAAACGAAAGAAGTGCGCTGTAAGTATTACCAGCGCACTTCG ACAGCGCCAGCTCCTCAGCAGTACCTCATCAGCAACATGCCCAGCAAGA AGAATGGAAGAAGCGGACCCCAACCCCATAAAAGGTGGGTGTTCACTCT GAATAATCCTTCCGAAGACGAGCGCAAGAAAATACGGGATCTTCCAATA TCCCTATTTGATTATT**
Sequence_226	**ATCTGAAAACGAAAGAAGTGCGCTGTAAGTATTACCAGCGCACTTTG GGAGGCGCAGCTCCTTGGGAGCAGCTCAGCAGCAACATGCCCAGCAAG AAGAATGGAAGAAGCGGACCCCAACCCCATAAAAGGTGGGTGTTCACT CTGAATAATCCTTCCGAAGACGAGCGCAAGAAAATACGGGATCTTCCAA TATCCCTATTTGATTATT**
Sequence_227	**ATCTGAAAACGAAAGAAGTGCGCTGTAAGTATTACCAGCGCACTTCG ACAGCGCCAGCTCCTCGGCATCACTCAGCAGCCAACATGCCCAGCAAGA AGAATGGAAGAAGCGGACCCCAACCCCATAAAAGGTGGGTGTTCACTCT GAATAATCCTTCCGAAGACGAGCGCAAGAAAATACGGGATCTTCCAATA TCCCTATTTGATTATT**
Sequence_228	**ATCTGAAAACGAAAGAAGTGCGCTGTAAGTATTACCAGCGCACTTCG ACAGCGGCAACATCTCAGCAGCACTCAGCAGCCAACATGCCCAGCAAGA AGAATGGAAGAAGCGGACCCCAACCCCATAAAAGGTGGGTGTTCACTCT GAATAATCCTTCCGAAGACGAGCGCAAGAAAATACGGGATCTTCCAATA TCCCTATTTGATTATT**
Sequence_229	**ATCTGAAAACGAAAGAAGTGCGCTGTAAGTATTACCAGCGCACTTCG GCGGCGGCAACACATCGGCATTACCTAGCAGCCAACATGCCCAGCAAGA AGAATGGAAGAAGCGGACCCCAACCCCATAAAAGGTGGGTGTTCACTCT GAATAATCCTTCCGAAGACGAGCGCAAGAAAATACGGGATCTTCCAATA TCCCTATTTGATTATT**
Sequence_230	**ATCTGAAAACGAAAGAAGTGCGCTGTAAGTATTACCAGCGCACTTTG GGAGCGCCAGCACATTGGGAGCAGCTAGCAGCCAACATGCCCAGCAAG AAGAATGGAAGAAGCGGACCCCAACCCCATAAAAGGTGGGTGTTCACT CTGAATAATCCTTCCGAAGACGAGCGCAAGAAAATACGGGATCTTCCAA TATCCCTATTTGATTATT**
Sequence_231	**ATCTGAAAACGAAAGAAGTGCGCTGTAAGTATTACCAGCGCACTTTG GGAGCGGCAACACATCAGCAGTACCTCATCAGCAACATGCCCAGCAAGA AGAATGGAAGAAGCGGACCCCAACCCCATAAAAGGTGGGTGTTCACTCT GAATAATCCTTCCGAAGACGAGCGCAAGAAAATACGGGATCTTCCAATA TCCCTATTTGATTATT**
Sequence_232	**ATCTGAAAACGAAAGAAGTGCGCTGTAAGTATTACCAGCGCACTTCG**

	**ACAGCGCCAGCTCCTCGGCATCAGCTCAGCATCAACATGCCCAGCAAGA AGAATGGAAGAAGCGGACCCCAACCCCATAAAAGGTGGGTGTTCACTCT GAATAATCCTTCCGAAGACGAGCGCAAGAAAATACGGGATCTTCCAATA TCCCTATTTGATTATT**
Sequence_233	**ATCTGAAAACGAAAGAAGTGCGCTGTAAGTATTACCAGCGCACTTCG ACAGCGGCGGCACATCGGCATTACCTCATCAGCAACATGCCCAGCAAGA AGAATGGAAGAAGCGGACCCCAACCCCATAAAAGGTGGGTGTTCACTCT GAATAATCCTTCCGAAGACGAGCGCAAGAAAATACGGGATCTTCCAATA TCCCTATTTGATTATT**
Sequence_234	**ATCTGAAAACGAAAGAAGTGCGCTGTAAGTATTACCAGCGCACTTTG GGAGCGGCAACTCCTCAGCAGTACCTAGCAGCCAACATGCCCAGCAAGA AGAATGGAAGAAGCGGACCCCAACCCCATAAAAGGTGGGTGTTCACTCT GAATAATCCTTCCGAAGACGAGCGCAAGAAAATACGGGATCTTCCAATA TCCCTATTTGATTATT**
Sequence_235	**ATCTGAAAACGAAAGAAGTGCGCTGTAAGTATTACCAGCGCACTTCG GCGGCGGCGGCTCCTCGGCACCAGCTCATCAGCAACATGCCCAGCAAGA AGAATGGAAGAAGCGGACCCCAACCCCATAAAAGGTGGGTGTTCACTCT GAATAATCCTTCCGAAGACGAGCGCAAGAAAATACGGGATCTTCCAATA TCCCTATTTGATTATT**
Sequence_236	**ATCTGAAAACGAAAGAAGTGCGCTGTAAGTATTACCAGCGCACTTTG GGAGCGGCGGCATCTCGGCACCAGCTCAGCATCAACATGCCCAGCAAGA AGAATGGAAGAAGCGGACCCCAACCCCATAAAAGGTGGGTGTTCACTCT GAATAATCCTTCCGAAGACGAGCGCAAGAAAATACGGGATCTTCCAATA TCCCTATTTGATTATT**
Sequence_237	**ATCTGAAAACGAAAGAAGTGCGCTGTAAGTATTACCAGCGCACTTCG GCGGCGGCAACTCCTCGGCACCACTCAGCAGCCAACATGCCCAGCAAGA AGAATGGAAGAAGCGGACCCCAACCCCATAAAAGGTGGGTGTTCACTCT GAATAATCCTTCCGAAGACGAGCGCAAGAAAATACGGGATCTTCCAATA TCCCTATTTGATTATT**
Sequence_238	**ATCTGAAAACGAAAGAAGTGCGCTGTAAGTATTACCAGCGCACTTCG ACAGCGGCGGCTCCTCGGCACCAGCTAGCAGCCAACATGCCCAGCAAGA AGAATGGAAGAAGCGGACCCCAACCCCATAAAAGGTGGGTGTTCACTCT GAATAATCCTTCCGAAGACGAGCGCAAGAAAATACGGGATCTTCCAATA TCCCTATTTGATTATT**
Sequence_239	**ATCTGAAAACGAAAGAAGTGCGCTGTAAGTATTACCAGCGCACTTCG GCGGCGGCGGCTCCTCGGCACCACTCCAGCAGCAACATGCCCAGCAAGA AGAATGGAAGAAGCGGACCCCAACCCCATAAAAGGTGGGTGTTCACTCT GAATAATCCTTCCGAAGACGAGCGCAAGAAAATACGGGATCTTCCAATA TCCCTATTTGATTATT**
Sequence_240	**ATCTGAAAACGAAAGAAGTGCGCTGTAAGTATTACCAGCGCACTTCG ACAGCGGCAACATCTCGGCACCAGCTCAGCATCAACATGCCCAGCAAGA AGAATGGAAGAAGCGGACCCCAACCCCATAAAAGGTGGGTGTTCACTCT GAATAATCCTTCCGAAGACGAGCGCAAGAAAATACGGGATCTTCCAATA**

	**TCCCTATTTGATTATT**
Sequence_241	**ATCTGAAAACGAAAGAAGTGCGCTGTAAGTATTACCAGCGCACTTCG GCGGGCGCAGCATCTTGGGAGTACCTCAGCATCAACATGCCCAGCAAGA AGAATGGAAGAAGCGGACCCCAACCCCATAAAAGGTGGGTGTTCACTCT GAATAATCCTTCCGAAGACGAGCGCAAGAAAATACGGGATCTTCCAATA TCCCTATTTGATTATT**
Sequence_242	**ATCTGAAAACGAAAGAAGTGCGCTGTAAGTATTACCAGCGCACTTTG GGAGCGGCGGCACATTGGGAGCAGCTCATCAGCAACATGCCCAGCAAG AAGAATGGAAGAAGCGGACCCCAACCCCATAAAAGGTGGGTGTTCACT CTGAATAATCCTTCCGAAGACGAGCGCAAGAAAATACGGGATCTTCCAA TATCCCTATTTGATTATT**
Sequence_243	**ATCTGAAAACGAAAGAAGTGCGCTGTAAGTATTACCAGCGCACTTCG ACAGCGGCAACTCCTCGGCACTACCTCAGCATCAACATGCCCAGCAAGA AGAATGGAAGAAGCGGACCCCAACCCCATAAAAGGTGGGTGTTCACTCT GAATAATCCTTCCGAAGACGAGCGCAAGAAAATACGGGATCTTCCAATA TCCCTATTTGATTATT**
Sequence_244	**ATCTGAAAACGAAAGAAGTGCGCTGTAAGTATTACCAGCGCACTTTG GGAGCGCCAGCTCCTTGGGAGCACTCCATCAGCAACATGCCCAGCAAGA AGAATGGAAGAAGCGGACCCCAACCCCATAAAAGGTGGGTGTTCACTCT GAATAATCCTTCCGAAGACGAGCGCAAGAAAATACGGGATCTTCCAATA TCCCTATTTGATTATT**
Sequence_245	**ATCTGAAAACGAAAGAAGTGCGCTGTAAGTATTACCAGCGCACTTCG GCGGCGGCGGCATCTCAGCAGTACCTAGCAGCCAACATGCCCAGCAAGA AGAATGGAAGAAGCGGACCCCAACCCCATAAAAGGTGGGTGTTCACTCT GAATAATCCTTCCGAAGACGAGCGCAAGAAAATACGGGATCTTCCAATA TCCCTATTTGATTATT**
Sequence_246	**ATCTGAAAACGAAAGAAGTGCGCTGTAAGTATTACCAGCGCACTTCG GCGGCGGCGGCTCCTCAGCAGCAGCTCAGCATCAACATGCCCAGCAAGA AGAATGGAAGAAGCGGACCCCAACCCCATAAAAGGTGGGTGTTCACTCT GAATAATCCTTCCGAAGACGAGCGCAAGAAAATACGGGATCTTCCAATA TCCCTATTTGATTATT**
Sequence_247	**ATCTGAAAACGAAAGAAGTGCGCTGTAAGTATTACCAGCGCACTTTG GGAGCGGCGGCATCTCGGCATTACCTCATCAGCAACATGCCCAGCAAGA AGAATGGAAGAAGCGGACCCCAACCCCATAAAAGGTGGGTGTTCACTCT GAATAATCCTTCCGAAGACGAGCGCAAGAAAATACGGGATCTTCCAATA TCCCTATTTGATTATT**
Sequence_248	**ATCTGAAAACGAAAGAAGTGCGCTGTAAGTATTACCAGCGCACTTTG GGAGCGGCGGCACATCGGCACCACTCCAGCAGCAACATGCCCAGCAAG AAGAATGGAAGAAGCGGACCCCAACCCCATAAAAGGTGGGTGTTCACT CTGAATAATCCTTCCGAAGACGAGCGCAAGAAAATACGGGATCTTCCAA TATCCCTATTTGATTATT**
Sequence_249	**ATCTGAAAACGAAAGAAGTGCGCTGTAAGTATTACCAGCGCACTTCG**

	**ACAGCGCCAGCATCTTGGGAGCACTCAGCAGCCAACATGCCCAGCAAGA AGAATGGAAGAAGCGGACCCCAACCCCATAAAAGGTGGGTGTTCACTCT GAATAATCCTTCCGAAGACGAGCGCAAGAAAATACGGGATCTTCCAATA TCCCTATTTGATTATT**
Sequence_250	**ATCTGAAAACGAAAGAAGTGCGCTGTAAGTATTACCAGCGCACTTCG GCGGCGCCAGCTCCTTGGGAGTACCTCAGCATCAACATGCCCAGCAAGA AGAATGGAAGAAGCGGACCCCAACCCCATAAAAGGTGGGTGTTCACTCT GAATAATCCTTCCGAAGACGAGCGCAAGAAAATACGGGATCTTCCAATA TCCCTATTTGATTATT**
Sequence_251	**ATCTGAAAACGAAAGAAGTGCGCTGTAAGTATTACCAGCGCACTTTG GGAGCGGCAACATCTTGGGAGTACCTCAGCATCAACATGCCCAGCAAGA AGAATGGAAGAAGCGGACCCCAACCCCATAAAAGGTGGGTGTTCACTCT GAATAATCCTTCCGAAGACGAGCGCAAGAAAATACGGGATCTTCCAATA TCCCTATTTGATTATT**
Sequence_252	**ATCTGAAAACGAAAGAAGTGCGCTGTAAGTATTACCAGCGCACTTCG ACAGCGGCAACTCCTTGGGAGCAGCTCAGCAGCAACATGCCCAGCAAGA AGAATGGAAGAAGCGGACCCCAACCCCATAAAAGGTGGGTGTTCACTCT GAATAATCCTTCCGAAGACGAGCGCAAGAAAATACGGGATCTTCCAATA TCCCTATTTGATTATT**
Sequence_253	**ATCTGAAAACGAAAGAAGTGCGCTGTAAGTATTACCAGCGCACTTCG GCGGCGGCGGCATCTCGGCACCACTCAGCAGCCAACATGCCCAGCAAGA AGAATGGAAGAAGCGGACCCCAACCCCATAAAAGGTGGGTGTTCACTCT GAATAATCCTTCCGAAGACGAGCGCAAGAAAATACGGGATCTTCCAATA TCCCTATTTGATTATT**
Sequence_254	**ATCTGAAAACGAAAGAAGTGCGCTGTAAGTATTACCAGCGCACTTCG ACAGGCGCAGCTCCTCAGCAGCAGCTCAGCAGCAACATGCCCAGCAAGA AGAATGGAAGAAGCGGACCCCAACCCCATAAAAGGTGGGTGTTCACTCT GAATAATCCTTCCGAAGACGAGCGCAAGAAAATACGGGATCTTCCAATA TCCCTATTTGATTATT**
Sequence_255	**ATCTGAAAACGAAAGAAGTGCGCTGTAAGTATTACCAGCGCACTTCG ACAGGCGCAGCACATCGGCACCACTCCATCAGCAACATGCCCAGCAAGA AGAATGGAAGAAGCGGACCCCAACCCCATAAAAGGTGGGTGTTCACTCT GAATAATCCTTCCGAAGACGAGCGCAAGAAAATACGGGATCTTCCAATA TCCCTATTTGATTATT**
Sequence_256	**ATCTGAAAACGAAAGAAGTGCGCTGTAAGTATTACCAGCGCACTTCG ACAGCGGCGGCATCTCAGCAGTACCTCAGCATCAACATGCCCAGCAAGA AGAATGGAAGAAGCGGACCCCAACCCCATAAAAGGTGGGTGTTCACTCT GAATAATCCTTCCGAAGACGAGCGCAAGAAAATACGGGATCTTCCAATA TCCCTATTTGATTATT**
Sequence_257	**ATCTGAAAACGAAAGAAGTGCGCTGTAAGTATTACCAGCGCACTTCG GCGGCGGCGGCTCCTCAGCAGCAGCTAGCAGCCAACATGCCCAGCAAGA AGAATGGAAGAAGCGGACCCCAACCCCATAAAAGGTGGGTGTTCACTCT GAATAATCCTTCCGAAGACGAGCGCAAGAAAATACGGGATCTTCCAATA**

	**TCCCTATTTGATTATT**
Sequence_258	**ATCTGAAAACGAAAGAAGTGCGCTGTAAGTATTACCAGCGCACTTCG GCGGCGGCGGCACATTGGGAGTACCTCATCAGCAACATGCCCAGCAAGA AGAATGGAAGAAGCGGACCCCAACCCCATAAAAGGTGGGTGTTCACTCT GAATAATCCTTCCGAAGACGAGCGCAAGAAAATACGGGATCTTCCAATA TCCCTATTTGATTATT**
Sequence_259	**ATCTGAAAACGAAAGAAGTGCGCTGTAAGTATTACCAGCGCACTTCG GCGGCGGCAACACATTGGGAGCAGCTAGCAGCCAACATGCCCAGCAAG AAGAATGGAAGAAGCGGACCCCAACCCCATAAAAGGTGGGTGTTCACT CTGAATAATCCTTCCGAAGACGAGCGCAAGAAAATACGGGATCTTCCAA TATCCCTATTTGATTATT**
Sequence_260	**ATCTGAAAACGAAAGAAGTGCGCTGTAAGTATTACCAGCGCACTTCG GCGGCGCCAGCATCTTGGGAGCACTCAGCAGCCAACATGCCCAGCAAGA AGAATGGAAGAAGCGGACCCCAACCCCATAAAAGGTGGGTGTTCACTCT GAATAATCCTTCCGAAGACGAGCGCAAGAAAATACGGGATCTTCCAATA TCCCTATTTGATTATT**
Sequence_261	**ATCTGAAAACGAAAGAAGTGCGCTGTAAGTATTACCAGCGCACTTCG ACAGCGGCGGCTCCTTGGGAGTACCTCAGCATCAACATGCCCAGCAAGA AGAATGGAAGAAGCGGACCCCAACCCCATAAAAGGTGGGTGTTCACTCT GAATAATCCTTCCGAAGACGAGCGCAAGAAAATACGGGATCTTCCAATA TCCCTATTTGATTATT**
Sequence_262	**ATCTGAAAACGAAAGAAGTGCGCTGTAAGTATTACCAGCGCACTTCG ACAGCGCCAGCATCTCGGCACCACTCCAGCAGCAACATGCCCAGCAAGA AGAATGGAAGAAGCGGACCCCAACCCCATAAAAGGTGGGTGTTCACTCT GAATAATCCTTCCGAAGACGAGCGCAAGAAAATACGGGATCTTCCAATA TCCCTATTTGATTATT**
Sequence_263	**ATCTGAAAACGAAAGAAGTGCGCTGTAAGTATTACCAGCGCACTTCG ACAGCGGCAACACATCGGCACCACTCCAGCATCAACATGCCCAGCAAGA AGAATGGAAGAAGCGGACCCCAACCCCATAAAAGGTGGGTGTTCACTCT GAATAATCCTTCCGAAGACGAGCGCAAGAAAATACGGGATCTTCCAATA TCCCTATTTGATTATT**
Sequence_264	**ATCTGAAAACGAAAGAAGTGCGCTGTAAGTATTACCAGCGCACTTCG GCGGCGGCAACTCCTTGGGAGCAGCTCAGCATCAACATGCCCAGCAAGA AGAATGGAAGAAGCGGACCCCAACCCCATAAAAGGTGGGTGTTCACTCT GAATAATCCTTCCGAAGACGAGCGCAAGAAAATACGGGATCTTCCAATA TCCCTATTTGATTATT**
Sequence_265	**ATCTGAAAACGAAAGAAGTGCGCTGTAAGTATTACCAGCGCACTTCG ACAGGCGCAGCATCTTGGGAGCACTCCAGCATCAACATGCCCAGCAAGA AGAATGGAAGAAGCGGACCCCAACCCCATAAAAGGTGGGTGTTCACTCT GAATAATCCTTCCGAAGACGAGCGCAAGAAAATACGGGATCTTCCAATA TCCCTATTTGATTATT**
Sequence_266	**ATCTGAAAACGAAAGAAGTGCGCTGTAAGTATTACCAGCGCACTTCG**

	**ACAGGCGCAGCATCTCAGCAGTACCTAGCAGCCAACATGCCCAGCAAGAAGAATGGAAGAAGCGGACCCCAACCCCATAAAAGGTGGGTGTTCACTCTGAATAATCCTTCCGAAGACGAGCGCAAGAAAATACGGGATCTTCCAATATCCCTATTTGATTATT**
Sequence_267	**ATCTGAAAACGAAAGAAGTGCGCTGTAAGTATTACCAGCGCACTTTGGGAGCGGCAACATCTCGGCATCAGCTAGCAGCCAACATGCCCAGCAAGAAGAATGGAAGAAGCGGACCCCAACCCCATAAAAGGTGGGTGTTCACTCTGAATAATCCTTCCGAAGACGAGCGCAAGAAAATACGGGATCTTCCAATATCCCTATTTGATTATT**
Sequence_268	**ATCTGAAAACGAAAGAAGTGCGCTGTAAGTATTACCAGCGCACTTCGGCGGCGCCAGCACATCAGCAGTACCTCAGCATCAACATGCCCAGCAAGAAGAATGGAAGAAGCGGACCCCAACCCCATAAAAGGTGGGTGTTCACTCTGAATAATCCTTCCGAAGACGAGCGCAAGAAAATACGGGATCTTCCAATATCCCTATTTGATTATT**
Sequence_269	**ATCTGAAAACGAAAGAAGTGCGCTGTAAGTATTACCAGCGCACTTCGACAGCGGCGGCACATCAGCAGTACCTCATCAGCAACATGCCCAGCAAGAAGAATGGAAGAAGCGGACCCCAACCCCATAAAAGGTGGGTGTTCACTCTGAATAATCCTTCCGAAGACGAGCGCAAGAAAATACGGGATCTTCCAATATCCCTATTTGATTATT**
Sequence_270	**ATCTGAAAACGAAAGAAGTGCGCTGTAAGTATTACCAGCGCACTTCGGCGGGCGCAGCACATCGGCATTACCTAGCAGCCAACATGCCCAGCAAGAAGAATGGAAGAAGCGGACCCCAACCCCATAAAAGGTGGGTGTTCACTCTGAATAATCCTTCCGAAGACGAGCGCAAGAAAATACGGGATCTTCCAATATCCCTATTTGATTATT**
Sequence_271	**ATCTGAAAACGAAAGAAGTGCGCTGTAAGTATTACCAGCGCACTTTGGGAGGCGCAGCACATCGGCATCAGCTAGCAGCCAACATGCCCAGCAAGAAGAATGGAAGAAGCGGACCCCAACCCCATAAAAGGTGGGTGTTCACTCTGAATAATCCTTCCGAAGACGAGCGCAAGAAAATACGGGATCTTCCAATATCCCTATTTGATTATT**
Sequence_272	**ATCTGAAAACGAAAGAAGTGCGCTGTAAGTATTACCAGCGCACTTCGGCGGCGGCGGCTCCTCGGCATTACCTAGCAGCCAACATGCCCAGCAAGAAGAATGGAAGAAGCGGACCCCAACCCCATAAAAGGTGGGTGTTCACTCTGAATAATCCTTCCGAAGACGAGCGCAAGAAAATACGGGATCTTCCAATATCCCTATTTGATTATT**
Sequence_273	**ATCTGAAAACGAAAGAAGTGCGCTGTAAGTATTACCAGCGCACTTTGGGAGCGCCAGCACATTGGGAGTACCTCAGCAGCAACATGCCCAGCAAGAAGAATGGAAGAAGCGGACCCCAACCCCATAAAAGGTGGGTGTTCACTCTGAATAATCCTTCCGAAGACGAGCGCAAGAAAATACGGGATCTTCCAATATCCCTATTTGATTATT**
Sequence_274	**ATCTGAAAACGAAAGAAGTGCGCTGTAAGTATTACCAGCGCACTTCGACAGCGGCAACTCCTCAGCAGCAGCTCAGCAGCAACATGCCCAGCAAGAAGAATGGAAGAAGCGGACCCCAACCCCATAAAAGGTGGGTGTTCACTCTGAATAATCCTTCCGAAGACGAGCGCAAGAAAATACGGGATCTTCCAATA**

	**TCCCTATTTGATTATT**
Sequence_275	**ATCTGAAAACGAAAGAAGTGCGCTGTAAGTATTACCAGCGCACTTTG GGAGCGGCAACTCCTTGGGAGCAGCTCAGCATCAACATGCCCAGCAAGA AGAATGGAAGAAGCGGACCCCAACCCCATAAAAGGTGGGTGTTCACTCT GAATAATCCTTCCGAAGACGAGCGCAAGAAAATACGGGATCTTCCAATA TCCCTATTTGATTATT**
Sequence_276	**ATCTGAAAACGAAAGAAGTGCGCTGTAAGTATTACCAGCGCACTTTG GGAGCGGCGGCTCCTCGGCACTACCTAGCAGCCAACATGCCCAGCAAGA AGAATGGAAGAAGCGGACCCCAACCCCATAAAAGGTGGGTGTTCACTCT GAATAATCCTTCCGAAGACGAGCGCAAGAAAATACGGGATCTTCCAATA TCCCTATTTGATTATT**
Sequence_277	**ATCTGAAAACGAAAGAAGTGCGCTGTAAGTATTACCAGCGCACTTTG GGAGGCGCAGCATCTCAGCAGCAGCTCATCAGCAACATGCCCAGCAAGA AGAATGGAAGAAGCGGACCCCAACCCCATAAAAGGTGGGTGTTCACTCT GAATAATCCTTCCGAAGACGAGCGCAAGAAAATACGGGATCTTCCAATA TCCCTATTTGATTATT**
Sequence_278	**ATCTGAAAACGAAAGAAGTGCGCTGTAAGTATTACCAGCGCACTTCG GCGGGCGCAGCTCCTCAGCAGCACTCCATCAGCAACATGCCCAGCAAGA AGAATGGAAGAAGCGGACCCCAACCCCATAAAAGGTGGGTGTTCACTCT GAATAATCCTTCCGAAGACGAGCGCAAGAAAATACGGGATCTTCCAATA TCCCTATTTGATTATT**
Sequence_279	**ATCTGAAAACGAAAGAAGTGCGCTGTAAGTATTACCAGCGCACTTTG GGAGCGCCAGCATCTTGGGAGCAGCTCAGCATCAACATGCCCAGCAAGA AGAATGGAAGAAGCGGACCCCAACCCCATAAAAGGTGGGTGTTCACTCT GAATAATCCTTCCGAAGACGAGCGCAAGAAAATACGGGATCTTCCAATA TCCCTATTTGATTATT**
Sequence_280	**ATCTGAAAACGAAAGAAGTGCGCTGTAAGTATTACCAGCGCACTTCG ACAGCGGCAACATCTCGGCACCAGCTAGCAGCCAACATGCCCAGCAAGA AGAATGGAAGAAGCGGACCCCAACCCCATAAAAGGTGGGTGTTCACTCT GAATAATCCTTCCGAAGACGAGCGCAAGAAAATACGGGATCTTCCAATA TCCCTATTTGATTATT**
Sequence_281	**ATCTGAAAACGAAAGAAGTGCGCTGTAAGTATTACCAGCGCACTTTG GGAGGCGCAGCACATCGGCATTACCTCAGCATCAACATGCCCAGCAAGA AGAATGGAAGAAGCGGACCCCAACCCCATAAAAGGTGGGTGTTCACTCT GAATAATCCTTCCGAAGACGAGCGCAAGAAAATACGGGATCTTCCAATA TCCCTATTTGATTATT**
Sequence_282	**ATCTGAAAACGAAAGAAGTGCGCTGTAAGTATTACCAGCGCACTTTG GGAGCGGCGGCATCTCGGCATTACCTCAGCAGCAACATGCCCAGCAAGA AGAATGGAAGAAGCGGACCCCAACCCCATAAAAGGTGGGTGTTCACTCT GAATAATCCTTCCGAAGACGAGCGCAAGAAAATACGGGATCTTCCAATA TCCCTATTTGATTATT**
Sequence_283	**ATCTGAAAACGAAAGAAGTGCGCTGTAAGTATTACCAGCGCACTTCG**

	**ACAGCGCCAGCATCTCAGCAGCAGCTAGCAGCCAACATGCCCAGCAAGA AGAATGGAAGAAGCGGACCCCAACCCCATAAAAGGTGGGTGTTCACTCT GAATAATCCTTCCGAAGACGAGCGCAAGAAAATACGGGATCTTCCAATA TCCCTATTTGATTATT**
Sequence_284	**ATCTGAAAACGAAAGAAGTGCGCTGTAAGTATTACCAGCGCACTTTG GGAGCGCCAGCATCTTGGGAGTACCTAGCAGCCAACATGCCCAGCAAGA AGAATGGAAGAAGCGGACCCCAACCCCATAAAAGGTGGGTGTTCACTCT GAATAATCCTTCCGAAGACGAGCGCAAGAAAATACGGGATCTTCCAATA TCCCTATTTGATTATT**
Sequence_285	**ATCTGAAAACGAAAGAAGTGCGCTGTAAGTATTACCAGCGCACTTCG GCGGCGGCAACATCTTGGGAGTACCTCAGCAGCAACATGCCCAGCAAGA AGAATGGAAGAAGCGGACCCCAACCCCATAAAAGGTGGGTGTTCACTCT GAATAATCCTTCCGAAGACGAGCGCAAGAAAATACGGGATCTTCCAATA TCCCTATTTGATTATT**
Sequence_286	**AGCTGAAAGCGAAAGAAGTGCGCTGTAAGTATTACCAGCGCACTTCG GCAGCGGCAGCACCTCGGCAGCACCTCAGCAGCAACATGCCCAGCAAGA AGAATGGAAGAAGCGGACCCCAACCCCATAAAAGGTGGGTGTTCACTCT GAATAATCCTTCCGAAGACGAGCGCAAGAAAATACGGGATCTTCCAATA TCCCTATTTGATTATT**
Sequence_287	**GTCTGAAAACGAAAGAAGTGCGCTGTAAGTATTACCAGCGCACTTCG GCAGCGGCAGCACCTCGGCAGCACCTCAGCAGCAACATGCCCAGCAAGA AGAATGGAAGAAGCGGACCCCAACCCCATAAAAGGTGGGTGTTCACTCT GAATAATCCTTCCGAAGACGAGCGCAAGAAAATACGGGATCTTCCAATA TCCCTATTTGATTATT**
Sequence_288	**TTCTGAAAACGAAAGAAGTGCGCTGTAAGTATTACCAGCGCACTTCG GCAGCGGCAGCACCTCGGCAGCACCTCAGCAGCAACATGCCCAGCAAGA AGAATGGAAGAAGCGGACCCCAACCCCATAAAAGGTGGGTGTTCACTCT GAATAATCCTTCCGAAGACGAGCGCAAGAAAATACGGGATCTTCCAATA TCCCTATTTGATTATT**
Sequence_289	**ATCTGAAAACGAAAGAAGTGCGCTGTAAGTATTACCAGCGCACTTCG GCAGCGGCAGCACCTCGGCAGCACCTCAGCAGCAACATGCCTTCGAAGA AGAGCGGCAGGAGTGCCCCCCAACCCCATAAAAGGTGGGTGTTCACTCT GAATAATCCTTCCGAAGACGAGCGCAAGAAAATACGGGATCTTCCAATA TCCCTATTTGATTATT**
Sequence_290	**ATCTGAAAACGAAAGAAGTGCGCTGTAAGTATTACCAGCGCACTTCG GCAGCGGCAGCACCTCGGCAGCACCTCAGCAGCAACATGCCGTCTAAGA AAAACGGGAGATCGGTACCCCAACCCCATAAAAGGTGGGTGTTCACTCT GAATAATCCTTCCGAAGACGAGCGCAAGAAAATACGGGATCTTCCAATA TCCCTATTTGATTATT**
Sequence_291	**ATCTGAAAACGAAAGAAGTGCGCTGTAAGTATTACCAGCGCACTTCG GCAGCGGCAGCACCTCGGCAGCACCTCAGCAGCAACATGCCGAGCAAA AAAAACGGAAGGTCAGGTCCCCAACCCCATAAAAGGTGGGTGTTCACTC TGAATAATCCTTCCGAAGACGAGCGCAAGAAAATACGGGATCTTCCAAT**

	**ATCCCTATTTGATTATT**
Sequence_292	**ATCTGAAAACGAAAGAAGTGCGCTGTAAGTATTACCAGCGCACTTCGGCAGCGGCAGCACCTCGGCAGCACCTCAGCAGCAACATGCCCTCCAAGAAAAATGGGCGGAGTGGTCCCCAACCCCATAAAAGGTGGGTGTTCACTCTGAATAATCCTTCCGAAGACGAGCGCAAGAAAATACGGGATCTTCCAATATCCCTATTTGATTATT**
Sequence_293	**ATCTGAAAACGAAAGAAGTGCGCTGTAAGTATTACCAGCGCACTTCGGCAGCGGCAGCACCTCGGCAGCACCTCAGCAGCAACATGCCCAACAAAAAGAATGGCAAGTCGGGCCCCCAACCCCATAAAAGGTGGGTGTTCACTCTGAATAATCCTTCCGAAGACGAGCGCAAGAAAATACGGGATCTTCCAATATCCCTATTTGATTATT**
Sequence_294	**ATCTGAAAACGAAAGAAGTGCGCTGTAAGTATTACCAGCGCACTTCGGCAGCGGCAGCACCTCGGCAGCACCTCAGCAGCAACATGCCGGGCAAAAAAAATGGAAGGTCAGGCCCCCAACCCCATAAAAGGTGGGTGTTCACTCTGAATAATCCTTCCGAAGACGAGCGCAAGAAAATACGGGATCTTCCAATATCCCTATTTGATTATT**
Sequence_295	**ATCTGAAAACGAAAGAAGTGCGCTGTAAGTATTACCAGCGCACTTCGGCAGCGGCAGCACCTCGGCAGCACCTCAGCAGCAACATGCCGAACAAGAAAAATGGTCGGTCAGGACCCCAACCCCATAAAAGGTGGGTGTTCACTCTGAATAATCCTTCCGAAGACGAGCGCAAGAAAATACGGGATCTTCCAATATCCCTATTTGATTATT**
Sequence_296	**ATCTGAAAACGAAAGAAGTGCGCTGTAAGTATTACCAGCGCACTTCGGCAGCGGCAGCACCTCGGCAGCACCTCAGCAGCAACATGCCGGGCAAGAAGAGTGGAAGGTCGGGCCCCCAACCCCATAAAAGGTGGGTGTTCACTCTGAATAATCCTTCCGAAGACGAGCGCAAGAAAATACGGGATCTTCCAATATCCCTATTTGATTATT**
Sequence_297	**ATCTGAAAACGAAAGAAGTGCGCTGTAAGTATTACCAGCGCACTTCGGCAGCGGCAGCACCTCGGCAGCACCTCAGCAGCAACATGCCCCGACGTAAGAATGGTACGTCGGGGCCCCAACCCCATAAAAGGTGGGTGTTCACTCTGAATAATCCTTCCGAAGACGAGCGCAAGAAAATACGGGATCTTCCAATATCCCTATTTGATTATT**
Sequence_298	**ATCTGAAAACGAAAGAAGTGCGCTGTAAGTATTACCAGCGCACTTCGGCAGCGGCAGCACCTCGGCAGCACCTCAGCAGCAACATGCCCAGCAAGAAAAATGGGCGGTCGGGCCCCCAACCCCATAAAAGGTGGGTGTTCACTCTGAATAATCCTTCCGAAGACGAGCGCAAGAAAATACGGGATCTTCCAATATCCCTATTTGATTATT**
Sequence_299	**ATCTGAAAACGAAAGAAGTGCGCTGTAAGTATTACCAGCGCACTTCGGCAGCGGCAGCACCTCGGCAGCACCTCAGCAGCAACATGCCGTCCAAGAAAAATGGACGGTCAGGCCCCCAACCCCATAAAAGGTGGGTGTTCACTCTGAATAATCCTTCCGAAGACGAGCGCAAGAAAATACGGGATCTTCCAATATCCCTATTTGATTATT**
Sequence_300	**ATCTGAAAACGAAAGAAGTGCGCTGTAAGTATTACCAGCGCACTTCG**

	**GCAGCGGCAGCACCTCGGCAGCACCTCAGCAGCAACATGCCGTCAAAAA AATCTGGACGGAGCGGTCCCCAACCCCATAAAAGGTGGGTGTTCACTCT GAATAATCCTTCCGAAGACGAGCGCAAGAAAATACGGGATCTTCCAATA TCCCTATTTGATTATT**
Sequence_301	**ATCTGAAAACGAAAGAAGTGCGCTGTAAGTATTACCAGCGCACTTCG GCAGCGGCAGCACCTCGGCAGCACCTCAGCAGCAACATGCCCAGTAAAA AGTCTGGGCGCTCCGGCCCCAACCCCATAAAAGGTGGGTGTTCACTCTG AATAATCCTTCCGAAGACGAGCGCAAGAAAATACGGGATCTTCCAATAT CCCTATTTGATTATT**
Sequence_302	**ATCTGAAAACGAAAGAAGTGCGCTGTAAGTATTACCAGCGCACTTCG GCAGCGGCAGCACCTCGGCAGCACCTCAGCAGCAACATGCTGAGCAAG AAATCCGGTCGGTCCGGTCCCCAACCCCATAAAAGGTGGGTGTTCACTCT GAATAATCCTTCCGAAGACGAGCGCAAGAAAATACGGGATCTTCCAATA TCCCTATTTGATTATT**
Sequence_303	**ATCTGAAAACGAAAGAAGTGCGCTGTAAGTATTACCAGCGCACTTCG GCAGCGGCAGCACCTCGGCAGCACCTCAGCAGCAACATGCCGTCCAAGA AAAATGGACGGTCCGGACCCCAACCCCATAAAAGGTGGGTGTTCACTCT GAATAATCCTTCCGAAGACGAGCGCAAGAAAATACGGGATCTTCCAATA TCCCTATTTGATTATT**
Sequence_304	**ATCTGAAAACGAAAGAAGTGCGCTGTAAGTATTACCAGCGCACTTCG GCAGCGGCAGCACCTCGGCAGCACCTCAGCAGCAACATGCCCAGTAAGA AGTCTGGGCCCCAGCCACCCCAACCCCATAAAAGGTGGGTGTTCACTCTG AATAATCCTTCCGAAGACGAGCGCAAGAAAATACGGGATCTTCCAATAT CCCTATTTGATTATT**
Sequence_305	**ATCTGAAAACGAAAGAAGTGCGCTGTAAGTATTACCAGCGCACTTCG GCAGCGGCAGCACCTCGGCAGCACCTCAGCAGCAACATGCCTAATAAAA AGAACGGGCGTTCTGGCCCCAACCCCATAAAAGGTGGGTGTTCACTCT GAATAATCCTTCCGAAGACGAGCGCAAGAAAATACGGGATCTTCCAATA TCCCTATTTGATTATT**
Sequence_306	**ATCTGAAAACGAAAGAAGTGCGCTGTAAGTATTACCAGCGCACTTCG GCAGCGGCAGCACCTCGGCAGCACCTCAGCAGCAACATGCCGTCCAAGA AGAACGGCCGAAGCGGTCCCCAACCCCATAAAAGGTGGGTGTTCACTCT GAATAATCCTTCCGAAGACGAGCGCAAGAAAATACGGGATCTTCCAATA TCCCTATTTGATTATT**
Sequence_307	**ATCTGAAAACGAAAGAAGTGCGCTGTAAGTATTACCAGCGCACTTCG GCAGCGGCAGCACCTCGGCAGCACCTCAGCAGCAACATGCCGTCCAAAA AAAATGGACGGTCCGGGCCCCAACCCCATAAAAGGTGGGTGTTCACTCT GAATAATCCTTCCGAAGACGAGCGCAAGAAAATACGGGATCTTCCAATA TCCCTATTTGATTATT**
Sequence_308	**ATCTGAAAACGAAAGAAGTGCGCTGTAAGTATTACCAGCGCACTTCG GCAGCGGCAGCACCTCGGCAGCACCTCAGCAGCAACATGCCGTCAAAAA AAAACGGCATTTCAGGTCCCCAACCCCATAAAAGGTGGGTGTTCACTCTG AATAATCCTTCCGAAGACGAGCGCAAGAAAATACGGGATCTTCCAATAT**

	**CCCTATTTGATTATT**
Sequence_309	**ATCTGAAAACGAAAGAAGTGCGCTGTAAGTATTACCAGCGCACTTCG GCAGCGGCAGCACCTCGGCAGCACCTCAGCAGCAACATGCCCAGCAAAA AGAATGGGCGTTCAGGGCCCCAACCCCATAAAAGGTGGGTGTTCACTCT GAATAATCCTTCCGAAGACGAGCGCAAGAAAATACGGGATCTTCCAATA TCCCTATTTGATTATT**
Sequence_310	**ATCTGAAAACGAAAGAAGTGCGCTGTAAGTATTACCAGCGCACTTCG GCAGCGGCAGCACCTCGGCAGCACCTCAGCAGCAACATGCCATCAAAGA AAAACGGCCGGTCGGGCCCCCAACCCCATAAAAGGTGGGTGTTCACTCT GAATAATCCTTCCGAAGACGAGCGCAAGAAAATACGGGATCTTCCAATA TCCCTATTTGATTATT**
Sequence_311	**ATCTGAAAACGAAAGAAGTGCGCTGTAAGTATTACCAGCGCACTTCG GCAGCGGCAGCACCTCGGCAGCACCTCAGCAGCAACATGCCCAGTAAAA AATCAGGAAAATCTGGCCCCCAACCCCATAAAAGGTGGGTGTTCACTCT GAATAATCCTTCCGAAGACGAGCGCAAGAAAATACGGGATCTTCCAATA TCCCTATTTGATTATT**
Sequence_312	**ATCTGAAAACGAAAGAAGTGCGCTGTAAGTATTACCAGCGCACTTCG GCAGCGGCAGCACCTCGGCAGCACCTCAGCAGCAACATGCCGTCAAAGA GAAATGGCAGATCGGGCCCCCAACCCCATAAAAGGTGGGTGTTCACTCT GAATAATCCTTCCGAAGACGAGCGCAAGAAAATACGGGATCTTCCAATA TCCCTATTTGATTATT**
Sequence_313	**ATCTGAAAACGAAAGAAGTGCGCTGTAAGTATTACCAGCGCACTTCG GCAGCGGCAGCACCTCGGCAGCACCTCAGCAGCAACATGCCAAGTAAGA AGAACGGAAGATCTGGCCCCCAACCCCATAAAAGGTGGGTGTTCACTCT GAATAATCCTTCCGAAGACGAGCGCAAGAAAATACGGGATCTTCCAATA TCCCTATTTGATTATT**
Sequence_314	**ATCTGAAAACGAAAGAAGTGCGCTGTAAGTATTACCAGCGCACTTCG GCAGCGGCAGCACCTCGGCAGCACCTCAGCAGCAACATGCCGAGCAAA AAAAATAATCGCTCGGGACCCCAACCCCATAAAAGGTGGGTGTTCACTC TGAATAATCCTTCCGAAGACGAGCGCAAGAAAATACGGGATCTTCCAAT ATCCCTATTTGATTATT**
Sequence_315	**ATCTGAAAACGAAAGAAGTGCGCTGTAAGTATTACCAGCGCACTTCG GCAGCGGCAGCACCTCGGCAGCACCTCAGCAGCAACATGCCCAGCAAGA AGAATGGAAGAAGCGGACCCCAACCGCATAAGCGGTGGGTATTTACTCT GAATAATCCTTCCGAAGACGAGCGCAAGAAAATACGGGATCTTCCAATA TCCCTATTTGATTATT**
Sequence_316	**ATCTGAAAACGAAAGAAGTGCGCTGTAAGTATTACCAGCGCACTTCG GCAGCGGCAGCACCTCGGCAGCACCTCAGCAGCAACATGCCCAGCAAGA AGAATGGAAGAAGCGGACCCCAGCCGCATAAACGGTGGGTATTTACAC TGAATAATCCTTCCGAAGACGAGCGCAAGAAAATACGGGATCTTCCAAT ATCCCTATTTGATTATT**
Sequence_317	**ATCTGAAAACGAAAGAAGTGCGCTGTAAGTATTACCAGCGCACTTCG**

	**GCAGCGGCAGCACCTCGGCAGCACCTCAGCAGCAACATGCCCAGCAAGAAGAATGGAAGAAGCGGACCACAGCCCCACCGGCGGTGGGTTTTTACTCTGAATAATCCTTCCGAAGACGAGCGCAAGAAAATACGGGATCTTCCAATATCCCTATTTGATTATT**
Sequence_318	**ATCTGAAAACGAAAGAAGTGCGCTGTAAGTATTACCAGCGCACTTCGGCAGCGGCAGCACCTCGGCAGCACCTCAGCAGCAACATGCCCAGCAAGAAGAATGGAAGAAGCGGACCGCAGCCCCACAAAAGGTGGGTGCTGACGCTGAATAATCCTTCCGAAGACGAGCGCAAGAAAATACGGGATCTTCCAATATCCCTATTTGATTATT**
Sequence_319	**ATCTGAAAACGAAAGAAGTGCGCTGTAAGTATTACCAGCGCACTTCGGCAGCGGCAGCACCTCGGCAGCACCTCAGCAGCAACATGCCCAGCAAGAAGAATGGAAGAAGCGGACACAAGCGATGGGTTTTCACGCTCAATAATCTGAATAATCCTTCCGAAGACGAGCGCAAGAAAATACGGGATCTTCCAATATCCCTATTTGATTATT**
Sequence_320	**ATCTGAAAACGAAAGAAGTGCGCTGTAAGTATTACCAGCGCACTTCGGCAGCGGCAGCACCTCGGCAGCACCTCAGCAGCAACATGCCCAGCAAGAAGAATGGAAGAAGCGGACCTCAGCCCCATAAGCGGTGGGTCTTCACCCTGAATAATCCTTCCGAAGACGAGCGCAAGAAAATACGGGATCTTCCAATATCCCTATTTGATTATT**
Sequence_321	**ATCTGAAAACGAAAGAAGTGCGCTGTAAGTATTACCAGCGCACTTCGGCAGCGGCAGCACCTCGGCAGCACCTCAGCAGCAACATGCCCAGCAAGAAGAATGGAAGAAGCGGACCACAGCCACATAAGAGGTGGGTGTTTACACTGAATAATCCTTCCGAAGACGAGCGCAAGAAAATACGGGATCTTCCAATATCCCTATTTGATTATT**
Sequence_322	**ATCTGAAAACGAAAGAAGTGCGCTGTAAGTATTACCAGCGCACTTCGGCAGCGGCAGCACCTCGGCAGCACCTCAGCAGCAACATGCCCAGCAAGAAGAATGGAAGAAGCGGACCTCAGCCTCATAAACGATGGGTGTTTACCCTGAATAATCCTTCCGAAGACGAGCGCAAGAAAATACGGGATCTTCCAATATCCCTATTTGATTATT**
Sequence_323	**ATCTGAAAACGAAAGAAGTGCGCTGTAAGTATTACCAGCGCACTTCGGCAGCGGCAGCACCTCGGCAGCACCTCAGCAGCAACATGCCCAGCAAGAAGAATGGAAGAAGCGGACCCCAACCCCATAAGAAGTGGGTTTTTACCCTGAATAATCCTTCCGAAGACGAGCGCAAGAAAATACGGGATCTTCCAATATCCCTATTTGATTATT**
Sequence_324	**ATCTGAAAACGAAAGAAGTGCGCTGTAAGTATTACCAGCGCACTTCGGCAGCGGCAGCACCTCGGCAGCACCTCAGCAGCAACATGCCCAGCAAGAAGAATGGAAGAAGCGGACCACACCCACATAAGAGGTGGGTCTTTACCCTGAATAATCCTTCCGAAGACGAGCGCAAGAAAATACGGGATCTTCCAATATCCCTATTTGATTATT**
Sequence_325	**ATCTGAAAACGAAAGAAGTGCGCTGTAAGTATTACCAGCGCACTTCGGCAGCGGCAGCACCTCGGCAGCACCTCAGCAGCAACATGCCCAGCAAGAAGAATGGAAGAAGCGGACCGCAACCCCCTAAGCGCTGGGTCTTTACACTGAATAATCCTTCCGAAGACGAGCGCAAGAAAATACGGGATCTTCCAATA**

	**TCCCTATTTGATTATT**
Sequence_326	**ATCTGAAAACGAAAGAAGTGCGCTGTAAGTATTACCAGCGCACTTCG GCAGCGGCAGCACCTCGGCAGCACCTCAGCAGCAACATGCCCAGCAAGA AGAATGGAAGAAGCGGACCGCAGCCACACAAACGGTGGGTCTTCACCC TGAATAATCCTTCCGAAGACGAGCGCAAGAAAATACGGGATCTTCCAAT ATCCCTATTTGATTATT**
Sequence_327	**ATCTGAAAACGAAAGAAGTGCGCTGTAAGTATTACCAGCGCACTTCG GCAGCGGCAGCACCTCGGCAGCACCTCAGCAGCAACATGCCCAGCAAGA AGAATGGAAGAAGCGGACCTCAGCCACATAAGCGCTGGGTATTTACTCT GAATAATCCTTCCGAAGACGAGCGCAAGAAAATACGGGATCTTCCAATA TCCCTATTTGATTATT**
Sequence_328	**ATCTGAAAACGAAAGAAGTGCGCTGTAAGTATTACCAGCGCACTTCG GCAGCGGCAGCACCTCGGCAGCACCTCAGCAGCAACATGCCCAGCAAGA AGAATGGAAGAAGCGGACCCCAACCACACAAGAGATGGGTATTCACCC TGAATAATCCTTCCGAAGACGAGCGCAAGAAAATACGGGATCTTCCAAT ATCCCTATTTGATTATT**
Sequence_329	**ATCTGAAAACGAAAGAAGTGCGCTGTAAGTATTACCAGCGCACTTCG GCAGCGGCAGCACCTCGGCAGCACCTCAGCAGCAACATGCCCAGCAAGA AGAATGGAAGAAGCGGACCTCAACCGCATCGGAGATGGGTCTTTACTCT GAATAATCCTTCCGAAGACGAGCGCAAGAAAATACGGGATCTTCCAATA TCCCTATTTGATTATT**
Sequence_330	**ATCTGAAAACGAAAGAAGTGCGCTGTAAGTATTACCAGCGCACTTCG GCAGCGGCAGCACCTCGGCAGCACCTCAGCAGCAACATGCCCAGCAAGA AGAATGGAAGAAGCGGACCTCAACCACATAAGCGCTGGGTGTTTACGCT GAATAATCCTTCCGAAGACGAGCGCAAGAAAATACGGGATCTTCCAATA TCCCTATTTGATTATT**
Sequence_331	**ATCTGAAAACGAAAGAAGTGCGCTGTAAGTATTACCAGCGCACTTCG GCAGCGGCAGCACCTCGGCAGCACCTCAGCAGCAACATGCCCAGCAAGA AGAATGGAAGAAGCGGACCGCAGCCACACAAACGTTGGGTGTTCACAC TGAATAATCCTTCCGAAGACGAGCGCAAGAAAATACGGGATCTTCCAAT ATCCCTATTTGATTATT**
Sequence_332	**ATCTGAAAACGAAAGAAGTGCGCTGTAAGTATTACCAGCGCACTTCG GCAGCGGCAGCACCTCGGCAGCACCTCAGCAGCAACATGCCCAGCAAGA AGAATGGAAGAAGCGGACCCCAACCGCACAAGCGGTGGGTGTTCACCC TGAATAATCCTTCCGAAGACGAGCGCAAGAAAATACGGGATCTTCCAAT ATCCCTATTTGATTATT**
Sequence_333	**ATCTGAAAACGAAAGAAGTGCGCTGTAAGTATTACCAGCGCACTTCG GCAGCGGCAGCACCTCGGCAGCACCTCAGCAGCAACATGCCCAGCAAGA AGAATGGAAGAAGCGGACCCCAACCCCACAAACGCTGGGTCTTCACGCT GAATAATCCTTCCGAAGACGAGCGCAAGAAAATACGGGATCTTCCAATA TCCCTATTTGATTATT**
Sequence_334	**ATCTGAAAACGAAAGAAGTGCGCTGTAAGTATTACCAGCGCACTTCG**

	**GCAGCGGCAGCACCTCGGCAGCACCTCAGCAGCAACATGCCCAGCAAGA AGAATGGAAGAAGCGGACCCCAGCCGCACAAGCGGTGGGTCTTCACCCT GAATAATCCTTCCGAAGACGAGCGCAAGAAAATACGGGATCTTCCAATA TCCCTATTTGATTATT**
Sequence_335	**ATCTGAAAACGAAAGAAGTGCGCTGTAAGTATTACCAGCGCACTTCG GCAGCGGCAGCACCTCGGCAGCACCTCAGCAGCAACATGCCCAGCAAGA AGAATGGAAGAAGCGGACCCCAACCCCATAAAAGGTGGGTGTTCACGT TGAATAACCCATCAGAGGACGAGCGGAAGAAAATACGGGATCTTCCAA TATCCCTATTTGATTATT**
Sequence_336	**ATCTGAAAACGAAAGAAGTGCGCTGTAAGTATTACCAGCGCACTTCG GCAGCGGCAGCACCTCGGCAGCACCTCAGCAGCAACATGCCCAGCAAGA AGAATGGAAGAAGCGGACCCCAACCCCATAAAAGGTGGGTGTTCACGC TTAACAACTCAAGTGAGGATGAGCGCAAGAAAATACGGGATCTTCCAAT ATCCCTATTTGATTATT**
Sequence_337	**ATCTGAAAACGAAAGAAGTGCGCTGTAAGTATTACCAGCGCACTTCG GCAGCGGCAGCACCTCGGCAGCACCTCAGCAGCAACATGCCCAGCAAGA AGAATGGAAGAAGCGGACCCCAACCCCATAAAAGGTGGGTGTTCACTTT GAATAATCCGAGCGAGGACGAACGGAAGAAAATACGGGATCTTCCAAT ATCCCTATTTGATTATT**
Sequence_338	**ATCTGAAAACGAAAGAAGTGCGCTGTAAGTATTACCAGCGCACTTCG GCAGCGGCAGCACCTCGGCAGCACCTCAGCAGCAACATGCCCAGCAAGA AGAATGGAAGAAGCGGACCCCAACCCCATAAAAGGTGGGTGTTCACGC TCAATAACCCGAGCGAGGACGAACGCAAGAAAATACGGGATCTTCCAAT ATCCCTATTTGATTATT**
Sequence_339	**ATCTGAAAACGAAAGAAGTGCGCTGTAAGTATTACCAGCGCACTTCG GCAGCGGCAGCACCTCGGCAGCACCTCAGCAGCAACATGCCCAGCAAGA AGAATGGAAGAAGCGGACCCCAACCCCATAAAAGGTGGGTGTTCACTCT GAATAACCCGTCAGAGGACGAAAGGAAGAAAATACGGGATCTTCCAAT ATCCCTATTTGATTATT**
Sequence_340	**ATCTGAAAACGAAAGAAGTGCGCTGTAAGTATTACCAGCGCACTTCG GCAGCGGCAGCACCTCGGCAGCACCTCAGCAGCAACATGCCCAGCAAGA AGAATGGAAGAAGCGGACCCCAACCCCATAAAAGGTGGGTGTTCACGC TCAACAATCCGAGTGAGGATGAGCGTAAGAAAATACGGGATCTTCCAAT ATCCCTATTTGATTATT**
Sequence_341	**ATCTGAAAACGAAAGAAGTGCGCTGTAAGTATTACCAGCGCACTTCG GCAGCGGCAGCACCTCGGCAGCACCTCAGCAGCAACATGCCCAGCAAGA AGAATGGAAGAAGCGGACCCCAACCCCATAAAAGGTGGGTGTTCACGC TCTCCAATCCGAGTGAGGATGAGCGCAAGAAAATACGGGATCTTCCAAT ATCCCTATTTGATTATT**
Sequence_342	**ATCTGAAAACGAAAGAAGTGCGCTGTAAGTATTACCAGCGCACTTCG GCAGCGGCAGCACCTCGGCAGCACCTCAGCAGCAACATGCCCAGCAAGA AGAATGGAAGAAGCGGACCCCAACCCCATAAAAGGTGGGTGTTCACGC TGAATAATCCTTCCGAAGATGAGCGCAAGAAAATACGGGATCTTCCAAT**

	**ATCCCTATTTGATTATT**
Sequence_343	**ATCTGAAAACGAAAGAAGTGCGCTGTAAGTATTACCAGCGCACTTCG GCAGCGGCAGCACCTCGGCAGCACCTCAGCAGCAACATGCCCAGCAAGA AGAATGGAAGAAGCGGACCCCAACCCCATAAAAGGTGGGTGTTCACGC TCAATAATCCGTCAGAGGATGAGCGTAAGAAAATACGGGATCTTCCAAT ATCCCTATTTGATTATT**
Sequence_344	**ATCTGAAAACGAAAGAAGTGCGCTGTAAGTATTACCAGCGCACTTCG GCAGCGGCAGCACCTCGGCAGCACCTCAGCAGCAACATGCCCAGCAAGA AGAATGGAAGAAGCGGACCCCAACCCCATAAAAGGTGGGTGTTCACTCT CAATAACCCTTCCGAGGATGAGCGGAAGAAAATACGGGATCTTCCAATA TCCCTATTTGATTATT**
Sequence_345	**ATCTGAAAACGAAAGAAGTGCGCTGTAAGTATTACCAGCGCACTTCG GCAGCGGCAGCACCTCGGCAGCACCTCAGCAGCAACATGCCCAGCAAGA AGAATGGAAGAAGCGGACCCCAACCCCATAAAAGGTGGGTGTTCACTCT CAATAACCCCTCTGAGGAGGAAAAGAAGAAAATACGGGATCTTCCAATA TCCCTATTTGATTATT**
Sequence_346	**ATCTGAAAACGAAAGAAGTGCGCTGTAAGTATTACCAGCGCACTTCG GCAGCGGCAGCACCTCGGCAGCACCTCAGCAGCAACATGCCCAGCAAGA AGAATGGAAGAAGCGGACCCCAACCCCATAAAAGGTGGGTGTTCACGT TGAATAATCCCTCAGAAGATGAGCGAAAGAAAATACGGGATCTTCCAAT ATCCCTATTTGATTATT**
Sequence_347	**ATCTGAAAACGAAAGAAGTGCGCTGTAAGTATTACCAGCGCACTTCG GCAGCGGCAGCACCTCGGCAGCACCTCAGCAGCAACATGCCCAGCAAGA AGAATGGAAGAAGCGGACCCCAACCCCATAAAAGGTGGGTGTTCACGC TCAACAACCCTTCGGAGGATGAGCGCAAGAAAATACGGGATCTTCCAAT ATCCCTATTTGATTATT**
Sequence_348	**ATCTGAAAACGAAAGAAGTGCGCTGTAAGTATTACCAGCGCACTTCG GCAGCGGCAGCACCTCGGCAGCACCTCAGCAGCAACATGCCCAGCAAGA AGAATGGAAGAAGCGGACCCCAACCCCATAAAAGGTGGGTGTTCACGC TCAATAATCCTAGTGAGGATGAGCGTAAGAAAATACGGGATCTTCCAAT ATCCCTATTTGATTATT**
Sequence_349	**ATCTGAAAACGAAAGAAGTGCGCTGTAAGTATTACCAGCGCACTTCG GCAGCGGCAGCACCTCGGCAGCACCTCAGCAGCAACATGCCCAGCAAGA AGAATGGAAGAAGCGGACCCCAACCCCATAAAAGGTGGGTGTTCACCCT TAATGATCCGTCGGAGGATGAGCGGAAGAAAATACGGGATCTTCCAAT ATCCCTATTTGATTATT**
Sequence_350	**ATCTGAAAACGAAAGAAGTGCGCTGTAAGTATTACCAGCGCACTTCG GCAGCGGCAGCACCTCGGCAGCACCTCAGCAGCAACATGCCCAGCAAGA AGAATGGAAGAAGCGGACCCCAACCCCATAAAAGGTGGGTGTTCAATC CCTCTGAAGAGGAGAAGAACAAAATAAAGAAAATACGGGATCTTCCAA TATCCCTATTTGATTATT**
Sequence_351	**ATCTGAAAACGAAAGAAGTGCGCTGTAAGTATTACCAGCGCACTTCG**

	**GCAGCGGCAGCACCTCGGCAGCACCTCAGCAGCAACATGCCCAGCAAGAAGAATGGAAGAAGCGGACCCCAACCCCATAAAAGGTGGGTGTTCACGCTCAACAATCCCAGCGAGGATGAGCGTAAGAAAATACGGGATCTTCCAATATCCCTATTTGATTATT**
Sequence_352	**ATCTGAAAACGAAAGAAGTGCGCTGTAAGTATTACCAGCGCACTTCGGCAGCGGCAGCACCTCGGCAGCACCTCAGCAGCAACATGCCCAGCAAGAAGAATGGAAGAAGCGGACCCCAACCCCATAAAAGGTGGGTGTTCACCTTGAATAACCCCTCAGAGGATGAACGAAAGAAAATACGGGATCTTCCAATATCCCTATTTGATTATT**
Sequence_353	**ATCTGAAAACGAAAGAAGTGCGCTGTAAGTATTACCAGCGCACTTCGGCAGCGGCAGCACCTCGGCAGCACCTCAGCAGCAACATGCCCAGCAAGAAGAATGGAAGAAGCGGACCCCAACCCCATAAAAGGTGGGTGTTCACCTTGAACAACCCCTCGGAGAACGAGCGGAAGAAAATACGGGATCTTCCAATATCCCTATTTGATTATT**
Sequence_354	**ATCTGAAAACGAAAGAAGTGCGCTGTAAGTATTACCAGCGCACTTCGGCAGCGGCAGCACCTCGGCAGCACCTCAGCAGCAACATGCCCAGCAAGAAGAATGGAAGAAGCGGACCCCAACCCCATAAAAGGTGGGTGTTCACTCTCAATACGCCCAGCGAAGATGAGCGCAAGAAAATACGGGATCTTCCAATATCCCTATTTGATTATT**
Sequence_355	**ATCTGAAAACGAAAGAAGTGCGCTGTAAGTATTACCAGCGCACTTCGGCAGCGGCAGCACCTCGGCAGCACCTCAGCAGCAACATGCCCAGCAAGAAGAATGGAAGAAGCGGACCCCAACCCCATAAAAGGTGGGTGTTCACGCTCAATAATCCATCCGAAGCGGAGCGCAAGAAAATACGGGATCTTCCAATATCCCTATTTGATTATT**
Sequence_356	**ATCTGAAAACGAAAGAAGTGCGCTGTAAGTATTACCAGCGCACTTCGGCAGCGGCAGCACCTCGGCAGCACCTCAGCAGCAACATGCCCAGCAAGAAGAATGGAAGAAGCGGACCCCAACCCCATAAAAGGTGGGTGTTCACCCTTAACAACCCCTCCGAGGACGAGCGCAAGAAAATACGGGATCTTCCAATATCCCTATTTGATTATT**
Sequence_357	**ATCTGAAAACGAAAGAAGTGCGCTGTAAGTATTACCAGCGCACTTCGGCAGCGGCAGCACCTCGGCAGCACCTCAGCAGCAACATGCCCAGCAAGAAGAATGGAAGAAGCGGACCCCAACCCCATAAAAGGTGGGTGTTCACGCTCAATGATCCTAGCGAGGATGAGCGTAAGAAAATACGGGATCTTCCAATATCCCTATTTGATTATT**
Sequence_358	**ATCTGAAAACGAAAGAAGTGCGCTGTAAGTATTACCAGCGCACTTCGGCAGCGGCAGCACCTCGGCAGCACCTCAGCAGCAACATGCCCAGCAAGAAGAATGGAAGAAGCGGACCCCAACCCCATAAAAGGTGGGTGTTCACGCTCAACAACCCCTCGGAGGAGGAGCGTAAGAAAATACGGGATCTTCCAATATCCCTATTTGATTATT**
Sequence_359	**ATCTGAAAACGAAAGAAGTGCGCTGTAAGTATTACCAGCGCACTTCGGCAGCGGCAGCACCTCGGCAGCACCTCAGCAGCAACATGCCCAGCAAGAAGAATGGAAGAAGCGGACCCCAACCCCATAAAAGGTGGGTGTTCACCGTGAATAATCCTAGTGAGGATGAGCGGAAGAAAATACGGGATCTTCCAAT**

	**ATCCCTATTTGATTATT**
Sequence_360	**ATCTGAAAACGAAAGAAGTGCGCTGTAAGTATTACCAGCGCACTTCG GCAGCGGCAGCACCTCGGCAGCACCTCAGCAGCAACATGCCTTCGCGCA AGTCGGGACGTAGTGGCCCCCAACCCCATAAAAGGTGGGTGTTCACTCT GAATAATCCTTCCGAAGACGAGCGCAAGAAAATACGGGATCTTCCAATA TCCCTATTTGATTATT**
Sequence_361	**ATCTGAAAACGAAAGAAGTGCGCTGTAAGTATTACCAGCGCACTTCG GCAGCGGCAGCACCTCGGCAGCACCTCAGCAGCAACATGCCTTCACGCA AGAACGGGCGGTCCGGACCCCAACCCCATAAAAGGTGGGTGTTCACTCT GAATAATCCTTCCGAAGACGAGCGCAAGAAAATACGGGATCTTCCAATA TCCCTATTTGATTATT**
Sequence_362	**ATCTGAAAACGAAAGAAGTGCGCTGTAAGTATTACCAGCGCACTTCG GCAGCGGCAGCACCTCGGCAGCACCTCAGCAGCAACATGCCAAGTAAAA AGAATGGCCGATCTGGACCCCAACCCCATAAAAGGTGGGTGTTCACTCT GAATAATCCTTCCGAAGACGAGCGCAAGAAAATACGGGATCTTCCAATA TCCCTATTTGATTATT**
Sequence_363	**ATCTGAAAACGAAAGAAGTGCGCTGTAAGTATTACCAGCGCACTTCG GCAGCGGCAGCACCTCGGCAGCACCTCAGCAGCAACATGCCTTCCAAAA AGTCAGGGAAATCAGGTCCCCAACCCCATAAAAGGTGGGTGTTCACTCT GAATAATCCTTCCGAAGACGAGCGCAAGAAAATACGGGATCTTCCAATA TCCCTATTTGATTATT**
Sequence_364	**ATCTGAAAACGAAAGAAGTGCGCTGTAAGTATTACCAGCGCACTTCG GCAGCGGCAGCACCTCGGCAGCACCTCAGCAGCAACATGCCATCAAAAA AATCAGGACGAAGCGGACCCCAACCCCATAAAAGGTGGGTGTTCACTCT GAATAATCCTTCCGAAGACGAGCGCAAGAAAATACGGGATCTTCCAATA TCCCTATTTGATTATT**
Sequence_365	**ATCTGAAAACGAAAGAAGTGCGCTGTAAGTATTACCAGCGCACTTCG GCAGCGGCAGCACCTCGGCAGCACCTCAGCAGCAACATGCCATCGAAAA AATCAGGAGGATCAGGGCCCCAACCCCATAAAAGGTGGGTGTTCACTCT GAATAATCCTTCCGAAGACGAGCGCAAGAAAATACGGGATCTTCCAATA TCCCTATTTGATTATT**
Sequence_366	**ATCTGAAAACGAAAGAAGTGCGCTGTAAGTATTACCAGCGCACTTCG GCAGCGGCAGCACCTCGGCAGCACCTCAGCAGCAACATGCCGTCAAAAA AGAACGGGCGATGTGGCCCCCAACCCCATAAAAGGTGGGTGTTCACTCT GAATAATCCTTCCGAAGACGAGCGCAAGAAAATACGGGATCTTCCAATA TCCCTATTTGATTATT**
Sequence_367	**ATCTGAAAACGAAAGAAGTGCGCTGTAAGTATTACCAGCGCACTTCG GCAGCGGCAGCACCTCGGCAGCACCTCAGCAGCAACATGCCATCTAAGA AATCCGGACCTCAACCACCCCAACCCCATAAAAGGTGGGTGTTCACTCTG AATAATCCTTCCGAAGACGAGCGCAAGAAAATACGGGATCTTCCAATAT CCCTATTTGATTATT**
Sequence_368	**ATCTGAAAACGAAAGAAGTGCGCTGTAAGTATTACCAGCGCACTTCG**

	**GCAGCGGCAGCACCTCGGCAGCACCTCAGCAGCAACATGCCCTCCAAGAAACATGGTCGCAGCGGTCCCCAACCCCATAAAAGGTGGGTGTTCACTCTGAATAATCCTTCCGAAGACGAGCGCAAGAAAATACGGGATCTTCCAATATCCCTATTTGATTATT**
Sequence_369	**ATCTGAAAACGAAAGAAGTGCGCTGTAAGTATTACCAGCGCACTTCGGCAGCGGCAGCACCTCGGCAGCACCTCAGCAGCAACATGCCTTCGAAAAAAAGTGGAGGGTCAGGTCCCCAACCCCATAAAAGGTGGGTGTTCACTCTGAATAATCCTTCCGAAGACGAGCGCAAGAAAATACGGGATCTTCCAATATCCCTATTTGATTATT**
Sequence_370	**ATCTGAAAACGAAAGAAGTGCGCTGTAAGTATTACCAGCGCACTTCGGCAGCGGCAGCACCTCGGCAGCACCTCAGCAGCAACATGCCGAGCAGGAAAAGTGGTCCGCAACCGCCCCAACCCCATAAAAGGTGGGTGTTCACTCTGAATAATCCTTCCGAAGACGAGCGCAAGAAAATACGGGATCTTCCAATATCCCTATTTGATTATT**
Sequence_371	**ATCTGAAAACGAAAGAAGTGCGCTGTAAGTATTACCAGCGCACTTCGGCAGCGGCAGCACCTCGGCAGCACCTCAGCAGCAACATGCCCAACAAGAAGAACGGACGATCAGGTCCCCAACCCCATAAAAGGTGGGTGTTCACTCTGAATAATCCTTCCGAAGACGAGCGCAAGAAAATACGGGATCTTCCAATATCCCTATTTGATTATT**
Sequence_372	**ATCTGAAAACGAAAGAAGTGCGCTGTAAGTATTACCAGCGCACTTCGGCAGCGGCAGCACCTCGGCAGCACCTCAGCAGCAACATGTCGAGTAAGAAGTCTGGCCCACAGCCTCCCCAACCCCATAAAAGGTGGGTGTTCACTCTGAATAATCCTTCCGAAGACGAGCGCAAGAAAATACGGGATCTTCCAATATCCCTATTTGATTATT**
Sequence_373	**ATCTGAAAACGAAAGAAGTGCGCTGTAAGTATTACCAGCGCACTTCGGCAGCGGCAGCACCTCGGCAGCACCTCAGCAGCAACATGCCGAGTAAGAAAAATGGAAGAAGTGGACCCCAACCCCATAAAAGGTGGGTGTTCACTCTGAATAATCCTTCCGAAGACGAGCGCAAGAAAATACGGGATCTTCCAATATCCCTATTTGATTATT**
Sequence_374	**ATCTGAAAACGAAAGAAGTGCGCTGTAAGTATTACCAGCGCACTTCGGCAGCGGCAGCACCTCGGCAGCACCTCAGCAGCAACATGCCAGGTAAGAAGAGCGGGCGTTCGGGACCCCAACCCCATAAAAGGTGGGTGTTCACTCTGAATAATCCTTCCGAAGACGAGCGCAAGAAAATACGGGATCTTCCAATATCCCTATTTGATTATT**
Sequence_375	**ATCTGAAAACGAAAGAAGTGCGCTGTAAGTATTACCAGCGCACTTCGGCAGCGGCAGCACCTCGGCAGCACCTCAGCAGCAACATGCCATCCAAAAAAAATGAACGATCAGGGCCCCAACCCCATAAAAGGTGGGTGTTCACTCTGAATAATCCTTCCGAAGACGAGCGCAAGAAAATACGGGATCTTCCAATATCCCTATTTGATTATT**
Sequence_376	**ATCTGAAAACGAAAGAAGTGCGCTGTAAGTATTACCAGCGCACTTCGGCAGCGGCAGCACCTCGGCAGCACCTCAGCAGCAACATGCCATCCAAAAAGAACGGAAAATCAGGGCCCCAACCCCATAAAAGGTGGGTGTTCACTCTGAATAATCCTTCCGAAGACGAGCGCAAGAAAATACGGGATCTTCCAATA**

	**TCCCTATTTGATTATT**
Sequence_377	**ATCTGAAAACGAAAGAAGTGCGCTGTAAGTATTACCAGCGCACTTCG GCAGCGGCAGCACCTCGGCAGCACCTCAGCAGCAACATGCCGAGTAAG AAAAGTGGAGGATCAGGGCCCCAACCCCATAAAAGGTGGGTGTTCACTC TGAATAATCCTTCCGAAGACGAGCGCAAGAAAATACGGGATCTTCCAAT ATCCCTATTTGATTATT**
Sequence_378	**ATCTGAAAACGAAAGAAGTGCGCTGTAAGTATTACCAGCGCACTTCG GCAGCGGCAGCACCTCGGCAGCACCTCAGCAGCAACATGCCGAGTAAAA AGAATGGAAGATCAGTACCCCAACCCCATAAAAGGTGGGTGTTCACTCT GAATAATCCTTCCGAAGACGAGCGCAAGAAAATACGGGATCTTCCAATA TCCCTATTTGATTATT**
Sequence_379	**ATCTGAAAACGAAAGAAGTGCGCTGTAAGTATTACCAGCGCACTTCG GCAGCGGCAGCACCTCGGCAGCACCTCAGCAGCAACATGCCGAGCAAG AAGAACGAGCGGTCCGGTCCCCAACCCCATAAAAGGTGGGTGTTCACTC TGAATAATCCTTCCGAAGACGAGCGCAAGAAAATACGGGATCTTCCAAT ATCCCTATTTGATTATT**
Sequence_380	**ATCTGAAAACGAAAGAAGTGCGCTGTAAGTATTACCAGCGCACTTCG GCAGCGGCAGCACCTCGGCAGCACCTCAGCAGCAACATGCCCAGTAAGA AGAGCGGTAGGAGTGGACCCCAACCCCATAAAAGGTGGGTGTTCACTCT GAATAATCCTTCCGAAGACGAGCGCAAGAAAATACGGGATCTTCCAATA TCCCTATTTGATTATT**
Sequence_381	**ATCTGAAAACGAAAGAAGTGCGCTGTAAGTATTACCAGCGCACTTCG GCAGCGGCAGCACCTCGGCAGCACCTCAGCAGCAACATGCCGTCGAAAA AACACGGGCGCAGTGGTCCCCAACCCCATAAAAGGTGGGTGTTCACTCT GAATAATCCTTCCGAAGACGAGCGCAAGAAAATACGGGATCTTCCAATA TCCCTATTTGATTATT**
Sequence_382	**ATCTGAAAACGAAAGAAGTGCGCTGTAAGTATTACCAGCGCACTTCG GCAGCGGCAGCACCTCGGCAGCACCTCAGCAGCAACATGCCATCTAGAA AGAGCGGAAGAAGTGGACCCCAACCCCATAAAAGGTGGGTGTTCACTCT GAATAATCCTTCCGAAGACGAGCGCAAGAAAATACGGGATCTTCCAATA TCCCTATTTGATTATT**
Sequence_383	**ATCTGAAAACGAAAGAAGTGCGCTGTAAGTATTACCAGCGCACTTCG GCAGCGGCAGCACCTCGGCAGCACCTCAGCAGCAACATGTCTTCAAAAA AAAACGGCCGAAGCGGTCCCCAACCCCATAAAAGGTGGGTGTTCACTCT GAATAATCCTTCCGAAGACGAGCGCAAGAAAATACGGGATCTTCCAATA TCCCTATTTGATTATT**
Sequence_384	**ATCTGAAAACGAAAGAAGTGCGCTGTAAGTATTACCAGCGCACTTCG GCAGCGGCAGCACCTCGGCAGCACCTCAGCAGCAACATGCCACGGCGGA AGAACGGTACCTCTGGCCCCCAACCCCATAAAAGGTGGGTGTTCACTCTG AATAATCCTTCCGAAGACGAGCGCAAGAAAATACGGGATCTTCCAATAT CCCTATTTGATTATT**
Sequence_385	**ATCTGAAAACGAAAGAAGTGCGCTGTAAGTATTACCAGCGCACTTCG**

	**GCAGCGGCAGCACCTCGGCAGCACCTCAGCAGCAACATGCCTTCTCGGA AAAATGGACGTAGCGGACCCCAACCCCATAAAAGGTGGGTGTTCACTCT GAATAATCCTTCCGAAGACGAGCGCAAGAAAATACGGGATCTTCCAATA TCCCTATTTGATTATT**
Sequence_386	**ATCTGAAAACGAAAGAAGTGCGCTGTAAGTATTACCAGCGCACTTCG GCAGCGGCAGCACCTCGGCAGCACCTCAGCAGCAACATGCCCAGCAAGA AGAATGGAAGAAGCGGACCCCAGCCTCACAAGAAATGGGTGTTTACACT GAATAATCCTTCCGAAGACGAGCGCAAGAAAATACGGGATCTTCCAATA TCCCTATTTGATTATT**
Sequence_387	**ATCTGAAAACGAAAGAAGTGCGCTGTAAGTATTACCAGCGCACTTCG GCAGCGGCAGCACCTCGGCAGCACCTCAGCAGCAACATGCCCAGCAAGA AGAATGGAAGAAGCGGACCTCAGCCGCACAAGAGATGGGTTTTTACTCT GAATAATCCTTCCGAAGACGAGCGCAAGAAAATACGGGATCTTCCAATA TCCCTATTTGATTATT**
Sequence_388	**ATCTGAAAACGAAAGAAGTGCGCTGTAAGTATTACCAGCGCACTTCG GCAGCGGCAGCACCTCGGCAGCACCTCAGCAGCAACATGCCCAGCAAGA AGAATGGAAGAAGCGGACCACAACCTCATAAGCGCTGGGTCTTTACCCT GAATAATCCTTCCGAAGACGAGCGCAAGAAAATACGGGATCTTCCAATA TCCCTATTTGATTATT**
Sequence_389	**ATCTGAAAACGAAAGAAGTGCGCTGTAAGTATTACCAGCGCACTTCG GCAGCGGCAGCACCTCGGCAGCACCTCAGCAGCAACATGCCCAGCAAGA AGAATGGAAGAAGCGGACCGCAGCCGCATAAACGTTGGGTCCTGACAC TGAATAATCCTTCCGAAGACGAGCGCAAGAAAATACGGGATCTTCCAAT ATCCCTATTTGATTATT**
Sequence_390	**ATCTGAAAACGAAAGAAGTGCGCTGTAAGTATTACCAGCGCACTTCG GCAGCGGCAGCACCTCGGCAGCACCTCAGCAGCAACATGCCCAGCAAGA AGAATGGAAGAAGCGGACCGCATCCTCATAAACGCTGGGTCTTTACACT GAATAATCCTTCCGAAGACGAGCGCAAGAAAATACGGGATCTTCCAATA TCCCTATTTGATTATT**
Sequence_391	**ATCTGAAAACGAAAGAAGTGCGCTGTAAGTATTACCAGCGCACTTCG GCAGCGGCAGCACCTCGGCAGCACCTCAGCAGCAACATGCCCAGCAAGA AGAATGGAAGAAGCGGACCTCAGCCACATAAGAAATGGGTGTTTACGC TGAATAATCCTTCCGAAGACGAGCGCAAGAAAATACGGGATCTTCCAAT ATCCCTATTTGATTATT**
Sequence_392	**ATCTGAAAACGAAAGAAGTGCGCTGTAAGTATTACCAGCGCACTTCG GCAGCGGCAGCACCTCGGCAGCACCTCAGCAGCAACATGCCCAGCAAGA AGAATGGAAGAAGCGGACCGCAGCCTCATAAAAGGTGGGTCTTTACCCT GAATAATCCTTCCGAAGACGAGCGCAAGAAAATACGGGATCTTCCAATA TCCCTATTTGATTATT**
Sequence_393	**ATCTGAAAACGAAAGAAGTGCGCTGTAAGTATTACCAGCGCACTTCG GCAGCGGCAGCACCTCGGCAGCACCTCAGCAGCAACATGCCCAGCAAGA AGAATGGAAGAAGCGGACCGCAACCCCACAAAAGGTGGGTATTTACGC TGAATAATCCTTCCGAAGACGAGCGCAAGAAAATACGGGATCTTCCAAT**

	**ATCCCTATTTGATTATT**
Sequence_394	**ATCTGAAAACGAAAGAAGTGCGCTGTAAGTATTACCAGCGCACTTCG GCAGCGGCAGCACCTCGGCAGCACCTCAGCAGCAACATGCCCAGCAAGA AGAATGGAAGAAGCGGACCCCAACCGCACAAAAGATGGGTCTTTACGC TGAATAATCCTTCCGAAGACGAGCGCAAGAAAATACGGGATCTTCCAAT ATCCCTATTTGATTATT**
Sequence_395	**ATCTGAAAACGAAAGAAGTGCGCTGTAAGTATTACCAGCGCACTTCG GCAGCGGCAGCACCTCGGCAGCACCTCAGCAGCAACATGCCCAGCAAGA AGAATGGAAGAAGCGGACCACAACCTCATAAGCGCTGGGTCTTCACGCT GAATAATCCTTCCGAAGACGAGCGCAAGAAAATACGGGATCTTCCAATA TCCCTATTTGATTATT**
Sequence_396	**ATCTGAAAACGAAAGAAGTGCGCTGTAAGTATTACCAGCGCACTTCG GCAGCGGCAGCACCTCGGCAGCACCTCAGCAGCAACATGCCCAGCAAGA AGAATGGAAGAAGCGGACCACAACCACACAAACGCTGGGTCTTCACACT GAATAATCCTTCCGAAGACGAGCGCAAGAAAATACGGGATCTTCCAATA TCCCTATTTGATTATT**
Sequence_397	**ATCTGAAAACGAAAGAAGTGCGCTGTAAGTATTACCAGCGCACTTCG GCAGCGGCAGCACCTCGGCAGCACCTCAGCAGCAACATGCCCAGCAAGA AGAATGGAAGAAGCGGACCACACCCCCACAAGAGATGGGTCTTCACACT GAATAATCCTTCCGAAGACGAGCGCAAGAAAATACGGGATCTTCCAATA TCCCTATTTGATTATT**
Sequence_398	**ATCTGAAAACGAAAGAAGTGCGCTGTAAGTATTACCAGCGCACTTCG GCAGCGGCAGCACCTCGGCAGCACCTCAGCAGCAACATGCCCAGCAAGA AGAATGGAAGAAGCGGACCACAACCATACAAACGCTGGGTATTTACACT GAATAATCCTTCCGAAGACGAGCGCAAGAAAATACGGGATCTTCCAATA TCCCTATTTGATTATT**
Sequence_399	**ATCTGAAAACGAAAGAAGTGCGCTGTAAGTATTACCAGCGCACTTCG GCAGCGGCAGCACCTCGGCAGCACCTCAGCAGCAACATGCCCAGCAAGA AGAATGGAAGAAGCGGACATAAACGCTGGGTCTTTACTCTAAACAATCT GAATAATCCTTCCGAAGACGAGCGCAAGAAAATACGGGATCTTCCAATA TCCCTATTTGATTATT**
Sequence_400	**ATCTGAAAACGAAAGAAGTGCGCTGTAAGTATTACCAGCGCACTTCG GCAGCGGCAGCACCTCGGCAGCACCTCAGCAGCAACATGCCCAGCAAGA AGAATGGAAGAAGCGGACCCCAACCTCATAAGCGCTGGGTTTTTACTCT GAATAATCCTTCCGAAGACGAGCGCAAGAAAATACGGGATCTTCCAATA TCCCTATTTGATTATT**
Sequence_401	**ATCTGAAAACGAAAGAAGTGCGCTGTAAGTATTACCAGCGCACTTCG GCAGCGGCAGCACCTCGGCAGCACCTCAGCAGCAACATGCCCAGCAAGA AGAATGGAAGAAGCGGACCGCAGCCATATAAGCGTTGGGTCTTTACCCT GAATAATCCTTCCGAAGACGAGCGCAAGAAAATACGGGATCTTCCAATA TCCCTATTTGATTATT**
Sequence_402	**ATCTGAAAACGAAAGAAGTGCGCTGTAAGTATTACCAGCGCACTTCG**

	**GCAGCGGCAGCACCTCGGCAGCACCTCAGCAGCAACATGCCCAGCAAGAAGAATGGAAGAAGCGGACCACAACCTCATAAACGCTGGGCTTTTACCCTGAATAATCCTTCCGAAGACGAGCGCAAGAAAATACGGGATCTTCCAATATCCCTATTTGATTATT**
Sequence_403	**ATCTGAAAACGAAAGAAGTGCGCTGTAAGTATTACCAGCGCACTTCGGCAGCGGCAGCACCTCGGCAGCACCTCAGCAGCAACATGCCCAGCAAGAAGAATGGAAGAAGCGGACCACAACCTCATAAACGCTGGGTCTTCACGCTGAATAATCCTTCCGAAGACGAGCGCAAGAAAATACGGGATCTTCCAATATCCCTATTTGATTATT**
Sequence_404	**ATCTGAAAACGAAAGAAGTGCGCTGTAAGTATTACCAGCGCACTTCGGCAGCGGCAGCACCTCGGCAGCACCTCAGCAGCAACATGCCCAGCAAGAAGAATGGAAGAAGCGGACCGCAACCGCACAAAAGATGGGTCTTCACTCTGAATAATCCTTCCGAAGACGAGCGCAAGAAAATACGGGATCTTCCAATATCCCTATTTGATTATT**
Sequence_405	**ATCTGAAAACGAAAGAAGTGCGCTGTAAGTATTACCAGCGCACTTCGGCAGCGGCAGCACCTCGGCAGCACCTCAGCAGCAACATGCCCAGCAAGAAGAATGGAAGAAGCGGACCTCAACCATTAAAACGCTGGGTATTCACTCTGAATAATCCTTCCGAAGACGAGCGCAAGAAAATACGGGATCTTCCAATATCCCTATTTGATTATT**
Sequence_406	**ATCTGAAAACGAAAGAAGTGCGCTGTAAGTATTACCAGCGCACTTCGGCAGCGGCAGCACCTCGGCAGCACCTCAGCAGCAACATGCCCAGCAAGAAGAATGGAAGAAGCGGACCCCAACCCCATAAAAGGTGGGTGTTCACTCTAAACAACCCCTCGGAGGACGAGCGTAAGAAAATACGGGATCTTCCAATATCCCTATTTGATTATT**
Sequence_407	**ATCTGAAAACGAAAGAAGTGCGCTGTAAGTATTACCAGCGCACTTCGGCAGCGGCAGCACCTCGGCAGCACCTCAGCAGCAACATGCCCAGCAAGAAGAATGGAAGAAGCGGACCCCAACCCCATAAAAGGTGGGTGTTCAACCCTTCGGAAGAAGAAAAGAATAAGATTAAGAAAATACGGGATCTTCCAATATCCCTATTTGATTATT**
Sequence_408	**ATCTGAAAACGAAAGAAGTGCGCTGTAAGTATTACCAGCGCACTTCGGCAGCGGCAGCACCTCGGCAGCACCTCAGCAGCAACATGCCCAGCAAGAAGAATGGAAGAAGCGGACCCCAACCCCATAAAAGGTGGGTGTTCACCCTAGACAACCCCTCCGAAGATGAGCGCAAGAAAATACGGGATCTTCCAATATCCCTATTTGATTATT**
Sequence_409	**ATCTGAAAACGAAAGAAGTGCGCTGTAAGTATTACCAGCGCACTTCGGCAGCGGCAGCACCTCGGCAGCACCTCAGCAGCAACATGCCCAGCAAGAAGAATGGAAGAAGCGGACCCCAACCCCATAAAAGGTGGGTGTTCACGCTAAATAATCCATCAGAGGATGAACGCAAGAAAATACGGGATCTTCCAATATCCCTATTTGATTATT**
Sequence_410	**ATCTGAAAACGAAAGAAGTGCGCTGTAAGTATTACCAGCGCACTTCGGCAGCGGCAGCACCTCGGCAGCACCTCAGCAGCAACATGCCCAGCAAGAAGAATGGAAGAAGCGGACCCCAACCCCATAAAAGGTGGGTGTTCACGTTGAATGATCCTTCGGAGGACGAACGCAAGAAAATACGGGATCTTCCAAT**

	**ATCCCTATTTGATTATT**
Sequence_411	**ATCTGAAAACGAAAGAAGTGCGCTGTAAGTATTACCAGCGCACTTCG GCAGCGGCAGCACCTCGGCAGCACCTCAGCAGCAACATGCCCAGCAAGA AGAATGGAAGAAGCGGACCCCAACCCCATAAAAGGTGGGTGTTCACCCT TAATAATCCGTCCGAGGACGAACGCAAGAAAATACGGGATCTTCCAATA TCCCTATTTGATTATT**
Sequence_412	**ATCTGAAAACGAAAGAAGTGCGCTGTAAGTATTACCAGCGCACTTCG GCAGCGGCAGCACCTCGGCAGCACCTCAGCAGCAACATGCCCAGCAAGA AGAATGGAAGAAGCGGACCCCAACCCCATAAAAGGTGGGTGTTCACCTT AAACAATCCATCAGAAGACGAAAGAAAGAAAATACGGGATCTTCCAAT ATCCCTATTTGATTATT**
Sequence_413	**ATCTGAAAACGAAAGAAGTGCGCTGTAAGTATTACCAGCGCACTTCG GCAGCGGCAGCACCTCGGCAGCACCTCAGCAGCAACATGCCCAGCAAGA AGAATGGAAGAAGCGGACCCCAACCCCATAAAAGGTGGGTGTTCACCCT GAATAACCCGAGTGAAGATGAGCGTAAGAAAATACGGGATCTTCCAAT ATCCCTATTTGATTATT**
Sequence_414	**ATCTGAAAACGAAAGAAGTGCGCTGTAAGTATTACCAGCGCACTTCG GCAGCGGCAGCACCTCGGCAGCACCTCAGCAGCAACATGCCCAGCAAGA AGAATGGAAGAAGCGGACCCCAACCCCATAAAAGGTGGGTGTTCACAC TTGATAATCCATCAGAAGACGAACGGAAGAAAATACGGGATCTTCCAAT ATCCCTATTTGATTATT**
Sequence_415	**ATCTGAAAACGAAAGAAGTGCGCTGTAAGTATTACCAGCGCACTTCG GCAGCGGCAGCACCTCGGCAGCACCTCAGCAGCAACATGCCCAGCAAGA AGAATGGAAGAAGCGGACCCCAACCCCATAAAAGGTGGGTGTTCACAC TAAACAACCCCAGTGAGGACGAACGTAAGAAAATACGGGATCTTCCAAT ATCCCTATTTGATTATT**
Sequence_416	**ATCTGAAAACGAAAGAAGTGCGCTGTAAGTATTACCAGCGCACTTCG GCAGCGGCAGCACCTCGGCAGCACCTCAGCAGCAACATGCCCAGCAAGA AGAATGGAAGAAGCGGACCCCAACCCCATAAAAGGTGGGTGTTCACGC TAAACAATCCAAGTGAAGATGAACGAAAGAAAATACGGGATCTTCCAAT ATCCCTATTTGATTATT**
Sequence_417	**ATCTGAAAACGAAAGAAGTGCGCTGTAAGTATTACCAGCGCACTTCG GCAGCGGCAGCACCTCGGCAGCACCTCAGCAGCAACATGCCCAGCAAGA AGAATGGAAGAAGCGGACCCCAACCCCATAAAAGGTGGGTGTTCACGC TGAACAACCCTAGCGAGGATGAACGGAAGAAAATACGGGATCTTCCAA TATCCCTATTTGATTATT**
Sequence_418	**ATCTGAAAACGAAAGAAGTGCGCTGTAAGTATTACCAGCGCACTTCG GCAGCGGCAGCACCTCGGCAGCACCTCAGCAGCAACATGCCCAGCAAGA AGAATGGAAGAAGCGGACCCCAACCCCATAAAAGGTGGGTGTTCACTCT CAATAATCCCTCAGAAGACGAAAGAAAGAAAATACGGGATCTTCCAATA TCCCTATTTGATTATT**
Sequence_419	**ATCTGAAAACGAAAGAAGTGCGCTGTAAGTATTACCAGCGCACTTCG**

	**GCAGCGGCAGCACCTCGGCAGCACCTCAGCAGCAACATGCCCAGCAAGAAGAATGGAAGAAGCGGACCCCAACCCCATAAAAGGTGGGTGTTCACGCTAAACAATCCTTCTGAAGATGAGAGAAAGAAAATACGGGATCTTCCAATATCCCTATTTGATTATT**
Sequence_420	**ATCTGAAAACGAAAGAAGTGCGCTGTAAGTATTACCAGCGCACTTCGGCAGCGGCAGCACCTCGGCAGCACCTCAGCAGCAACATGCCCAGCAAGAAGAATGGAAGAAGCGGACCCCAACCCCATAAAAGGTGGGTGTTCACGGTAAATAACCCCTCGGAAGATGAGCGCAAGAAAATACGGGATCTTCCAATATCCCTATTTGATTATT**
Sequence_421	**ATCTGAAAACGAAAGAAGTGCGCTGTAAGTATTACCAGCGCACTTCGGCAGCGGCAGCACCTCGGCAGCACCTCAGCAGCAACATGCCCAGCAAGAAGAATGGAAGAAGCGGACCCCAACCCCATAAAAGGTGGGTGTTCACACTTAATAATCCGTCGGAAGCAGAGCGGAAGAAAATACGGGATCTTCCAATATCCCTATTTGATTATT**
Sequence_422	**ATCTGAAAACGAAAGAAGTGCGCTGTAAGTATTACCAGCGCACTTCGGCAGCGGCAGCACCTCGGCAGCACCTCAGCAGCAACATGCCCAGCAAGAAGAATGGAAGAAGCGGACCCCAACCCCATAAAAGGTGGGTGTTCACCCTCAACAACCCGAGCGAAAACGAACGGAAGAAAATACGGGATCTTCCAATATCCCTATTTGATTATT**
Sequence_423	**ATCTGAAAACGAAAGAAGTGCGCTGTAAGTATTACCAGCGCACTTCGGCAGCGGCAGCACCTCGGCAGCACCTCAGCAGCAACATGCCCAGCAAGAAGAATGGAAGAAGCGGACCCCAACCCCATAAAAGGTGGGTGTTCACACTGAATAACCCATCTGAAGGCGAACGTAAGAAAATACGGGATCTTCCAATATCCCTATTTGATTATT**
Sequence_424	**ATCTGAAAACGAAAGAAGTGCGCTGTAAGTATTACCAGCGCACTTCGGCAGCGGCAGCACCTCGGCAGCACCTCAGCAGCAACATGCCCAGCAAGAAGAATGGAAGAAGCGGACCCCAACCCCATAAAAGGTGGGTGTTCACACTGAATACTCCTTCTGAAGACGAACGCAAGAAAATACGGGATCTTCCAATATCCCTATTTGATTATT**
Sequence_425	**ATCTGAAAACGAAAGAAGTGCGCTGTAAGTATTACCAGCGCACTTCGGCAGCGGCAGCACCTCGGCAGCACCTCAGCAGCAACATGCCCAGCAAGAAGAATGGAAGAAGCGGACCCCAACCCCATAAAAGGTGGGTGTTCACGTTGGACAATCCATCTGAAGACGAGCGTAAGAAAATACGGGATCTTCCAATATCCCTATTTGATTATT**
Sequence_426	**ATCTGAAAACGAAAGAAGTGCGCTGTAAGTATTACCAGCGCACTTCGGCAGCGGCAGCACCTCGGCAGCACCTCAGCAGCAACATGCCCAGCAAGAAGAATGGAAGAAGCGGACCCCAACCCCATAAAAGGTGGGTGTTCACACTGAACAATCCTTCAGAGGCCGAGAGGAAGAAAATACGGGATCTTCCAATATCCCTATTTGATTATT**
Sequence_427	**ATCTGAAAACGAAAGAAGTGCGCTGTAAGTATTACCAGCGCACTTCGGCAGCGGCAGCACCTCGGCAGCACCTCAGCAGCAACATGCCCAGCAAGAAGAATGGAAGAAGCGGACCCCAACCCCATAAAAGGTGGGTGTTCACCCTCAACAACCCCAGCGAAGGTGAGAGAAAGAAAATACGGGATCTTCCAAT**

	**ATCCCTATTTGATTATT**
Sequence_428	**ATCTGAAAACGAAAGAAGTGCGCTGTAAGTATTACCAGCGCACTTCG GCAGCGGCAGCACCTCGGCAGCACCTCAGCAGCAACATGCCCAGCAAGA AGAATGGAAGAAGCGGACCCCAACCCCATAAAAGGTGGGTGTTCACAC TTAATAACCCATGTGAGGACGAACGGAAGAAAATACGGGATCTTCCAAT ATCCCTATTTGATTATT**
Sequence_429	**ATCTGAAAACGAAAGAAGTGCGCTGTAAGTATTACCAGCGCACTTCG GCAGCGGCAGCACCTCGGCAGCACCTCAGCAGCAACATGCCCAGCAAGA AGAATGGAAGAAGCGGACCCCAACCCCATAAAAGGTGGGTGTTCACGC TGAACACCCCAAGTGAAGACGAACGAAAGAAAATACGGGATCTTCCAA TATCCCTATTTGATTATT**
Sequence_430	**ATCTGAAAACGAAAGAAGTGCGCTGTAAGTATTACCAGCGCACTTCG GCAGCGGCAGCACCTCGGCAGCACCTCAGCAGCAACATGCCCAGCAAGA AGAATGGAAGAAGCGGACCCCAACCCCATAAAAGGTGGGTGTTCACAC TAAATAATCCAAGCGAAAATGAGCGCAAGAAAATACGGGATCTTCCAAT ATCCCTATTTGATTATT**
Sequence_431	**ATCTGAAAACGAAAGAAGTGCGCTGTAAGTATTACCAGCGCACTTCG GCAGCGGCAGCACCTCGGCAGCACCTCAGCAGCAACATGCCAAGCAAAA AAAACGGAAGAAGCGGACCCCAACCACACAAAAGATGGGTGTTCACAC TAAACAACCCCTCCGAAGACGAAAGAAAGAAAATACGGGATCTTCCAAT ATCCCTATTTGATTATT**
Sequence_432	**ATCTGAAAACGAAAGAAGTGCGCTGTAAGTATTACCAGCGCACTTCG GCAGCGGCAGCACCTCGGCAGCACCTCAGCAGCAACATGCCAAGCAAG AAAAATGGAAGAAGCGGACCCCAACCACACAAAAGATGGGTGTTCACA CTAAACAACCCTTCCGAAGACGAGAGAAAGAAAATACGGGATCTTCCAA TATCCCTATTTGATTATT**
Sequence_433	**ATCTGAAAACGAAAGAAGTGCGCTGTAAGTATTACCAGCGCACTTCG GCAGCGGCAGCACCTCGGCAGCACCTCAGCAGCAACATGCCCAGCAAGA AGAATGGAAGAAGCGGACCCCAACCACATAAAAGGTGGGTGTTCACAC TGAACAATCCCTCCGAAGACGAGAGAAAGAAAATACGGGATCTTCCAAT ATCCCTATTTGATTATT**
Sequence_434	**ATCTGAAAACGAAAGAAGTGCGCTGTAAGTATTACCAGCGCACTTCG GCAGCGGCAGCACCTCGGCAGCACCTCAGCAGCAACATGCCAAGCAAAA AGAACGGAAGAAGCGGACCCCAACCCCATAAAAGATGGGTGTTCACAC TAAACAACCCCTCCGAAGACGAACGCAAGAAAATACGGGATCTTCCAAT ATCCCTATTTGATTATT**
Sequence_435	**ATCTGAAAACGAAAGAAGTGCGCTGTAAGTATTACCAGCGCACTTCG GCAGCGGCAGCACCTCGGCAGCACCTCAGCAGCAACATGCCAAGCAAAA AAAATGGAAGAAGCGGACCCCAACCCCACAAAAGATGGGTGTTCACAC TAAACAACCCTTCCGAAGACGAGAGAAAGAAAATACGGGATCTTCCAAT ATCCCTATTTGATTATT**
Sequence_436	**ATCTGAAAACGAAAGAAGTGCGCTGTAAGTATTACCAGCGCACTTCG**

	**GCAGCGGCAGCACCTCGGCAGCACCTCAGCAGCAACATGCCCAGCAAAAAAAATGGAAGAAGCGGACCCCAACCCCACAAAAGGTGGGTGTTCACTCTAAACAATCCTTCCGAAGACGAGCGCAAGAAAATACGGGATCTTCCAATATCCCTATTTGATTATT**
Sequence_437	**ATCTGAAAACGAAAGAAGTGCGCTGTAAGTATTACCAGCGCACTTCGGCAGCGGCAGCACCTCGGCAGCACCTCAGCAGCAACATGCCCAGCAAAAAAAACGGAAGAAGCGGACCCCAACCACACAAAAGGTGGGTGTTCACACTAAATAACCCCTCCGAAGACGAACGCAAGAAAATACGGGATCTTCCAATATCCCTATTTGATTATT**
Sequence_438	**ATCTGAAAACGAAAGAAGTGCGCTGTAAGTATTACCAGCGCACTTCGGCAGCGGCAGCACCTCGGCAGCACCTCAGCAGCAACATGCCAAGCAAAAAAAATGGAAGAAGCGGACCCCAACCACACAAAAGATGGGTGTTCACTCTAAACAACCCTTCCGAAGACGAGAGAAAGAAAATACGGGATCTTCCAATATCCCTATTTGATTATT**
Sequence_439	**ATCTGAAAACGAAAGAAGTGCGCTGTAAGTATTACCAGCGCACTTCGGCAGCGGCAGCACCTCGGCAGCACCTCAGCAGCAACATGCCAAGCAAAAAAAATGGAAGAAGCGGACCCCAACCACACAAAAGATGGGTGTTCACTCTGAATAACCCCTCCGAAGACGAAAGAAAGAAAATACGGGATCTTCCAATATCCCTATTTGATTATT**
Sequence_440	**ATCTGAAAACGAAAGAAGTGCGCTGTAAGTATTACCAGCGCACTTCGGCAGCGGCAGCACCTCGGCAGCACCTCAGCAGCAACATGCCCAGCAAGAAAAACGGAAGAAGCGGACCCCAACCACATAAAAGGTGGGTGTTCACTCTAAATAATCCCTCCGAAGACGAGCGCAAGAAAATACGGGATCTTCCAATATCCCTATTTGATTATT**
Sequence_441	**ATCTGAAAACGAAAGAAGTGCGCTGTAAGTATTACCAGCGCACTTCGGCAGCGGCAGCACCTCGGCAGCACCTCAGCAGCAACATGCCCAGCAAGAAAAATGGAAGAAGCGGACCCCAACCCCACAAAAGATGGGTGTTCACACTGAATAATCCTTCCGAAGACGAACGCAAGAAAATACGGGATCTTCCAATATCCCTATTTGATTATT**
Sequence_442	**ATCTGAAAACGAAAGAAGTGCGCTGTAAGTATTACCAGCGCACTTCGGCAGCGGCAGCACCTCGGCAGCACCTCAGCAGCAACATGCCCAGCAAAAAGAACGGAAGAAGCGGACCCCAACCCCACAAAAGATGGGTGTTCACACTAAATAACCCCTCCGAAGACGAAAGAAAGAAAATACGGGATCTTCCAATATCCCTATTTGATTATT**
Sequence_443	**ATCTGAAAACGAAAGAAGTGCGCTGTAAGTATTACCAGCGCACTTCGGCAGCGGCAGCACCTCGGCAGCACCTCAGCAGCAACATGCCAAGCAAAAAGAACGGAAGAAGCGGACCCCAACCACATAAAAGATGGGTGTTCACACTAAATAACCCTTCCGAAGACGAAAGAAAGAAAATACGGGATCTTCCAATATCCCTATTTGATTATT**
Sequence_444	**ATCTGAAAACGAAAGAAGTGCGCTGTAAGTATTACCAGCGCACTTCGGCAGCGGCAGCACCTCGGCAGCACCTCAGCAGCAACATGCCCAGCAAAAAGAATGGAAGAAGCGGACCCCAACCCCACAAAAGGTGGGTGTTCACTCTAAATAATCCTTCCGAAGACGAAAGAAAGAAAATACGGGATCTTCCAATA**

	**TCCCTATTTGATTATT**
Sequence_445	**ATCTGAAAACGAAAGAAGTGCGCTGTAAGTATTACCAGCGCACTTCG GCAGCGGCAGCACCTCGGCAGCACCTCAGCAGCAACATGCCCAGCAAAA AAAACGGAAGAAGCGGACCCCAACCACACAAAAGATGGGTGTTCACTC TAAATAACCCCTCCGAAGACGAACGCAAGAAAATACGGGATCTTCCAAT ATCCCTATTTGATTATT**
Sequence_446	**ATCTGAAAACGAAAGAAGTGCGCTGTAAGTATTACCAGCGCACTTCG GCAGCGGCAGCACCTCGGCAGCACCTCAGCAGCAACATGCCAAGCAAAA AAAATGGAAGAAGCGGACCCCAACCACACAAAAGGTGGGTGTTCACAC TAAACAATCCTTCCGAAGACGAAAGAAAGAAAATACGGGATCTTCCAAT ATCCCTATTTGATTATT**
Sequence_447	**ATCTGAAAACGAAAGAAGTGCGCTGTAAGTATTACCAGCGCACTTCG GCAGCGGCAGCACCTCGGCAGCACCTCAGCAGCAACATGCCAAGCAAAA AAAACGGAAGAAGCGGACCCCAACCACATAAAAGATGGGTGTTCACAC TGAACAACCCTTCCGAAGACGAACGCAAGAAAATACGGGATCTTCCAAT ATCCCTATTTGATTATT**
Sequence_448	**ATCTGAAAACGAAAGAAGTGCGCTGTAAGTATTACCAGCGCACTTCG GCAGCGGCAGCACCTCGGCAGCACCTCAGCAGCAACATGCCCAGCAAGA AGAATGGAAGAAGCGGACCCCAACCCCACAAAAGATGGGTGTTCACAC TGAATAACCCTTCCGAAGACGAACGCAAGAAAATACGGGATCTTCCAAT ATCCCTATTTGATTATT**
Sequence_449	**ATCTGAAAACGAAAGAAGTGCGCTGTAAGTATTACCAGCGCACTTCG GCAGCGGCAGCACCTCGGCAGCACCTCAGCAGCAACATGCCCAGCAAGA AGAACGGAAGAAGCGGACCCCAACCACACAAAAGGTGGGTGTTCACAC TAAATAATCCTTCCGAAGACGAGCGCAAGAAAATACGGGATCTTCCAAT ATCCCTATTTGATTATT**
Sequence_450	**ATCTGAAAACGAAAGAAGTGCGCTGTAAGTATTACCAGCGCACTTCG GCAGCGGCAGCACCTCGGCAGCACCTCAGCAGCAACATGCCAAGCAAG AAGAATGGAAGAAGCGGACCCCAACCCCACAAAAGGTGGGTGTTCACA CTAAATAATCCTTCCGAAGACGAACGCAAGAAAATACGGGATCTTCCAA TATCCCTATTTGATTATT**
Sequence_451	**ATCTGAAAACGAAAGAAGTGCGCTGTAAGTATTACCAGCGCACTTCG GCAGCGGCAGCACCTCGGCAGCACCTCAGCAGCAACATGCCCAGCAAGA AGAATGGAAGAAGCGGACCCCAACCACATAAAAGGTGGGTGTTCACAC TAAACAACCCTTCCGAAGACGAGCGCAAGAAAATACGGGATCTTCCAAT ATCCCTATTTGATTATT**
Sequence_452	**ATCTGAAAACGAAAGAAGTGCGCTGTAAGTATTACCAGCGCACTTCG GCAGCGGCAGCACCTCGGCAGCACCTCAGCAGCAACATGCCCAGCAAGA AGAATGGAAGAAGCGGACCCCAACCACACAAAAGGTGGGTGTTCACAC TAAATAATCCTTCCGAAGACGAACGCAAGAAAATACGGGATCTTCCAAT ATCCCTATTTGATTATT**
Sequence_453	**ATCTGAAAACGAAAGAAGTGCGCTGTAAGTATTACCAGCGCACTTCG**

	**GCAGCGGCAGCACCTCGGCAGCACCTCAGCAGCAACATGCCAAGCAAAAAGAACGGAAGAAGCGGACCCCAACCCCACAAAAGATGGGTGTTCACACTGAATAACCCCTCCGAAGACGAAAGAAAGAAAATACGGGATCTTCCAATATCCCTATTTGATTATT**
Sequence_454	**ATCTGAAAACGAAAGAAGTGCGCTGTAAGTATTACCAGCGCACTTCGGCAGCGGCAGCACCTCGGCAGCACCTCAGCAGCAACATGCCCAGCAAAAAAAACGGAAGAAGCGGACCCCAACCCCACAAAAGATGGGTGTTCACACTGAACAACCCCTCCGAAGACGAACGCAAGAAAATACGGGATCTTCCAATATCCCTATTTGATTATT**
Sequence_455	**ATCTGAAAACGAAAGAAGTGCGCTGTAAGTATTACCAGCGCACTTCGGCAGCGGCAGCACCTCGGCAGCACCTCAGCAGCAACATGCCAAGCAAGAAAAATGGAAGAAGCGGACCCCAACCACACAAAAGATGGGTGTTCACACTGAACAATCCCTCCGAAGACGAAAGAAAGAAAATACGGGATCTTCCAATATCCCTATTTGATTATT**
Sequence_456	**ATCTGAAAACGAAAGAAGTGCGCTGTAAGTATTACCAGCGCACTTCGGCAGCGGCAGCACCTCGGCAGCACCTCAGCAGCAACATGCCAAGCAAGAAAAACGGAAGAAGCGGACCCCAACCACACAAAAGATGGGTGTTCACACTAAATAACCCCTCCGAAGACGAGCGCAAGAAAATACGGGATCTTCCAATATCCCTATTTGATTATT**
Sequence_457	**ATCTGAAAACGAAAGAAGTGCGCTGTAAGTATTACCAGCGCACTTCGGCAGCGGCAGCACCTCGGCAGCACCTCAGCAGCAACATGCCAAGCAAAAAAAATGGAAGAAGCGGACCCCAACCACACAAAAGGTGGGTGTTCACACTAAATAACCCTTCCGAAGACGAAAGAAAGAAAATACGGGATCTTCCAATATCCCTATTTGATTATT**
Sequence_458	**ATCTGAAAACGAAAGAAGTGCGCTGTAAGTATTACCAGCGCACTTCGGCAGCGGCAGCACCTCGGCAGCACCTCAGCAGCAACATGCCCAGCAAGAAGAATGGAAGAAGCGGACCCCAACCCCACAAAAGATGGGTGTTCACACTGAACAATCCTTCCGAAGACGAACGCAAGAAAATACGGGATCTTCCAATATCCCTATTTGATTATT**
Sequence_459	**ATCTGAAAACGAAAGAAGTGCGCTGTAAGTATTACCAGCGCACTTCGGCAGCGGCAGCACCTCGGCAGCACCTCAGCAGCAACATGCCAAGCAAAAAGAATGGAAGAAGCGGACCCCAACCACATAAAAGGTGGGTGTTCACACTGAATAATCCTTCCGAAGACGAGAGAAAGAAAATACGGGATCTTCCAATATCCCTATTTGATTATT**
Sequence_460	**ATCTGAAAACGAAAGAAGTGCGCTGTAAGTATTACCAGCGCACTTCGGCAGCGGCAGCACCTCGGCAGCACCTCAGCAGCAACATGCCAAGCAAAAAAAACGGAAGAAGCGGACCCCAACCACACAAAAGGTGGGTGTTCACTCTGAACAACCCTTCCGAAGACGAAAGAAAGAAAATACGGGATCTTCCAATATCCCTATTTGATTATT**
Sequence_461	**ATCTGAAAACGAAAGAAGTGCGCTGTAAGTATTACCAGCGCACTTCGGCAGCGGCAGCACCTCGGCAGCACCTCAGCAGCAACATGCCAAGCAAAAAAAATGGAAGAAGCGGACCCCAACCACACAAAAGATGGGTGTTCACACTAAATAATCCCTCCGAAGACGAACGCAAGAAAATACGGGATCTTCCAAT**

	**ATCCCTATTTGATTATT**
Sequence_462	**ATCTGAAAACGAAAGAAGTGCGCTGTAAGTATTACCAGCGCACTTCG GCAGCGGCAGCACCTCGGCAGCACCTCAGCAGCAACATGCCAAGCAAAA AAAACGGAAGAAGCGGACCCCAACCACATAAAAGATGGGTGTTCACTCT AAACAATCCCTCCGAAGACGAACGCAAGAAAATACGGGATCTTCCAATA TCCCTATTTGATTATT**
Sequence_463	**ATCTGAAAACGAAAGAAGTGCGCTGTAAGTATTACCAGCGCACTTCG GCAGCGGCAGCACCTCGGCAGCACCTCAGCAGCAACATGCCCAGCAAGA AGAACGGCCGGAGCGGCCCCCAGCCCCACAAGAGATGGGTGTTCACCCT GAACAACCCCAGCGAGGACGAGCGGAAGAAAATACGGGATCTTCCAAT ATCCCTATTTGATTATT**
Sequence_464	**ATCTGAAAACGAAAGAAGTGCGCTGTAAGTATTACCAGCGCACTTCG GCAGCGGCAGCACCTCGGCAGCACCTCAGCAGCAACATGCCCTCCAAGA AGAACGGCCGCTCCGGCCCCCAGCCCCACAAGAGATGGGTGTTCACCCT GAACAACCCCTCCGAGGACGAGCGCAAGAAAATACGGGATCTTCCAATA TCCCTATTTGATTATT**
Sequence_465	**ATCTGAAAACGAAAGAAGTGCGCTGTAAGTATTACCAGCGCACTTCG GCAGCGGCAGCACCTCGGCAGCACCTCAGCAGCAACATGCCCAGCAAGA AGAATGGAAGAAGCGGCCCCCAGCCCCACAAGAGATGGGTGTTCACCCT GAATAACCCCAGCGAAGACGAGCGGAAGAAAATACGGGATCTTCCAAT ATCCCTATTTGATTATT**
Sequence_466	**ATCTGAAAACGAAAGAAGTGCGCTGTAAGTATTACCAGCGCACTTCG GCAGCGGCAGCACCTCGGCAGCACCTCAGCAGCAACATGCCCAGCAAGA AGAACGGAAGAAGCGGACCCCAACCCCATAAGAGGTGGGTGTTCACCC TGAACAACCCTTCCGAAGACGAGCGCAAGAAAATACGGGATCTTCCAAT ATCCCTATTTGATTATT**
Sequence_467	**ATCTGAAAACGAAAGAAGTGCGCTGTAAGTATTACCAGCGCACTTCG GCAGCGGCAGCACCTCGGCAGCACCTCAGCAGCAACATGCCCAGCAAGA AGAACGGCCGGAGCGGACCCCAACCCCACAAGAGATGGGTGTTCACCCT GAACAACCCTTCCGAGGACGAGCGCAAGAAAATACGGGATCTTCCAATA TCCCTATTTGATTATT**
Sequence_468	**ATCTGAAAACGAAAGAAGTGCGCTGTAAGTATTACCAGCGCACTTCG GCAGCGGCAGCACCTCGGCAGCACCTCAGCAGCAACATGCCCAGCAAGA AGAATGGAAGAAGCGGACCCCAACCCCACAAAAGATGGGTGTTCACTCT GAACAATCCTAGCGAGGACGAGCGCAAGAAAATACGGGATCTTCCAAT ATCCCTATTTGATTATT**
Sequence_469	**ATCTGAAAACGAAAGAAGTGCGCTGTAAGTATTACCAGCGCACTTCG GCAGCGGCAGCACCTCGGCAGCACCTCAGCAGCAACATGCCCAGCAAGA AGAACGGACGGAGCGGACCCCAGCCCCATAAAAGATGGGTGTTCACTCT GAATAATCCCTCCGAAGACGAGCGCAAGAAAATACGGGATCTTCCAATA TCCCTATTTGATTATT**
Sequence_470	**ATCTGAAAACGAAAGAAGTGCGCTGTAAGTATTACCAGCGCACTTCG**

	**GCAGCGGCAGCACCTCGGCAGCACCTCAGCAGCAACATGCCCAGCAAGAAGAACGGAAGAAGCGGACCCCAGCCCCATAAAAGGTGGGTGTTCACCCTGAACAACCCTTCCGAAGACGAGCGCAAGAAAATACGGATCTTCCAATATCCCTATTTGATTATT**
Sequence_471	**ATCTGAAAACGAAAGAAGTGCGCTGTAAGTATTACCAGCGCACTTCGGCAGCGGCAGCACCTCGGCAGCACCTCAGCAGCAACATGCCCAGCAAGAAGAATGGACGGAGCGGACCCCAACCCCACAAAAGATGGGTGTTCACTCTGAACAACCCTTCCGAAGACGAGCGCAAGAAAATACGGATCTTCCAATATCCCTATTTGATTATT**
Sequence_472	**ATCTGAAAACGAAAGAAGTGCGCTGTAAGTATTACCAGCGCACTTCGGCAGCGGCAGCACCTCGGCAGCACCTCAGCAGCAACATGCCCAGCAAGAAGAATGGACGGAGCGGCCCCCAACCCCACAAGAGATGGGTGTTCACCCTGAACAACCCCTCCGAAGACGAGCGGAAGAAAATACGGATCTTCCAATATCCCTATTTGATTATT**
Sequence_473	**ATCTGAAAACGAAAGAAGTGCGCTGTAAGTATTACCAGCGCACTTCGGCAGCGGCAGCACCTCGGCAGCACCTCAGCAGCAACATGCCCAGCAAGAAGAACGGCAGAAGCGGACCCCAGCCCCATAAGAGATGGGTGTTCACCCTGAACAACCCCTCCGAGGACGAGCGCAAGAAAATACGGATCTTCCAATATCCCTATTTGATTATT**
Sequence_474	**ATCTGAAAACGAAAGAAGTGCGCTGTAAGTATTACCAGCGCACTTCGGCAGCGGCAGCACCTCGGCAGCACCTCAGCAGCAACATGCCCAGCAAGAAGAACGGCCGGAGCGGACCCCAGCCCCACAAGAGATGGGTGTTCACTCTGAATAACCCTTCCGAGGACGAGCGGAAGAAAATACGGATCTTCCAATATCCCTATTTGATTATT**
Sequence_475	**ATCTGAAAACGAAAGAAGTGCGCTGTAAGTATTACCAGCGCACTTCGGCAGCGGCAGCACCTCGGCAGCACCTCAGCAGCAACATGCCCAGCAAGAAGAATGGCAGAAGCGGCCCCCAACCCCACAAGAGATGGGTGTTCACCCTGAACAATCCCAGCGAGGACGAGCGCAAGAAAATACGGATCTTCCAATATCCCTATTTGATTATT**
Sequence_476	**ATCTGAAAACGAAAGAAGTGCGCTGTAAGTATTACCAGCGCACTTCGGCAGCGGCAGCACCTCGGCAGCACCTCAGCAGCAACATGCCCAGCAAGAAGAATGGCAGAAGCGGACCCCAGCCCCATAAAAGGTGGGTGTTCACTCTGAACAATCCCTCCGAAGACGAGCGGAAGAAAATACGGATCTTCCAATATCCCTATTTGATTATT**
Sequence_477	**ATCTGAAAACGAAAGAAGTGCGCTGTAAGTATTACCAGCGCACTTCGGCAGCGGCAGCACCTCGGCAGCACCTCAGCAGCAACATGCCCAGCAAGAAGAATGGAAGAAGCGGCCCCCAACCCCATAAAAGGTGGGTGTTCACTCTGAATAATCCCAGCGAGGACGAGCGGAAGAAAATACGGATCTTCCAATATCCCTATTTGATTATT**
Sequence_478	**ATCTGAAAACGAAAGAAGTGCGCTGTAAGTATTACCAGCGCACTTCGGCAGCGGCAGCACCTCGGCAGCACCTCAGCAGCAACATGCCCAGCAAGAAGAATGGAAGAAGCGGCCCCCAACCCCATAAAAGGTGGGTGTTCACCCTGAACAATCCCTCCGAAGACGAGCGGAAGAAAATACGGATCTTCCAATA**

	**TCCCTATTTGATTATT**
Sequence_479	**ATCTGAAAACGAAAGAAGTGCGCTGTAAGTATTACCAGCGCACTTCGGCAGCGGCAGCACCTCGGCAGCACCTCAGCAGCAACATGCCCAGCAAGAAGAATGGAAGAAGCGGCCCCCAGCCCCACAAGAGGTGGGTGTTCACCCTGAACAACCCCAGCGAAGACGAGCGGAAGAAAATACGGGATCTTCCAATATCCCTATTTGATTATT**
Sequence_480	**ATCTGAAAACGAAAGAAGTGCGCTGTAAGTATTACCAGCGCACTTCGGCAGCGGCAGCACCTCGGCAGCACCTCAGCAGCAACATGCCCAGCAAGAAGAATGGAAGAAGCGGACCCCAGCCCCACAAAAGGTGGGTGTTCACTCTGAATAACCCTAGCGAGGACGAGCGCAAGAAAATACGGGATCTTCCAATATCCCTATTTGATTATT**
Sequence_481	**ATCTGAAAACGAAAGAAGTGCGCTGTAAGTATTACCAGCGCACTTCGGCAGCGGCAGCACCTCGGCAGCACCTCAGCAGCAACATGCCCAGCAAGAAGAATGGCCGGAGCGGACCCCAACCCCACAAAAGGTGGGTGTTCACCCTGAATAATCCTTCCGAGGACGAGCGCAAGAAAATACGGGATCTTCCAATATCCCTATTTGATTATT**
Sequence_482	**ATCTGAAAACGAAAGAAGTGCGCTGTAAGTATTACCAGCGCACTTCGGCAGCGGCAGCACCTCGGCAGCACCTCAGCAGCAACATGCCCAGCAAGAAGAATGGAAGAAGCGGCCCCCAGCCCCATAAAAGATGGGTGTTCACTCTGAATAATCCCTCCGAAGACGAGCGGAAGAAAATACGGGATCTTCCAATATCCCTATTTGATTATT**
Sequence_483	**ATCTGAAAACGAAAGAAGTGCGCTGTAAGTATTACCAGCGCACTTCGGCAGCGGCAGCACCTCGGCAGCACCTCAGCAGCAACATGCCCTCCAAGAAGAACGGCAGAAGCGGCCCCCAACCCCACAAAAGATGGGTGTTCACCCTGAACAACCCTTCCGAGGACGAGCGCAAGAAAATACGGGATCTTCCAATATCCCTATTTGATTATT**
Sequence_484	**ATCTGAAAACGAAAGAAGTGCGCTGTAAGTATTACCAGCGCACTTCGGCAGCGGCAGCACCTCGGCAGCACCTCAGCAGCAACATGCCCTCCAAGAAGAATGGACGCTCCGGACCCCAACCCCATAAAAGATGGGTGTTCACTCTGAATAATCCTTCCGAGGACGAGCGCAAGAAAATACGGGATCTTCCAATATCCCTATTTGATTATT**
Sequence_485	**ATCTGAAAACGAAAGAAGTGCGCTGTAAGTATTACCAGCGCACTTCGGCAGCGGCAGCACCTCGGCAGCACCTCAGCAGCAACATGCCCTCCAAGAAGAATGGAAGATCCGGACCCCAACCCCATAAAAGGTGGGTGTTCACTCTGAACAACCCCTCCGAAGACGAGCGCAAGAAAATACGGGATCTTCCAATATCCCTATTTGATTATT**
Sequence_486	**ATCTGAAAACGAAAGAAGTGCGCTGTAAGTATTACCAGCGCACTTCGGCAGCGGCAGCACCTCGGCAGCACCTCAGCAGCAACATGCCCAGCAAGAAGAACGGAAGATCCGGACCCCAACCCCATAAAAGGTGGGTGTTCACCCTGAATAACCCTTCCGAGGACGAGCGCAAGAAAATACGGGATCTTCCAATATCCCTATTTGATTATT**
Sequence_487	**ATCTGAAAACGAAAGAAGTGCGCTGTAAGTATTACCAGCGCACTTCG**

	GCAGCGGCAGCACCTCGGCAGCACCTCAGCAGCAACATGCCCAGCAAGA AGAACGGAAGAAGCGGACCCCAACCCCACAAAAGGTGGGTGTTCACCC TGAACAACCCTTCCGAAGACGAGCGCAAGAAAATACGGGATCTTCCAAT ATCCCTATTTGATTATT
Sequence_488	ATCTGAAAACGAAAGAAGTGCGCTGTAAGTATTACCAGCGCACTTCG GCAGCGGCAGCACCTCGGCAGCACCTCAGCAGCAACATGCCCAGCAAGA AGAACGGAAGAAGCGGACCCCAGCCCCATAAAAGGTGGGTGTTCACTCT GAACAATCCCTCCGAGGACGAGCGCAAGAAAATACGGGATCTTCCAATA TCCCTATTTGATTATT
Sequence_489	ATCTGAAAACGAAAGAAGTGCGCTGTAAGTATTACCAGCGCACTTCG GCAGCGGCAGCACCTCGGCAGCACCTCAGCAGCAACATGCCCAGCAAGA AGAATGGAAGAAGCGGACCCCAACCCCACAAGAGGTGGGTGTTCACTCT GAATAACCCCTCCGAGGACGAGCGCAAGAAAATACGGGATCTTCCAATA TCCCTATTTGATTATT
Sequence_490	ATCTGAAAACGAAAGAAGTGCGCTGTAAGTATTACCAGCGCACTTCG GCAGCGGCAGCACCTCGGCAGCACCTCAGCAGCAACATGCCCAGCAAGA AGAATGGCAGATCCGGCCCCCAGCCCCACAAAAGATGGGTGTTCACCCT GAACAATCCCTCCGAGGACGAGCGCAAGAAAATACGGGATCTTCCAATA TCCCTATTTGATTATT
Sequence_491	ATCTGAAAACGAAAGAAGTGCGCTGTAAGTATTACCAGCGCACTTCG GCAGCGGCAGCACCTCGGCAGCACCTCAGCAGCAACATGCCCAGCAAGA AGAACGGAAGAAGCGGACCCCAACCCCATAAAAGGTGGGTGTTCACCCT GAACAATCCCTCCGAGGACGAGCGCAAGAAAATACGGGATCTTCCAATA TCCCTATTTGATTATT
Sequence_492	ATCTGAAAACGAAAGAAGTGCGCTGTAAGTATTACCAGCGCACTTCG GCAGCGGCAGCACCTCGGCAGCACCTCAGCAGCAACATGCCCAGCAAGA AGAACGGCCGCAGCGGCCCCCAGCCCCACAAAAGATGGGTGTTCACCCT GAATAATCCCTCCGAGGACGAGCGCAAGAAAATACGGGATCTTCCAATA TCCCTATTTGATTATT
Sequence_493	ATCTGAAAACGAAAGAAGTGCGCTGTAAGTATTACCAGCGCACTTCG GCAGCGGCAGCACCTCGGCAGCACCTCAGCAGCAACATGCCCAGCAAGA AGAACGGCAGATCCGGCCCCCAGCCCCACAAAAGGTGGGTGTTCACCCT GAACAACCCTTCCGAGGACGAGCGCAAGAAAATACGGGATCTTCCAATA TCCCTATTTGATTATT
Sequence_494	ATCTGAAAACGAAAGAAGTGCGCTGTAAGTATTACCAGCGCACTTCG GCAGCGGCAGCACCTCGGCAGCACCTCAGCAGCAACATGCCCTCCAAGA AGAACGGACGCAGCGGACCCCAACCCCATAAAAGGTGGGTGTTCACTCT GAATAACCCTTCCGAGGACGAGCGCAAGAAAATACGGGATCTTCCAATA TCCCTATTTGATTATT
Sequence_495	ATCTGAAAACGAAAGAAGTGCGCTGTAAGTATTACCAGCGCACTTCG GCAGCGGCAGCACCTCGGCAGCACCTCAGCAGCAACATGCCCTCCAAGA AGAACGGCCGCTCCGGCCCCCAGCCCCACAAAAGGTGGGTGTTCACTCT GAATAATCCCTCCGAGGACGAGCGCAAGAAAATACGGGATCTTCCAATA

	**TCCCTATTTGATTATT**
Sequence_496	**ATCTGAAAACGAAAGAAGTGCGCTGTAAGTATTACCAGCGCACTTCG GCAGCGGCAGCACCTCGGCAGCACCTCAGCAGCAACATGCCCAGCAAGA AGAATGGAAGAAGCGGCCCCCAGCCCCACAAAAGGTGGGTGTTCACTCT GAATAACCCTTCCGAGGACGAGCGCAAGAAAATACGGGATCTTCCAATA TCCCTATTTGATTATT**
Sequence_497	**ATCTGAAAACGAAAGAAGTGCGCTGTAAGTATTACCAGCGCACTTCG GCAGCGGCAGCACCTCGGCAGCACCTCAGCAGCAACATGCCCTCCAAGA AGAATGGCCGCAGCGGCCCCCAGCCCCATAAGAGGTGGGTGTTCACCCT GAACAACCCCTCCGAAGACGAGCGCAAGAAAATACGGGATCTTCCAATA TCCCTATTTGATTATT**
Sequence_498	**ATCTGAAAACGAAAGAAGTGCGCTGTAAGTATTACCAGCGCACTTCG GCAGCGGCAGCACCTCGGCAGCACCTCAGCAGCAACATGCCCAGCAAGA AGAATGGCAGATCCGGACCCCAGCCCCATAAAAGGTGGGTGTTCACCCT GAATAATCCCTCCGAAGACGAGCGCAAGAAAATACGGGATCTTCCAATA TCCCTATTTGATTATT**
Sequence_499	**ATCTGAAAACGAAAGAAGTGCGCTGTAAGTATTACCAGCGCACTTCG GCAGCGGCAGCACCTCGGCAGCACCTCAGCAGCAACATGCCCAGCAAGA AGAACGGCAGAAGCGGCCCCCAGCCCCACAAAAGATGGGTGTTCACCCT GAATAACCCCTCCGAGGACGAGCGCAAGAAAATACGGGATCTTCCAATA TCCCTATTTGATTATT**
Sequence_500	**ATCTGAAAACGAAAGAAGTGCGCTGTAAGTATTACCAGCGCACTTCG GCAGCGGCAGCACCTCGGCAGCACCTCAGCAGCAACATGCCCTCCAAGA AGAACGGCCGCTCCGGACCCCAGCCCCACAAAAGATGGGTGTTCACCCT GAACAATCCTTCCGAAGACGAGCGCAAGAAAATACGGGATCTTCCAATA TCCCTATTTGATTATT**

Table S3. Synthetic library sequences in the edited region (marked in bold), the full sequence of the wildtype sequence is at the 1st row (NCBI accesstion number KJ128273)

**NCBI accession number**	**Type**		**NCBI accession number**	**Type**
AF071879	PCV1		KC261600	PCV2
AY099501	PCV1		KC261601	PCV2
AY184287	PCV1		KC336417	PCV2
AY219836	PCV1		KC336418	PCV2
AY660574	PCV1		KC447454	PCV2
AY699796	PCV1		KC473165	PCV2
AY754012	PCV1		KC473166	PCV2
AY754013	PCV1		KC473167	PCV2
AY754014	PCV1		KC473168	PCV2
AY754015	PCV1		KC514965	PCV2
DQ358813	PCV1		KC514966	PCV2
DQ472012	PCV1		KC514967	PCV2

DQ472013	PCV1		KC514968	PCV2
DQ472014	PCV1		KC514969	PCV2
DQ472015	PCV1		KC514970	PCV2
DQ472016	PCV1		KC514971	PCV2
DQ494787	PCV1		KC514972	PCV2
DQ494788	PCV1		KC514973	PCV2
DQ648032	PCV1		KC514974	PCV2
DQ650650	PCV1		KC514975	PCV2
DQ659153	PCV1		KC514976	PCV2
DQ659154	PCV1		KC514977	PCV2
EF493843	PCV1		KC514978	PCV2
EF533941	PCV1		KC514979	PCV2
FJ159689	PCV1		KC514980	PCV2
FJ159690	PCV1		KC514981	PCV2
FJ159691	PCV1		KC514982	PCV2
FJ159692	PCV1		KC514983	PCV2
FJ159693	PCV1		KC514984	PCV2
FJ475129	PCV1		KC514985	PCV2
FJ655418	PCV1		KC514986	PCV2
FJ655419	PCV1		KC514987	PCV2
FJ790425	PCV1		KC514988	PCV2
GU371908	PCV1		KC514989	PCV2
GU799575	PCV1		KC514990	PCV2
HM143844	PCV1		KC514991	PCV2
JN133302	PCV1		KC514992	PCV2
JN133303	PCV1		KC514993	PCV2
JN398656	PCV1		KC514994	PCV2
JX566507	PCV1		KC514995	PCV2
KC447455	PCV1		KC514996	PCV2
KC733436	PCV1		KC514997	PCV2
KC878437	PCV1		KC514998	PCV2
KC894933	PCV1		KC514999	PCV2
KC924758	PCV1		KC515000	PCV2
KF732857	PCV1		KC515001	PCV2
KJ408798	PCV1		KC515002	PCV2
KJ408799	PCV1		KC515003	PCV2
KJ746929	PCV1		KC515004	PCV2
KJ746930	PCV1		KC515005	PCV2
KJ808815	PCV1		KC515006	PCV2
KX816645	PCV1		KC515007	PCV2
KX827778	PCV1		KC515008	PCV2
KX827779	PCV1		KC515009	PCV2
KX827780	PCV1		KC515010	PCV2
KX827781	PCV1		KC515011	PCV2
KX827782	PCV1		KC515012	PCV2

KX827783	PCV1		KC515013	PCV2
KX827784	PCV1		KC515014	PCV2
KX827785	PCV1		KC515015	PCV2
KX827786	PCV1		KC515016	PCV2
KX827787	PCV1		KC515017	PCV2
KX827788	PCV1		KC515018	PCV2
KX827789	PCV1		KC515019	PCV2
KX827790	PCV1		KC515020	PCV2
KY437725	PCV1		KC515021	PCV2
NC_001792	PCV1		KC515022	PCV2
NC_013774	PCV1		KC515023	PCV2
AB072301	PCV2		KC515024	PCV2
AB072302	PCV2		KC515025	PCV2
AB072303	PCV2		KC515026	PCV2
AB426905	PCV2		KC515027	PCV2
AF027217	PCV2		KC515028	PCV2
AF055391	PCV2		KC515029	PCV2
AF055392	PCV2		KC527542	PCV2
AF055393	PCV2		KC533811	PCV2
AF055394	PCV2		KC533812	PCV2
AF085695	PCV2		KC618389	PCV2
AF086834	PCV2		KC684978	PCV2
AF086835	PCV2		KC688418	PCV2
AF086836	PCV2		KC688419	PCV2
AF109399	PCV2		KC688420	PCV2
AF147751	PCV2		KC751546	PCV2
AF201305	PCV2		KC753768	PCV2
AF201306	PCV2		KC753769	PCV2
AF201307	PCV2		KC753770	PCV2
AF201308	PCV2		KC753771	PCV2
AF201309	PCV2		KC753772	PCV2
AF201310	PCV2		KC788504	PCV2
AF201311	PCV2		KC800634	PCV2
AF201897	PCV2		KC800635	PCV2
AF264038	PCV2		KC800636	PCV2
AF264039	PCV2		KC800637	PCV2
AF264040	PCV2		KC800638	PCV2
AF264041	PCV2		KC800639	PCV2
AF264042	PCV2		KC800640	PCV2
AF264043	PCV2		KC800641	PCV2
AF381175	PCV2		KC800642	PCV2
AF381176	PCV2		KC800643	PCV2
AF381177	PCV2		KC800644	PCV2
AF408635	PCV2		KC800645	PCV2
AF454546	PCV2		KC800646	PCV2

AF465211	PCV2		KC821781	PCV2
AF520783	PCV2		KC821782	PCV2
AF538325	PCV2		KC821783	PCV2
AF544024	PCV2		KC821784	PCV2
AM086384	PCV2		KC821785	PCV2
AY035820	PCV2		KC823053	PCV2
AY094619	PCV2		KC823054	PCV2
AY099495	PCV2		KC823055	PCV2
AY099496	PCV2		KC823056	PCV2
AY099497	PCV2		KC835190	PCV2
AY099498	PCV2		KC835191	PCV2
AY099499	PCV2		KC835192	PCV2
AY099500	PCV2		KC835193	PCV2
AY122275	PCV2		KC835194	PCV2
AY146991	PCV2		KC859451	PCV2
AY146992	PCV2		KC860786	PCV2
AY146993	PCV2		KC907703	PCV2
AY177626	PCV2		KC924956	PCV2
AY180396	PCV2		KF027491	PCV2
AY180397	PCV2		KF027492	PCV2
AY181945	PCV2		KF027493	PCV2
AY181946	PCV2		KF027494	PCV2
AY181947	PCV2		KF027495	PCV2
AY181948	PCV2		KF027496	PCV2
AY188355	PCV2		KF027497	PCV2
AY217743	PCV2		KF374705	PCV2
AY256455	PCV2		KF530836	PCV2
AY256456	PCV2		KF530837	PCV2
AY256457	PCV2		KF695388	PCV2
AY256458	PCV2		KF732649	PCV2
AY256459	PCV2		KF742540	PCV2
AY256460	PCV2		KF742541	PCV2
AY288133	PCV2		KF742542	PCV2
AY288134	PCV2		KF742543	PCV2
AY288135	PCV2		KF742544	PCV2
AY291316	PCV2		KF742545	PCV2
AY291317	PCV2		KF742546	PCV2
AY291318	PCV2		KF742547	PCV2
AY294310	PCV2		KF742548	PCV2
AY321982	PCV2		KF742549	PCV2
AY321983	PCV2		KF742550	PCV2
AY321984	PCV2		KF742551	PCV2
AY321985	PCV2		KF742552	PCV2
AY321986	PCV2		KF742553	PCV2
AY321987	PCV2		KF850458	PCV2

AY321988	PCV2		KF850459	PCV2
AY321989	PCV2		KF850460	PCV2
AY321990	PCV2		KF850461	PCV2
AY321991	PCV2		KF850462	PCV2
AY321992	PCV2		KF850463	PCV2
AY321993	PCV2		KF850464	PCV2
AY321994	PCV2		KF850465	PCV2
AY321995	PCV2		KF850466	PCV2
AY321996	PCV2		KF850467	PCV2
AY321997	PCV2		KF850468	PCV2
AY321998	PCV2		KF850469	PCV2
AY321999	PCV2		KF871067	PCV2
AY322000	PCV2		KF871068	PCV2
AY322001	PCV2		KF926650	PCV2
AY322002	PCV2		KJ094599	PCV2
AY322003	PCV2		KJ094600	PCV2
AY322004	PCV2		KJ094601	PCV2
AY325495	PCV2		KJ094602	PCV2
AY391729	PCV2		KJ094603	PCV2
AY424401	PCV2		KJ094604	PCV2
AY424402	PCV2		KJ094605	PCV2
AY424403	PCV2		KJ094606	PCV2
AY424404	PCV2		KJ128269	PCV2
AY424405	PCV2		KJ128270	PCV2
AY484407	PCV2		KJ128271	PCV2
AY484408	PCV2		KJ128272	PCV2
AY484409	PCV2		KJ128273	PCV2
AY484410	PCV2		KJ128274	PCV2
AY484411	PCV2		KJ133547	PCV2
AY484412	PCV2		KJ139962	PCV2
AY484413	PCV2		KJ187306	PCV2
AY484414	PCV2		KJ437192	PCV2
AY484415	PCV2		KJ437506	PCV2
AY484416	PCV2		KJ511870	PCV2
AY510375	PCV2		KJ511871	PCV2
AY536755	PCV2		KJ511872	PCV2
AY536756	PCV2		KJ511873	PCV2
AY556473	PCV2		KJ511874	PCV2
AY556474	PCV2		KJ511875	PCV2
AY556475	PCV2		KJ511876	PCV2
AY556476	PCV2		KJ511877	PCV2
AY556477	PCV2		KJ596438	PCV2
AY578327	PCV2		KJ599673	PCV2
AY579893	PCV2		KJ679445	PCV2
AY596823	PCV2		KJ679446	PCV2

AY604430	PCV2		KJ680340	PCV2
AY613854	PCV2		KJ680341	PCV2
AY641542	PCV2		KJ680342	PCV2
AY651850	PCV2		KJ680343	PCV2
AY678532	PCV2		KJ680344	PCV2
AY682990	PCV2		KJ680345	PCV2
AY682991	PCV2		KJ680346	PCV2
AY682992	PCV2		KJ680347	PCV2
AY682993	PCV2		KJ680348	PCV2
AY682994	PCV2		KJ680349	PCV2
AY682995	PCV2		KJ680350	PCV2
AY682996	PCV2		KJ680351	PCV2
AY682997	PCV2		KJ680352	PCV2
AY686762	PCV2		KJ680353	PCV2
AY686763	PCV2		KJ680354	PCV2
AY686764	PCV2		KJ680355	PCV2
AY686765	PCV2		KJ680356	PCV2
AY691169	PCV2		KJ680357	PCV2
AY691679	PCV2		KJ680358	PCV2
AY699793	PCV2		KJ680359	PCV2
AY713470	PCV2		KJ680360	PCV2
AY732494	PCV2		KJ680361	PCV2
AY754016	PCV2		KJ680362	PCV2
AY754017	PCV2		KJ680363	PCV2
AY754018	PCV2		KJ680364	PCV2
AY754019	PCV2		KJ680365	PCV2
AY754020	PCV2		KJ680366	PCV2
AY754021	PCV2		KJ680367	PCV2
AY754022	PCV2		KJ680368	PCV2
AY849938	PCV2		KJ680369	PCV2
AY874163	PCV2		KJ680370	PCV2
AY874164	PCV2		KJ729072	PCV2
AY874165	PCV2		KJ729073	PCV2
AY874166	PCV2		KJ729074	PCV2
AY874167	PCV2		KJ729075	PCV2
AY874168	PCV2		KJ778679	PCV2
AY874169	PCV2		KJ778680	PCV2
AY916791	PCV2		KJ867553	PCV2
AY943819	PCV2		KJ867554	PCV2
AY969004	PCV2		KJ867555	PCV2
DQ017036	PCV2		KJ867556	PCV2
DQ104419	PCV2		KJ920205	PCV2
DQ104420	PCV2		KJ946351	PCV2
DQ104421	PCV2		KJ956689	PCV2
DQ104422	PCV2		KJ956690	PCV2

DQ104423	PCV2		KJ956691	PCV2
DQ141322	PCV2		KJ956692	PCV2
DQ180392	PCV2		KM035760	PCV2
DQ180393	PCV2		KM035761	PCV2
DQ195679	PCV2		KM035762	PCV2
DQ201641	PCV2		KM042394	PCV2
DQ201642	PCV2		KM042395	PCV2
DQ206444	PCV2		KM042396	PCV2
DQ218419	PCV2		KM042397	PCV2
DQ218420	PCV2		KM042398	PCV2
DQ218421	PCV2		KM042399	PCV2
DQ220727	PCV2		KM042400	PCV2
DQ220728	PCV2		KM042401	PCV2
DQ220729	PCV2		KM042402	PCV2
DQ220730	PCV2		KM042403	PCV2
DQ220731	PCV2		KM042404	PCV2
DQ220732	PCV2		KM042405	PCV2
DQ220733	PCV2		KM042406	PCV2
DQ220734	PCV2		KM042407	PCV2
DQ220735	PCV2		KM067384	PCV2
DQ220736	PCV2		KM067385	PCV2
DQ220737	PCV2		KM096530	PCV2
DQ220738	PCV2		KM116513	PCV2
DQ220739	PCV2		KM116514	PCV2
DQ233257	PCV2		KM116515	PCV2
DQ364650	PCV2		KM235959	PCV2
DQ397521	PCV2		KM235960	PCV2
DQ629113	PCV2		KM235961	PCV2
DQ629114	PCV2		KM245558	PCV2
DQ629115	PCV2		KM272211	PCV2
DQ629116	PCV2		KM272212	PCV2
DQ629117	PCV2		KM360049	PCV2
DQ629118	PCV2		KM360050	PCV2
DQ629119	PCV2		KM360051	PCV2
DQ648031	PCV2		KM360052	PCV2
DQ861895	PCV2		KM360053	PCV2
DQ861896	PCV2		KM360054	PCV2
DQ861897	PCV2		KM360055	PCV2
DQ861898	PCV2		KM360056	PCV2
DQ861899	PCV2		KM360057	PCV2
DQ861900	PCV2		KM434195	PCV2
DQ861901	PCV2		KM460823	PCV2
DQ861902	PCV2		KM460824	PCV2
DQ870484	PCV2		KM487708	PCV2
DQ910865	PCV2		KM487709	PCV2

DQ910866	PCV2		KM503044	PCV2
DQ915583	PCV2		KM604666	PCV2
DQ915584	PCV2		KM604667	PCV2
DQ915585	PCV2		KM624030	PCV2
DQ915586	PCV2		KM624031	PCV2
DQ915587	PCV2		KM624032	PCV2
DQ915588	PCV2		KM624033	PCV2
DQ923523	PCV2		KM624034	PCV2
DQ923524	PCV2		KM624035	PCV2
DQ997815	PCV2		KM624036	PCV2
DQ997816	PCV2		KM624037	PCV2
DQ997817	PCV2		KM624038	PCV2
EF210106	PCV2		KM624039	PCV2
EF394774	PCV2		KM880080	PCV2
EF394775	PCV2		KM880081	PCV2
EF394776	PCV2		KM880082	PCV2
EF394777	PCV2		KM880083	PCV2
EF394778	PCV2		KM880084	PCV2
EF394779	PCV2		KM880085	PCV2
EF421967	PCV2		KM880086	PCV2
EF421968	PCV2		KM880087	PCV2
EF421969	PCV2		KM924364	PCV2
EF421970	PCV2		KM924366	PCV2
EF421971	PCV2		KM924367	PCV2
EF421972	PCV2		KM924369	PCV2
EF421973	PCV2		KM974911	PCV2
EF452350	PCV2		KM975682	PCV2
EF452351	PCV2		KM975683	PCV2
EF452352	PCV2		KP081538	PCV2
EF452353	PCV2		KP081539	PCV2
EF458306	PCV2		KP081540	PCV2
EF493837	PCV2		KP081541	PCV2
EF493838	PCV2		KP081542	PCV2
EF493839	PCV2		KP081543	PCV2
EF493840	PCV2		KP081544	PCV2
EF493841	PCV2		KP081545	PCV2
EF493842	PCV2		KP081546	PCV2
EF515839	PCV2		KP081547	PCV2
EF524515	PCV2		KP081548	PCV2
EF524516	PCV2		KP081549	PCV2
EF524517	PCV2		KP081550	PCV2
EF524518	PCV2		KP081551	PCV2
EF524519	PCV2		KP081552	PCV2
EF524520	PCV2		KP081553	PCV2
EF524521	PCV2		KP081554	PCV2

EF524522	PCV2		KP081555	PCV2
EF524524	PCV2		KP081556	PCV2
EF524525	PCV2		KP112484	PCV2
EF524527	PCV2		KP112485	PCV2
EF524528	PCV2		KP112486	PCV2
EF524529	PCV2		KP179231	PCV2
EF524530	PCV2		KP231099	PCV2
EF524533	PCV2		KP231100	PCV2
EF524534	PCV2		KP231101	PCV2
EF524535	PCV2		KP231102	PCV2
EF524538	PCV2		KP231103	PCV2
EF524539	PCV2		KP231104	PCV2
EF524540	PCV2		KP231105	PCV2
EF524542	PCV2		KP231106	PCV2
EF565342	PCV2		KP231107	PCV2
EF565343	PCV2		KP231108	PCV2
EF565344	PCV2		KP231109	PCV2
EF565345	PCV2		KP231110	PCV2
EF565346	PCV2		KP231111	PCV2
EF565347	PCV2		KP231112	PCV2
EF565348	PCV2		KP231113	PCV2
EF565349	PCV2		KP231114	PCV2
EF565350	PCV2		KP231115	PCV2
EF565351	PCV2		KP231116	PCV2
EF565352	PCV2		KP231117	PCV2
EF565353	PCV2		KP231118	PCV2
EF565354	PCV2		KP231119	PCV2
EF565355	PCV2		KP231120	PCV2
EF565356	PCV2		KP231121	PCV2
EF565357	PCV2		KP231122	PCV2
EF565358	PCV2		KP231123	PCV2
EF565359	PCV2		KP231124	PCV2
EF565360	PCV2		KP231125	PCV2
EF565361	PCV2		KP231126	PCV2
EF565362	PCV2		KP231127	PCV2
EF565363	PCV2		KP231128	PCV2
EF565364	PCV2		KP231129	PCV2
EF565365	PCV2		KP231130	PCV2
EF565366	PCV2		KP231131	PCV2
EF565367	PCV2		KP231132	PCV2
EF565368	PCV2		KP231133	PCV2
EF592575	PCV2		KP231134	PCV2
EF592576	PCV2		KP231135	PCV2
EF619037	PCV2		KP231136	PCV2
EF675229	PCV2		KP231137	PCV2

EF675230	PCV2		KP231138	PCV2
EF675231	PCV2		KP231139	PCV2
EF675232	PCV2		KP231140	PCV2
EF675233	PCV2		KP231141	PCV2
EF675234	PCV2		KP231142	PCV2
EF675235	PCV2		KP231143	PCV2
EF675236	PCV2		KP231144	PCV2
EF675237	PCV2		KP231145	PCV2
EF675239	PCV2		KP231146	PCV2
EF675240	PCV2		KP231147	PCV2
EF675241	PCV2		KP231148	PCV2
EF675242	PCV2		KP231149	PCV2
EF675244	PCV2		KP231150	PCV2
EF989713	PCV2		KP231151	PCV2
EU057184	PCV2		KP231152	PCV2
EU057185	PCV2		KP231153	PCV2
EU057186	PCV2		KP231154	PCV2
EU057187	PCV2		KP231155	PCV2
EU057188	PCV2		KP231156	PCV2
EU057189	PCV2		KP231157	PCV2
EU095020	PCV2		KP231158	PCV2
EU126887	PCV2		KP231159	PCV2
EU136711	PCV2		KP231160	PCV2
EU136712	PCV2		KP231161	PCV2
EU136713	PCV2		KP231162	PCV2
EU136714	PCV2		KP231163	PCV2
EU136715	PCV2		KP231164	PCV2
EU136716	PCV2		KP231165	PCV2
EU136717	PCV2		KP231166	PCV2
EU136718	PCV2		KP231167	PCV2
EU136719	PCV2		KP231168	PCV2
EU136720	PCV2		KP231169	PCV2
EU148503	PCV2		KP231170	PCV2
EU148504	PCV2		KP231171	PCV2
EU148505	PCV2		KP231173	PCV2
EU148506	PCV2		KP313251	PCV2
EU148507	PCV2		KP313252	PCV2
EU257511	PCV2		KP313253	PCV2
EU257512	PCV2		KP313254	PCV2
EU257513	PCV2		KP420181	PCV2
EU257514	PCV2		KP420182	PCV2
EU257515	PCV2		KP420183	PCV2
EU257516	PCV2		KP420184	PCV2
EU274309	PCV2		KP420185	PCV2
EU274310	PCV2		KP420186	PCV2

EU274311	PCV2		KP420187	PCV2
EU274312	PCV2		KP420188	PCV2
EU283329	PCV2		KP420189	PCV2
EU302139	PCV2		KP420190	PCV2
EU302140	PCV2		KP420191	PCV2
EU302141	PCV2		KP420192	PCV2
EU340257	PCV2		KP420193	PCV2
EU340258	PCV2		KP420194	PCV2
EU346945	PCV2		KP420195	PCV2
EU366323	PCV2		KP420196	PCV2
EU366324	PCV2		KP420197	PCV2
EU366325	PCV2		KP420198	PCV2
EU366326	PCV2		KP420199	PCV2
EU391637	PCV2		KP420200	PCV2
EU408780	PCV2		KP420201	PCV2
EU418626	PCV2		KP420202	PCV2
EU418627	PCV2		KP420203	PCV2
EU450584	PCV2		KP637021	PCV2
EU450585	PCV2		KP670418	PCV2
EU450586	PCV2		KP670419	PCV2
EU450587	PCV2		KP670420	PCV2
EU450588	PCV2		KP670421	PCV2
EU450589	PCV2		KP698394	PCV2
EU450590	PCV2		KP698395	PCV2
EU450591	PCV2		KP698396	PCV2
EU450592	PCV2		KP698397	PCV2
EU503031	PCV2		KP698398	PCV2
EU503032	PCV2		KP698399	PCV2
EU503033	PCV2		KP698400	PCV2
EU503034	PCV2		KP698401	PCV2
EU503035	PCV2		KP698402	PCV2
EU503036	PCV2		KP698403	PCV2
EU503037	PCV2		KP698404	PCV2
EU503038	PCV2		KP698405	PCV2
EU503039	PCV2		KP867046	PCV2
EU503040	PCV2		KP867047	PCV2
EU521707	PCV2		KP867048	PCV2
EU521708	PCV2		KP867049	PCV2
EU521709	PCV2		KP867050	PCV2
EU545542	PCV2		KP975430	PCV2
EU545543	PCV2		KP975431	PCV2
EU545544	PCV2		KP975433	PCV2
EU545545	PCV2		KP975434	PCV2
EU545546	PCV2		KP975435	PCV2
EU545547	PCV2		KP975436	PCV2

EU545548	PCV2		KP975437	PCV2
EU545549	PCV2		KP975438	PCV2
EU545550	PCV2		KP975439	PCV2
EU545551	PCV2		KP975440	PCV2
EU555439	PCV2		KP975441	PCV2
EU589463	PCV2		KP975442	PCV2
EU594437	PCV2		KP975443	PCV2
EU594438	PCV2		KP975444	PCV2
EU594439	PCV2		KP975445	PCV2
EU594440	PCV2		KP975446	PCV2
EU647557	PCV2		KP975447	PCV2
EU656143	PCV2		KP975449	PCV2
EU684164	PCV2		KP975450	PCV2
EU747085	PCV2		KP975451	PCV2
EU780073	PCV2		KP975452	PCV2
EU780074	PCV2		KR054744	PCV2
EU886637	PCV2		KR258797	PCV2
EU886638	PCV2		KR816332	PCV2
EU909686	PCV2		KR868575	PCV2
EU909687	PCV2		KT216672	PCV2
EU909688	PCV2		KT216673	PCV2
EU921254	PCV2		KT216674	PCV2
EU921255	PCV2		KT216675	PCV2
EU921256	PCV2		KT216676	PCV2
EU921257	PCV2		KT216677	PCV2
FJ041151	PCV2		KT220420	PCV2
FJ158602	PCV2		KT265712	PCV2
FJ158603	PCV2		KT284886	PCV2
FJ158604	PCV2		KT284887	PCV2
FJ158605	PCV2		KT284888	PCV2
FJ158606	PCV2		KT284889	PCV2
FJ158607	PCV2		KT336598	PCV2
FJ218000	PCV2		KT336599	PCV2
FJ218001	PCV2		KT336600	PCV2
FJ218002	PCV2		KT336601	PCV2
FJ233905	PCV2		KT336602	PCV2
FJ233906	PCV2		KT336603	PCV2
FJ233907	PCV2		KT336604	PCV2
FJ233908	PCV2		KT336605	PCV2
FJ233909	PCV2		KT336606	PCV2
FJ233910	PCV2		KT336607	PCV2
FJ388889	PCV2		KT336608	PCV2
FJ426398	PCV2		KT369067	PCV2
FJ440338	PCV2		KT369068	PCV2
FJ447482	PCV2		KT369069	PCV2

FJ483938	PCV2		KT369070	PCV2
FJ501957	PCV2		KT719404	PCV2
FJ594471	PCV2		KT795287	PCV2
FJ598044	PCV2		KT795288	PCV2
FJ598045	PCV2		KT795289	PCV2
FJ608538	PCV2		KT795290	PCV2
FJ608539	PCV2		KT804910	PCV2
FJ608540	PCV2		KT819159	PCV2
FJ608541	PCV2		KT819160	PCV2
FJ608542	PCV2		KT819161	PCV2
FJ608543	PCV2		KT819162	PCV2
FJ608544	PCV2		KT819163	PCV2
FJ608545	PCV2		KT819164	PCV2
FJ608546	PCV2		KT819165	PCV2
FJ608547	PCV2		KT819166	PCV2
FJ608549	PCV2		KT819167	PCV2
FJ623185	PCV2		KT819168	PCV2
FJ644555	PCV2		KT819169	PCV2
FJ644556	PCV2		KT819170	PCV2
FJ644557	PCV2		KT870146	PCV2
FJ644558	PCV2		KT870147	PCV2
FJ644559	PCV2		KU041848	PCV2
FJ644560	PCV2		KU041849	PCV2
FJ644561	PCV2		KU041850	PCV2
FJ644562	PCV2		KU041851	PCV2
FJ644563	PCV2		KU041852	PCV2
FJ644919	PCV2		KU041853	PCV2
FJ644920	PCV2		KU041854	PCV2
FJ644921	PCV2		KU041855	PCV2
FJ644922	PCV2		KU041856	PCV2
FJ644923	PCV2		KU041857	PCV2
FJ644924	PCV2		KU041858	PCV2
FJ644925	PCV2		KU041859	PCV2
FJ644926	PCV2		KU193766	PCV2
FJ644927	PCV2		KU193767	PCV2
FJ644928	PCV2		KU311010	PCV2
FJ644929	PCV2		KU311011	PCV2
FJ644930	PCV2		KU311012	PCV2
FJ644931	PCV2		KU311013	PCV2
FJ644932	PCV2		KU311014	PCV2
FJ660967	PCV2		KU311015	PCV2
FJ660968	PCV2		KU311016	PCV2
FJ660969	PCV2		KU311017	PCV2
FJ660970	PCV2		KU311018	PCV2
FJ660971	PCV2		KU311019	PCV2

FJ667582	PCV2		KU311020	PCV2
FJ667583	PCV2		KU311021	PCV2
FJ667584	PCV2		KU311022	PCV2
FJ667585	PCV2		KU311023	PCV2
FJ667586	PCV2		KU311024	PCV2
FJ667587	PCV2		KU311025	PCV2
FJ667588	PCV2		KU311026	PCV2
FJ667589	PCV2		KU311027	PCV2
FJ667590	PCV2		KU311028	PCV2
FJ667591	PCV2		KU311029	PCV2
FJ667592	PCV2		KU311030	PCV2
FJ667593	PCV2		KU311031	PCV2
FJ667594	PCV2		KU311032	PCV2
FJ667595	PCV2		KU311033	PCV2
FJ667596	PCV2		KU311034	PCV2
FJ712215	PCV2		KU311035	PCV2
FJ712216	PCV2		KU317472	PCV2
FJ716703	PCV2		KU317473	PCV2
FJ716704	PCV2		KU317474	PCV2
FJ804417	PCV2		KU317475	PCV2
FJ870967	PCV2		KU317476	PCV2
FJ870968	PCV2		KU317477	PCV2
FJ870969	PCV2		KU317478	PCV2
FJ870970	PCV2		KU317479	PCV2
FJ870971	PCV2		KU317480	PCV2
FJ870972	PCV2		KU317481	PCV2
FJ870973	PCV2		KU317482	PCV2
FJ870974	PCV2		KU317483	PCV2
FJ870975	PCV2		KU317484	PCV2
FJ870976	PCV2		KU317485	PCV2
FJ905459	PCV2		KU317486	PCV2
FJ905460	PCV2		KU317487	PCV2
FJ905461	PCV2		KU317488	PCV2
FJ905462	PCV2		KU317489	PCV2
FJ905463	PCV2		KU317490	PCV2
FJ905464	PCV2		KU317491	PCV2
FJ905465	PCV2		KU317492	PCV2
FJ905466	PCV2		KU317493	PCV2
FJ905467	PCV2		KU317494	PCV2
FJ905468	PCV2		KU317495	PCV2
FJ905469	PCV2		KU317496	PCV2
FJ905470	PCV2		KU317497	PCV2
FJ905471	PCV2		KU317498	PCV2
FJ935780	PCV2		KU317499	PCV2
FJ948167	PCV2		KU557355	PCV2

FJ948168	PCV2		KU756237	PCV2
FJ998185	PCV2		KU756238	PCV2
FN398022	PCV2		KU960929	PCV2
FN398023	PCV2		KU960930	PCV2
FN398024	PCV2		KU960931	PCV2
FN398025	PCV2		KU960932	PCV2
FN398027	PCV2		KU960933	PCV2
FR823451	PCV2		KU960934	PCV2
GQ174519	PCV2		KU960935	PCV2
GQ227412	PCV2		KU960936	PCV2
GQ358992	PCV2		KU960937	PCV2
GQ358994	PCV2		KU960938	PCV2
GQ358995	PCV2		KU960939	PCV2
GQ358997	PCV2		KU960940	PCV2
GQ358999	PCV2		KU960941	PCV2
GQ359000	PCV2		KX009480	PCV2
GQ359002	PCV2		KX009481	PCV2
GQ359003	PCV2		KX009482	PCV2
GQ359004	PCV2		KX068219	PCV2
GQ359005	PCV2		KX068220	PCV2
GQ359006	PCV2		KX068221	PCV2
GQ359008	PCV2		KX068222	PCV2
GQ359009	PCV2		KX161663	PCV2
GQ359010	PCV2		KX161664	PCV2
GQ359011	PCV2		KX161665	PCV2
GQ404798	PCV2		KX161666	PCV2
GQ404799	PCV2		KX161667	PCV2
GQ404800	PCV2		KX161668	PCV2
GQ404801	PCV2		KX161669	PCV2
GQ404802	PCV2		KX161670	PCV2
GQ404803	PCV2		KX161671	PCV2
GQ404804	PCV2		KX161672	PCV2
GQ404805	PCV2		KX161673	PCV2
GQ404806	PCV2		KX161674	PCV2
GQ404807	PCV2		KX161675	PCV2
GQ404808	PCV2		KX161676	PCV2
GQ404852	PCV2		KX161677	PCV2
GQ404853	PCV2		KX161678	PCV2
GQ449672	PCV2		KX161679	PCV2
GQ911590	PCV2		KX161680	PCV2
GQ915288	PCV2		KX161681	PCV2
GQ915289	PCV2		KX161682	PCV2
GQ996404	PCV2		KX161683	PCV2
GU001709	PCV2		KX161684	PCV2
GU001710	PCV2		KX161685	PCV2

GU017735	PCV2		KX161686	PCV2
GU049340	PCV2		KX161687	PCV2
GU049341	PCV2		KX161688	PCV2
GU049342	PCV2		KX161689	PCV2
GU083582	PCV2		KX161690	PCV2
GU083583	PCV2		KX161691	PCV2
GU124593	PCV2		KX161692	PCV2
GU233804	PCV2		KX161693	PCV2
GU244506	PCV2		KX161694	PCV2
GU244507	PCV2		KX161695	PCV2
GU247987	PCV2		KX161696	PCV2
GU247988	PCV2		KX161697	PCV2
GU247989	PCV2		KX161698	PCV2
GU247990	PCV2		KX161699	PCV2
GU247991	PCV2		KX247783	PCV2
GU247992	PCV2		KX247784	PCV2
GU252369	PCV2		KX247785	PCV2
GU252370	PCV2		KX247786	PCV2
GU325753	PCV2		KX247787	PCV2
GU325754	PCV2		KX247788	PCV2
GU325755	PCV2		KX247789	PCV2
GU325756	PCV2		KX247790	PCV2
GU325757	PCV2		KX247791	PCV2
GU325758	PCV2		KX247792	PCV2
GU325759	PCV2		KX247793	PCV2
GU325760	PCV2		KX247794	PCV2
GU325761	PCV2		KX247795	PCV2
GU325762	PCV2		KX247796	PCV2
GU325763	PCV2		KX247797	PCV2
GU325764	PCV2		KX247798	PCV2
GU325765	PCV2		KX247799	PCV2
GU325766	PCV2		KX247800	PCV2
GU325767	PCV2		KX247801	PCV2
GU325768	PCV2		KX247803	PCV2
GU325769	PCV2		KX247804	PCV2
GU325770	PCV2		KX247805	PCV2
GU370063	PCV2		KX247806	PCV2
GU370064	PCV2		KX247807	PCV2
GU450327	PCV2		KX247808	PCV2
GU450328	PCV2		KX247809	PCV2
GU450329	PCV2		KX247810	PCV2
GU450330	PCV2		KX247811	PCV2
GU799576	PCV2		KX247812	PCV2
GU808525	PCV2		KX247813	PCV2
GU938302	PCV2		KX247814	PCV2

GU938303	PCV2		KX247815	PCV2
GU938304	PCV2		KX247816	PCV2
HF542107	PCV2		KX247817	PCV2
HM003569	PCV2		KX247818	PCV2
HM003570	PCV2		KX247819	PCV2
HM009336	PCV2		KX247820	PCV2
HM009337	PCV2		KX247821	PCV2
HM009338	PCV2		KX247822	PCV2
HM027579	PCV2		KX247823	PCV2
HM027580	PCV2		KX247824	PCV2
HM038016	PCV2		KX247825	PCV2
HM038017	PCV2		KX247826	PCV2
HM038018	PCV2		KX247827	PCV2
HM038019	PCV2		KX247828	PCV2
HM038020	PCV2		KX247829	PCV2
HM038021	PCV2		KX247830	PCV2
HM038022	PCV2		KX247831	PCV2
HM038023	PCV2		KX247832	PCV2
HM038024	PCV2		KX247833	PCV2
HM038025	PCV2		KX247834	PCV2
HM038026	PCV2		KX247835	PCV2
HM038027	PCV2		KX247836	PCV2
HM038028	PCV2		KX247837	PCV2
HM038029	PCV2		KX247838	PCV2
HM038030	PCV2		KX247839	PCV2
HM038031	PCV2		KX247840	PCV2
HM038032	PCV2		KX247841	PCV2
HM038033	PCV2		KX247842	PCV2
HM038034	PCV2		KX247843	PCV2
HM102350	PCV2		KX247844	PCV2
HM142894	PCV2		KX298473	PCV2
HM142895	PCV2		KX298474	PCV2
HM142896	PCV2		KX352153	PCV2
HM142897	PCV2		KX352154	PCV2
HM142898	PCV2		KX352155	PCV2
HM142899	PCV2		KX352156	PCV2
HM142900	PCV2		KX352157	PCV2
HM161710	PCV2		KX352158	PCV2
HM161711	PCV2		KX352159	PCV2
HM623764	PCV2		KX352160	PCV2
HM641752	PCV2		KX352161	PCV2
HM776437	PCV2		KX352445	PCV2
HM776438	PCV2		KX668489	PCV2
HM776439	PCV2		KX668490	PCV2
HM776440	PCV2		KX668491	PCV2

HM776441	PCV2		KX668492	PCV2
HM776442	PCV2		KX814347	PCV2
HM776443	PCV2		KX814348	PCV2
HM776444	PCV2		KX814349	PCV2
HM776445	PCV2		KX814350	PCV2
HM776446	PCV2		KX814351	PCV2
HM776447	PCV2		KX814352	PCV2
HM776448	PCV2		KX826907	PCV2
HM776449	PCV2		KX826908	PCV2
HM776450	PCV2		KX828213	PCV2
HM776451	PCV2		KX828214	PCV2
HM776452	PCV2		KX828215	PCV2
HM776453	PCV2		KX828216	PCV2
HQ113117	PCV2		KX828217	PCV2
HQ113118	PCV2		KX828218	PCV2
HQ113119	PCV2		KX828219	PCV2
HQ113120	PCV2		KX828220	PCV2
HQ113121	PCV2		KX828221	PCV2
HQ148879	PCV2		KX828222	PCV2
HQ202944	PCV2		KX828223	PCV2
HQ202945	PCV2		KX828224	PCV2
HQ202946	PCV2		KX828225	PCV2
HQ202947	PCV2		KX828226	PCV2
HQ202948	PCV2		KX828227	PCV2
HQ202949	PCV2		KX828228	PCV2
HQ202950	PCV2		KX828229	PCV2
HQ202951	PCV2		KX828230	PCV2
HQ202952	PCV2		KX828231	PCV2
HQ202953	PCV2		KX828232	PCV2
HQ202954	PCV2		KX828233	PCV2
HQ202955	PCV2		KX828234	PCV2
HQ202956	PCV2		KX828235	PCV2
HQ202957	PCV2		KX828236	PCV2
HQ202958	PCV2		KX828237	PCV2
HQ202959	PCV2		KX828238	PCV2
HQ202960	PCV2		KX828239	PCV2
HQ202961	PCV2		KX828240	PCV2
HQ202962	PCV2		KX828241	PCV2
HQ202963	PCV2		KX828581	PCV2
HQ202964	PCV2		KX831475	PCV2
HQ202965	PCV2		KX831477	PCV2
HQ202966	PCV2		KX831478	PCV2
HQ202967	PCV2		KX831479	PCV2
HQ202968	PCV2		KX831480	PCV2
HQ202969	PCV2		KX831481	PCV2

HQ202970	PCV2		KX831482	PCV2
HQ202971	PCV2		KX831483	PCV2
HQ202972	PCV2		KX844823	PCV2
HQ202973	PCV2		KX844824	PCV2
HQ231328	PCV2		KX844825	PCV2
HQ231329	PCV2		KX845692	PCV2
HQ378158	PCV2		KX845693	PCV2
HQ378159	PCV2		KX845694	PCV2
HQ378160	PCV2		KX845695	PCV2
HQ378161	PCV2		KX855982	PCV2
HQ395019	PCV2		KX855983	PCV2
HQ395020	PCV2		KX855984	PCV2
HQ395021	PCV2		KX856425	PCV2
HQ395022	PCV2		KX865092	PCV2
HQ395023	PCV2		KX865093	PCV2
HQ395024	PCV2		KX865094	PCV2
HQ395025	PCV2		KX867818	PCV2
HQ395026	PCV2		KX867819	PCV2
HQ395027	PCV2		KX867820	PCV2
HQ395028	PCV2		KX867821	PCV2
HQ395029	PCV2		KX894318	PCV2
HQ395030	PCV2		KX894319	PCV2
HQ395031	PCV2		KX894321	PCV2
HQ395032	PCV2		KX904945	PCV2
HQ395033	PCV2		KX904946	PCV2
HQ395034	PCV2		KX904947	PCV2
HQ395035	PCV2		KX904948	PCV2
HQ395036	PCV2		KX904949	PCV2
HQ395037	PCV2		KX904950	PCV2
HQ395038	PCV2		KX904951	PCV2
HQ395039	PCV2		KX904952	PCV2
HQ395040	PCV2		KX904953	PCV2
HQ395041	PCV2		KX928993	PCV2
HQ395042	PCV2		KX928994	PCV2
HQ395043	PCV2		KX928995	PCV2
HQ395044	PCV2		KX928996	PCV2
HQ395045	PCV2		KX928997	PCV2
HQ395046	PCV2		KX928998	PCV2
HQ395047	PCV2		KX928999	PCV2
HQ395048	PCV2		KX929000	PCV2
HQ395049	PCV2		KX929001	PCV2
HQ395050	PCV2		KX929002	PCV2
HQ395051	PCV2		KX929003	PCV2
HQ395052	PCV2		KX929004	PCV2
HQ395053	PCV2		KX929005	PCV2

HQ395054	PCV2		KX929006	PCV2
HQ395055	PCV2		KX929007	PCV2
HQ395056	PCV2		KX929008	PCV2
HQ395057	PCV2		KX929009	PCV2
HQ395058	PCV2		KX929010	PCV2
HQ395059	PCV2		KX929011	PCV2
HQ395060	PCV2		KX929012	PCV2
HQ395061	PCV2		KX929013	PCV2
HQ402903	PCV2		KX929014	PCV2
HQ591365	PCV2		KX929015	PCV2
HQ591366	PCV2		KX929016	PCV2
HQ591367	PCV2		KX929017	PCV2
HQ591368	PCV2		KX929018	PCV2
HQ591370	PCV2		KX929019	PCV2
HQ591371	PCV2		KX960916	PCV2
HQ591372	PCV2		KX960917	PCV2
HQ591373	PCV2		KX960918	PCV2
HQ591374	PCV2		KX960919	PCV2
HQ591375	PCV2		KX960920	PCV2
HQ591376	PCV2		KX960921	PCV2
HQ591377	PCV2		KX960922	PCV2
HQ591378	PCV2		KX960923	PCV2
HQ591379	PCV2		KX960924	PCV2
HQ591380	PCV2		KX960925	PCV2
HQ591381	PCV2		KX960926	PCV2
HQ650833	PCV2		KX960927	PCV2
HQ693093	PCV2		KX960928	PCV2
HQ713495	PCV2		KX960929	PCV2
HQ738639	PCV2		KX960930	PCV2
HQ738640	PCV2		KX960931	PCV2
HQ738641	PCV2		KX960932	PCV2
HQ831519	PCV2		KX960933	PCV2
HQ831520	PCV2		KX960934	PCV2
HQ831521	PCV2		KX960935	PCV2
HQ831522	PCV2		KX960936	PCV2
HQ831523	PCV2		KX960937	PCV2
HQ831524	PCV2		KX960938	PCV2
HQ831525	PCV2		KX960939	PCV2
HQ831526	PCV2		KX960940	PCV2
HQ831527	PCV2		KX960941	PCV2
HQ831528	PCV2		KX960942	PCV2
HQ831529	PCV2		KX960943	PCV2
HQ831530	PCV2		KX960944	PCV2
HQ831531	PCV2		KX960945	PCV2
HQ831532	PCV2		KX960946	PCV2

HQ831533	PCV2		KX960947	PCV2
HQ831534	PCV2		KX960948	PCV2
HQ831535	PCV2		KX960949	PCV2
HQ831536	PCV2		KX981602	PCV2
HQ831537	PCV2		KX981603	PCV2
HQ831538	PCV2		KX981604	PCV2
HQ831539	PCV2		KY084478	PCV2
HQ831540	PCV2		KY126311	PCV2
JF272497	PCV2		KY126312	PCV2
JF272498	PCV2		KY126313	PCV2
JF272499	PCV2		KY126314	PCV2
JF290418	PCV2		KY126315	PCV2
JF317565	PCV2		KY126317	PCV2
JF317566	PCV2		KY211020	PCV2
JF317567	PCV2		KY305198	PCV2
JF317568	PCV2		KY305199	PCV2
JF317569	PCV2		KY305200	PCV2
JF317570	PCV2		KY305201	PCV2
JF317571	PCV2		KY305202	PCV2
JF317572	PCV2		KY305203	PCV2
JF317573	PCV2		KY305204	PCV2
JF317574	PCV2		KY347898	PCV2
JF317575	PCV2		KY347899	PCV2
JF317576	PCV2		KY388466	PCV2
JF317577	PCV2		KY388467	PCV2
JF317578	PCV2		KY388468	PCV2
JF317579	PCV2		KY388469	PCV2
JF317580	PCV2		KY388470	PCV2
JF317581	PCV2		KY388471	PCV2
JF317582	PCV2		KY388472	PCV2
JF317583	PCV2		KY388473	PCV2
JF317584	PCV2		KY388474	PCV2
JF317585	PCV2		KY388475	PCV2
JF317586	PCV2		KY388476	PCV2
JF317587	PCV2		KY388477	PCV2
JF317588	PCV2		KY388478	PCV2
JF317589	PCV2		KY388479	PCV2
JF682791	PCV2		KY425814	PCV2
JF682792	PCV2		KY425815	PCV2
JF682793	PCV2		KY440165	PCV2
JF682794	PCV2		KY440166	PCV2
JF690911	PCV2		KY509295	PCV2
JF690912	PCV2		KY509296	PCV2
JF690913	PCV2		KY569375	PCV2
JF690914	PCV2		KY569376	PCV2

JF690915	PCV2		KY569377	PCV2
JF690916	PCV2		KY569378	PCV2
JF690917	PCV2		KY569379	PCV2
JF690918	PCV2		KY569380	PCV2
JF690919	PCV2		KY569381	PCV2
JF690920	PCV2		KY569382	PCV2
JF690921	PCV2		KY569383	PCV2
JF690922	PCV2		KY613025	PCV2
JF690923	PCV2		KY613026	PCV2
JF718784	PCV2		KY613027	PCV2
JF827599	PCV2		KY613028	PCV2
JF899334	PCV2		KY613029	PCV2
JF928002	PCV2		KY613030	PCV2
JF928003	PCV2		KY613031	PCV2
JF928004	PCV2		KY613032	PCV2
JF928005	PCV2		KY652744	PCV2
JF928006	PCV2		KY652745	PCV2
JN119255	PCV2		KY652746	PCV2
JN119256	PCV2		KY652747	PCV2
JN133304	PCV2		KY659550	PCV2
JN176181	PCV2		KY659551	PCV2
JN388690	PCV2		KY659552	PCV2
JN615187	PCV2		KY659553	PCV2
JN639856	PCV2		KY659554	PCV2
JN639857	PCV2		KY677756	PCV2
JN660055	PCV2		KY677757	PCV2
JQ002671	PCV2		KY810319	PCV2
JQ002672	PCV2		KY810320	PCV2
JQ181585	PCV2		KY810321	PCV2
JQ181586	PCV2		KY810322	PCV2
JQ181587	PCV2		KY810323	PCV2
JQ181588	PCV2		KY810324	PCV2
JQ181589	PCV2		KY810325	PCV2
JQ181590	PCV2		KY940520	PCV2
JQ181591	PCV2		KY940521	PCV2
JQ181592	PCV2		KY940522	PCV2
JQ181593	PCV2		KY940523	PCV2
JQ181594	PCV2		KY940524	PCV2
JQ181595	PCV2		KY940525	PCV2
JQ181596	PCV2		KY940526	PCV2
JQ181597	PCV2		KY940527	PCV2
JQ181598	PCV2		KY940528	PCV2
JQ181599	PCV2		KY940529	PCV2
JQ181600	PCV2		KY940530	PCV2
JQ181601	PCV2		KY940531	PCV2

JQ181602	PCV2		KY940532	PCV2
JQ181603	PCV2		KY940533	PCV2
JQ181604	PCV2		KY940534	PCV2
JQ181605	PCV2		KY940535	PCV2
JQ181606	PCV2		KY940536	PCV2
JQ181607	PCV2		KY940537	PCV2
JQ390467	PCV2		KY940538	PCV2
JQ413808	PCV2		KY940539	PCV2
JQ653449	PCV2		KY940540	PCV2
JQ692110	PCV2		KY940541	PCV2
JQ806749	PCV2		KY940542	PCV2
JQ809462	PCV2		KY940543	PCV2
JQ809463	PCV2		LC004732	PCV2
JQ809464	PCV2		LC004733	PCV2
JQ955679	PCV2		LC004734	PCV2
JQ994268	PCV2		LC004735	PCV2
JQ994269	PCV2		LC004736	PCV2
JQ994270	PCV2		LC004737	PCV2
JX099780	PCV2		LC004738	PCV2
JX099781	PCV2		LC004739	PCV2
JX099782	PCV2		LC004740	PCV2
JX099783	PCV2		LC004741	PCV2
JX099784	PCV2		LC004742	PCV2
JX099785	PCV2		LC004743	PCV2
JX099786	PCV2		LC004744	PCV2
JX193799	PCV2		LC004745	PCV2
JX204386	PCV2		LC004746	PCV2
JX274295	PCV2		LC004747	PCV2
JX294717	PCV2		LC004748	PCV2
JX406419	PCV2		LC004749	PCV2
JX406420	PCV2		LC004750	PCV2
JX406421	PCV2		LC004751	PCV2
JX406422	PCV2		LC004752	PCV2
JX406423	PCV2		LC004753	PCV2
JX406424	PCV2		LC004754	PCV2
JX406426	PCV2		LC008134	PCV2
JX506730	PCV2		LC008135	PCV2
JX512853	PCV2		LC008136	PCV2
JX512854	PCV2		LC008137	PCV2
JX512855	PCV2		LC008138	PCV2
JX512856	PCV2		LC008139	PCV2
JX512857	PCV2		LC008140	PCV2
JX512858	PCV2		LC008141	PCV2
JX512859	PCV2		LC008142	PCV2
JX512860	PCV2		MF142260	PCV2

JX519293	PCV2		MF142261	PCV2
JX534236	PCV2		MF142262	PCV2
JX534237	PCV2		MF142263	PCV2
JX535296	PCV2		MF142264	PCV2
JX535297	PCV2		MF142265	PCV2
JX678978	PCV2		MF142266	PCV2
JX679498	PCV2		MF142267	PCV2
JX912914	PCV2		MF142268	PCV2
JX912915	PCV2		MF142269	PCV2
JX945575	PCV2		MF142270	PCV2
JX945576	PCV2		MF142271	PCV2
JX945577	PCV2		MF142272	PCV2
JX948768	PCV2		MF142273	PCV2
JX948769	PCV2		MF142274	PCV2
JX948770	PCV2		MF142275	PCV2
JX948771	PCV2		MF142276	PCV2
JX948772	PCV2		MF278777	PCV2
JX948773	PCV2		MF278778	PCV2
JX948774	PCV2		MF278779	PCV2
JX948775	PCV2		MF616413	PCV2
JX948776	PCV2		MF616414	PCV2
JX948777	PCV2		MF616415	PCV2
JX948778	PCV2		MF616416	PCV2
JX948779	PCV2		MF616417	PCV2
JX948780	PCV2		MF616418	PCV2
JX948781	PCV2		MF616419	PCV2
JX948782	PCV2		MF616420	PCV2
JX948783	PCV2		MF616421	PCV2
JX948784	PCV2		MF616422	PCV2
JX948785	PCV2		MF616423	PCV2
JX948786	PCV2		MF616424	PCV2
JX982219	PCV2		MF616425	PCV2
JX982220	PCV2		MF616426	PCV2
JX982221	PCV2		MF616427	PCV2
JX982222	PCV2		MF616428	PCV2
JX982223	PCV2		MF616429	PCV2
JX982224	PCV2		MF616432	PCV2
JX982225	PCV2		MF926252	PCV2
JX982226	PCV2		MF964235	PCV2
JX982227	PCV2		MF981845	PCV2
JX982228	PCV2		MF981846	PCV2
KC153106	PCV2		MG744311	PCV2
KC188796	PCV2		NC_005148	PCV2
KC249977	PCV2			

Table S4. Accession numbers of wildtype PCV variants used for analyses
